# Isoreticular Chemistry and Applications of Supramolecularly Assembled Copper–Adenine Porous Materials

**DOI:** 10.1021/acs.inorgchem.3c02708

**Published:** 2023-11-01

**Authors:** Sandra Mena-Gutiérrez, Jon Pascual-Colino, Garikoitz Beobide, Oscar Castillo, Ainara Castellanos-Rubio, Antonio Luque, Ekain Maiza-Razkin, Jon Mentxaka, Sonia Pérez-Yáñez

**Affiliations:** † Departamento de Química Orgánica e Inorgánica, Facultad de Ciencia y Tecnología, Universidad del País Vasco/Euskal Herriko Unibertsitatea, UPV/EHU, Apartado 644, E-48080 Bilbao, Spain; ‡ BCMaterials, Basque Center for Materials, Applications and Nanostructures, UPV/EHU Science Park, E-48940 Leioa, Spain; § Departamento de Genética, Antropología física y Fisiología animal, Facultad de Medicina, Universidad del País Vasco/Euskal Herriko Unibertsitatea, UPV/EHU, E-48940 Leioa, Spain; ∥ Ikerbasque, Basque Foundation for Science; E-48011, Bilbao, Spain; ⊥ Biobizkaia Research Institute, E-480903 Barakaldo, Bizkaia Spain; # Departamento de Bioquímica y Biología Molecular, UPV-EHU, E-48940 Leioa, Bizkaia Spain

## Abstract

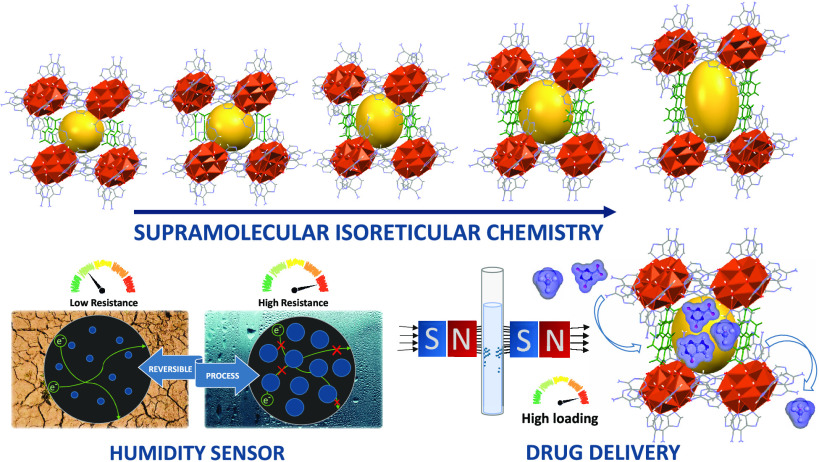

The useful concepts of reticular chemistry, rigid and predictable metal nodes together with strong and manageable covalent interactions between metal centers and organic linkers, have made the so-called metal–organic frameworks (MOFs) a flourishing area of enormous applicability. In this work, the extension of similar strategies to supramolecularly assembled metal–organic materials has allowed us to obtain a family of isoreticular compounds of the general formula [Cu_7_(μ-adeninato-κ*N3*:κ*N9*)_6_(μ_3_-OH)_6_(μ-OH_2_)_6_](OOC-R-COO)·*n*H_2_O (R: ethylene-, acetylene-, naphthalene-, or biphenyl-group) in which the rigid copper–adeninato entities and the organic dicarboxylate anions are held together not by covalent interactions but by a robust and flexible network of synergic hydrogen bonds and π–π stacking interactions based on well-known supramolecular synthons (SMOFs). All compounds are isoreticular, highly insoluble, and water-stable and show a porous crystalline structure with a **pcu** topology containing a two-dimensional (2D) network of channels, whose dimensions and degree of porosity of the supramolecular network are tailored by the length of the dicarboxylate anion. The partial loss of the crystallization water molecules upon removal from the mother liquor produces a shrinkage of the unit cell and porosity, which leads to a color change of the compounds (from blue to olive green) if complete dehydration is achieved by means of gentle heating or vacuuming. However, the supramolecular network of noncovalent interactions is robust and flexible enough to reverse to the expanded unit cell and color after exposure to a humid atmosphere. This humidity-driven breathing behavior has been used to design a sensor in which the electrical resistance varies reversibly with the degree of humidity, very similar to the water vapor adsorption isotherm of the SMOF. The in-solution adsorption properties were explored for the uptake and release of the widely employed 5-fluorouracil, 4-aminosalycilic acid, 5-aminosalycilic acid, and allopurinol drugs. In addition, cytotoxicity activity assays were completed for the pristine and 5-fluorouracil-loaded samples.

## Abstract 2

Isoreticular chemistry principles applied to flexible porous supramolecular materials with applications on moisture sensoring and drug molecule capture/release in aqueous solutions.

## Introduction

Reticular chemistry,^1,2^ the rational combination of inorganic nodes and organic linkers to afford extended framework structures with predictable and precise architectural arrangements, has made metal–organic frameworks (MOFs) the most fruitful and the fastest growing area of inorganic chemistry.^3^ In fact, the structural CSD database records more than 100,000 crystal structures of MOFs.^4^ The robustness and predictability of the node–linker coordinate bonds in MOFs allow obtaining porous materials whose cavities show the appropriate characteristics (size, surface area, and chemical nature) to be successfully applied in areas such as gas storage and separation,^5^ catalysis,^6^ toxic chemical removal,^7^ water adsorption,^8^ clean energy,^9^ and biomedicine.^10^

In this sense, the development of the concept of isoreticular chemistry played a key role in the success of these materials as it provided to the experimentalist a straightforward control on the pore size and inner surface chemistries while retaining the network topology.^11−16^ This approach has been fruitful in providing families of isoreticular MOFs such as the pioneering IRMOFs of Yaghi et al.,^11^ the Zr-based UiO-66, -67, and -68 series prepared by Lillerud and co-workers,^17^ the large analogues of MIL-88 from the Feréy and Serre laboratory,^18^ and the PCN-61, -66, -68, and -610 MOFs based on hexacarboxylate ligands and developed by Zhou.^19^ These coordination bond-based porous materials have shaken the area of porous materials pushing up the porosity limits to previously unthinkable values.^20^ Despite the success of MOFs in delivering highly porous materials, the chemistry of MOFs also presents some drawbacks such as chemical or mechanical instability, time-consuming and expensive synthetic methods, and the use of toxic solvents among others that cannot always be overcome.^21^ Therefore, there has been a great interest in finding alternative porous materials, among which covalent organic frameworks (COFs),^22,23^ hydrogen organic frameworks (HOFs),^24,25^ and even the less studied supramolecular metal–organic frameworks (SMOFs) stand out.^26−28^

SMOFs are porous materials in which the crystal structure is governed by noncovalent forces such as hydrogen bonds and π–π stacking interactions.^29,30^ These materials are obtained through milder processes and their structure is more adaptable and can afford reversible assembling/disassembling processes.^31,32^ Although supramolecular interactions are weaker than covalent bonds, SMOFs usually involve more than one supramolecular linking interaction between adjacent discrete building units. This feature means that many of the structures of SMOFs are stable enough to be applied in areas similar to MOFs, such as gas adsorption,^33^ catalysis,^34^ and the incorporation of toxic adsorptives.^35^

In this work, a previously reported flexible SMOF with the formula [Cu_7_(μ-adeninato-κ*N3*:κ*N9*)_6_(μ_3_-OH)_6_(μ-OH_2_)_6_](OOC–C_6_H_4_–COO)^36^ has been employed as a source of inspiration to create an isoreticular family replacing the terephthalate anion by other dicarboxylate anions of different lengths. In this compound, the assembly of the structural units takes place, in addition to the electrostatic forces, by recurrent π–π interactions between the adeninato ligands of the copper(II)-based cationic entities and also by means of hydrogen bonds involving the carboxylate groups of terephthalate anions and a HO–Cu–OH fragment of the complex cations. We expect this synthon to be strong enough to direct the supramolecular building into the same topology, regardless of the dicarboxylate anion. To test this hypothesis, five dicarboxylate organic anions were employed to obtain their supramolecular assemblies with the heptameric entity: ethylenedicarboxylate, also named as fumarate (**1a, 1b**), acetylenedicarboxylate (**2a, 2b**), terephthalate (**3**, incorporated for comparative purposes), naphthalene-2,6-dicarboxylate (**4a, 4b**), and biphenyl-4,4′-dicarboxylate (**5)**. Compounds marked as **a** correspond to fully hydrated samples, while the **b** label corresponds to partially dehydrated samples. We will show how the previously mentioned synthon is retained in all cases giving rise to an isoreticular family of compounds in which we can analyze the effect of the length of the dicarboxylate anion on the porosity, structural flexibility, and water adsorption capacity. Finally, potential applications of these compounds as humidity sensors and as drug loading/releasing materials in aqueous solutions are also tested.

## Methods

### Chemicals

Copper(II) nitrate trihydrate (Cu(NO_3_)_2_·3H_2_O, Sigma-Aldrich, 98%), fumaric acid (C_4_H_4_O_4_, Sigma-Aldrich, >99%), acetylenedicarboxylic acid (C_4_H_2_O_4_, Sigma-Aldrich, 95%), naphthalene-2,6-dicarboxylic acid (C_12_H_8_O_4_, Sigma-Aldrich, 95%), biphenyl-4,4′-dicarboxylic acid (C_14_H_10_O_4_, Sigma-Aldrich, 97%), adenine (C_5_H_5_N_5_, Sigma-Aldrich, >99%), and methanol (CH_3_OH, Scharlau, >99% v/v) were the chemicals used as commercially obtained.

#### Synthesis of [Cu_7_(μ-adeninato)_6_(μ_3_-OH)_6_(μ-OH_2_)_6_](fumarate)·∼22H_2_O (**1a**) and [Cu_7_(μ-adeninato)_6_(μ_3_-OH)_6_(μ-OH_2_)_6_](fumarate)·∼16H_2_O (**1b**)

A mixture of 20 mL of methanol–water (1:1) containing 0.0810 g of adenine (0.60 mmol), heated to 60 °C, was added to a solution of 0.1223 g (0.50 mmol) of Cu(NO_3_)_2_·3H_2_O in 10 mL of distilled water. The resulting mixture (pH ∼ 4) was stirred while NaOH was added dropwise until pH ∼ 9. On the other hand, 0.1160 g of fumaric acid (1.00 mmol) was dissolved at room temperature in 15 mL of distilled water. This solution was basified with NaOH until pH ∼ 11 and added to the first one. The pH was adjusted to ∼9.2 by adding dilute HNO_3_ solution (1:3) dropwise. The final purple solution was covered with parafilm and allowed to stand at room temperature. After 3–4 days, blue cubic crystals of compound **1a** appeared. A single crystal was selected, removed from the solution, covered with Paratone oil, and used for single-crystal X-ray data collection (**1a**). To check the purity of the samples, polycrystalline samples were introduced into a capillary with their mother liquor to obtain their powder X-ray diffraction (PXRD). Crystals from another synthesis with the same procedure were filtered for ∼20 min and selected for study by single-crystal X-ray diffraction (**1b**). Data will show that during the filtration process in air, the crystals of **1a** undergo partial dehydration to give **1b**, although preserving the original color and crystalline nature. Yield: *ca.* 80%. IR features (cm^–1^; KBr pellets): 3450vs, 3350vs, 3310s, 1640vs, 1600m, 1550s, 1460vs, 1400vs, 1370s, 1280s, 1190vs, 1140vs, 1040m, 930w, 800m, 740m, 670s, 520vs, and 470m.

#### Synthesis of [Cu_7_(μ-adeninato)_6_(μ_3_-OH)_6_(μ-OH_2_)_6_](acetylenedicarboxylate)·∼22H_2_O (**2a**) and [Cu_7_(μ-adeninato)_6_(μ_3_-OH)_6_(μ-OH_2_)_6_](acetylenedicarboxylate)·∼17H_2_O (**2b**)

Compound **2a** was synthesized by the above-described method, but by using 0.1206 g of acetylenedicarboxylic acid (0.90 mmol). After 4–5 days, blue cubic crystals of compound **2a** appeared. After filtration, partially dehydrated crystals (**2b**) were obtained. Yield: *ca.* 60%. IR features (cm^–1^; KBr pellets): 3420vs, 3350m, 2130m, 1640vs, 1600m, 1550s, 1500m, 1460vs, 1340s, 1280m, 1150s, 1200s, 1030m, 940w, 780m, 740m, 660m, 550m, and 450m.

#### Synthesis of [Cu_7_(μ-adeninato)_6_(μ_3_-OH)_6_(μ-OH_2_)_6_](naphthalene-2,6-dicarboxylate)·∼32H_2_O (**4a**)

The above-described procedure for compounds **1a** and **2a** was used for the preparation of compound **4a**, but it employed 0.1405 g of naphthalene-2,6-dicarboxylic acid (0.65 mmol). After 6–7 days, blue square single crystals of **4a** were obtained. The air-drying of the crystals produces its partial dehydration (**4b**) but, in this case, the crystals become cracked and get a matte color appearance. Unfortunately, its structural characterization by single-crystal diffraction was precluded, but the mesogravimetric measurements indicate *ca*. 27 crystallization water molecules per formula (see Figure S4 and Table S3 of the Supporting Information). Yield: *ca.* 85%. Main IR features (cm^–1^; KBr pellets): 3440vs, 3340w, 3190vs, 1640vs, 1600m, 1540s, 1490m, 1460vs, 1400vs, 1340s, 1270m, 1140s, 1200s, 1040m, 930w, 790m, 740m, 650m, 550m, and 450m.

When crystals of fumarate (**1**)-, acetylenedicarboxylate (**2**)-, and naphthalene-2,6-dicarboxylate (**4**)-containing compounds are completely dehydrated at 100 °C or under vacuum, they lose brightness and their color changes from blue to olive green. Powder X-ray diffraction indicates a loss of crystallinity with broader peaks shifted to higher 2θ value data (Figure S5 and S6). This bulk dehydrated sample adsorbs ambient moisture upon exposure to a humid atmosphere for 24 h, reverting to its original blue color and powder X-ray diffraction pattern.

#### Synthesis of [Cu_7_(μ-adeninato)_6_(μ_3_-OH)_6_(μ-OH_2_)_6_](biphenyl-4,4′-dicarboxylate)·∼44H_2_O (**5**)

All our attempts to obtain this compound by a direct mixture of the starting materials were unsuccessful. For that, a few block-like crystals of **5** suitable for X-ray diffraction were isolated after 2 weeks using a test tube diffusion technique in which over the aquo-methanolic solution containing the copper(II) salt (0.0489 g, 0.20 mmol) and adenine (0.0603, 0.45 mmol) mixture, an aqueous solution of the organic acid (0.1090 g, 0.45 mmol) is layered carefully. Yield: *ca.* 5%.

### Characterization

The purity of the bulk samples was assessed by powder X-ray diffraction (PXRD), thermogravimetric analysis (TGA), and Fourier-transform infrared (FTIR) spectroscopy. Due to the partial dehydration and structural change taking place upon the removal from the mother liquid, X-ray powder diffraction was performed over samples introduced in a Lindemann capillary filled with the synthesis mother liquid. A Rigaku Smartlab automatic diffractometer with a capillary fixation head was used and the diffraction data were collected in continuous rotation in the range 3° < 2θ < 65°. Routine PXRD measurements were performed on a Phillips X’PERT diffractometer (equipped with Cu–Kα radiation, λ = 1.5418 Å) over the range 5 < 2θ < 70° with a step size of 0.02°, a variable automatic divergence slit, and an acquisition time of 2.5 s per step at 293 K. The X-ray thermodiffraction patterns of compounds were carried out with a Bruker D8 Advance diffractometer equipped with a copper tube in the range 5° < 2θ < 30° with a step of 0.016°, an acquisition time of 0.7 s, and a heating rate of 0.166 °C·s^–1^. TGA was performed on a Mettler Toledo TGA/SDTA851 thermal analyzer in a synthetic air (80% N_2_, 20% O_2_) flux of 50 cm^3^·min^–1^ from room temperature to 600 °C with a heating rate of 5 °C·min^–1^ and a sample amount of about 10–20 mg per run. FTIR spectra of the samples (KBr pellets) were recorded at a resolution of 4 cm^–1^ in the 4000–500 cm^–1^ region using a FTIR 8400S Shimadzu spectrometer. Variable-temperature magnetic susceptibility measurements were performed using a standard Quantum Design PPMS magnetometer while cooling from 300 to 2 K at 1 kOe. Magnetization as a function of field (H) was measured using the same magnetometer in −50 ≤ H/kOe ≤ 50 at 2 K after cooling the sample in zero field.

The single-crystal X-ray diffraction data for structure determination were collected on Agilent Technologies Supernova diffractometers (λ MοK_α_= 0.71073 for **1a**, **1b**, **2b**, and **5**; λ Cu Kα= 1.54184 Å for **2a** and **4a**). The data reduction was done with the CrysAlisPro program.^37^ Crystal structures were solved by direct methods using SIR92^38^ or SUPERFLIP^39^ (**5**) and refined by full-matrix least-squares on *F*^2^ including all reflections (SHELXS).^40^ All calculations for these structures were performed using the WINGX crystallographic software package programs.^41,42^ The crystal structure of some of these compounds shows disorder on the positions of some of the adeninato ligands and the aromatic ring of the dicarboxylic ligands. The disorder was modeled by distributing the disordered atoms over two positions and fixing the sum of their occupation factors to one. The high disorder that some solvent molecules present precluded their modeling and, as a consequence, the electron density was subtracted from the reflection data by the SQUEEZE method^43^ as implemented in PLATON.^44^ Details of the structure determination and refinement of all compounds are summarized in Table S4 of the Supporting Information.

Water vapor sorption isotherms were performed using an automated gravimetric analyzer (Aquadyne DVS, Quantachrome Instruments) with nitrogen 6.0 as the carrier gas and an equilibrium criterion corresponding to 0.0004% of mass change per minute at a given relative humidity. Before the experiments, the samples were outgassed under vacuum at 30 °C for 8 h.

Magnetic sustentation adsorption quantification was performed using a dipole electromagnet (Newport Pagnell England Electromagnet Type C sourced by a Hewlett-Packard 6655A system DC power supply) to determine the critical magnetic field at which the particles are detached from the electromagnet pole for the unloaded and loaded samples. Each measurement was repeated 5 times to provide the corresponding associated error. The calibration of the technique required samples of the porous materials with a known adsorbed mass that were previously determined by ^1^H-NMR spectroscopy using a Bruker AVANCE 5OO (one-bay; 500 MHz) spectrometer at 293 K after duplicating the adsorption procedure using D_2_O instead of water and using an internal standard.

### Cytotoxicity Studies

The human colorectal cancer line HCT116 (ATCC–CCL247) was employed. Cells were seeded at 3000 cells per well in 96-well plates and incubated at 37 °C and 5% CO_2_ in Dulbecco’s modified Eagle’s medium (DMEM) supplemented with 10% FBS and antibiotics. After overnight incubation, the compounds were added to the cells and the cells were collected for crystal violet staining at 8, 24, 48, and 72 h incubation time points. Subsequently, the incubated cells were fixed with paraformaldehyde and stained with 0.1% crystal violet. The staining was washed 3 times; 10% acetic acid was added, and plates were incubated by shaking for 20 min. Absorbance was measured at 590 nm and relative proliferation was calculated.

## Results and Discussion

### Heptameric Cation

**Figure 1:**
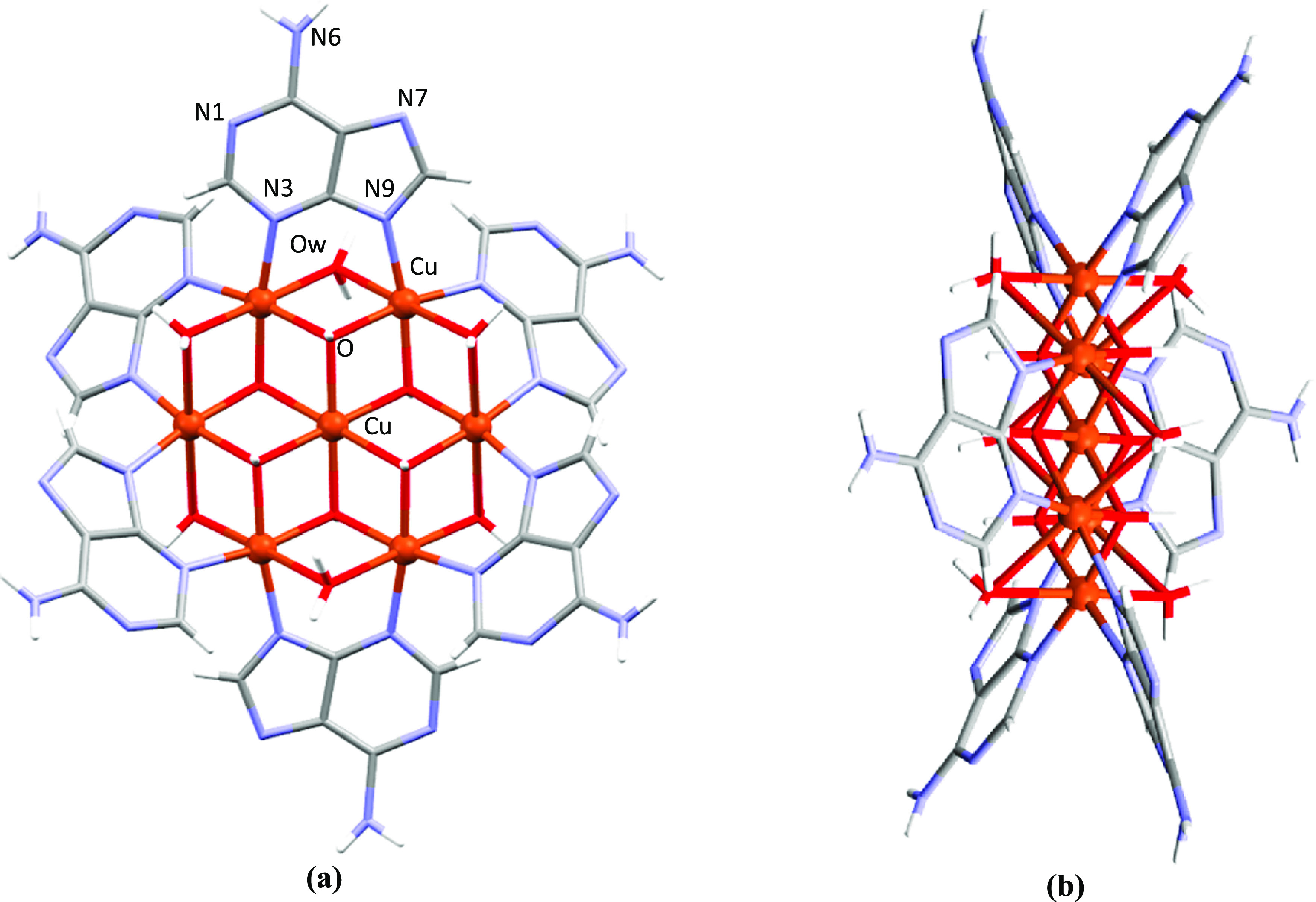
Views of the heptanuclear entity [Cu_7_(μ-adeninato)_6_(μ_3_-OH)_6_(μ-OH_2_)_6_]^2+^: (a) front and (b) side. Color code: H, white; C, grey; N, blue; O, red; and Cu, orange.

The basic building unit of all of the compounds is a cationic wheel-shaped [Cu_7_(μ-adeninato)_6_(μ_3_-OH)_6_(μ-H_2_O)_6_]^2+^ entity in which a central [Cu(OH)_6_]^4–^ core is connected to six outer copper(II) metal centers through μ_3_-hydroxido bridges in a radial and planar arrangement.^36,45,46^ The external copper atoms are doubly bridged by semicoordinated water molecules and by peripheral adeninato ligands which exhibit a bidentate μ-*κN3*:*κN9* coordination mode (Figure 1). All of the metal centers present an octahedral geometry with the usual Jahn–Teller tetragonal elongation, which is more pronounced for the external copper(II) atoms than for the inner one because of the rigidity of the heptanuclear entity (Tables S4–S9).

### Crystal Packings of Compounds

**Figure 2:**
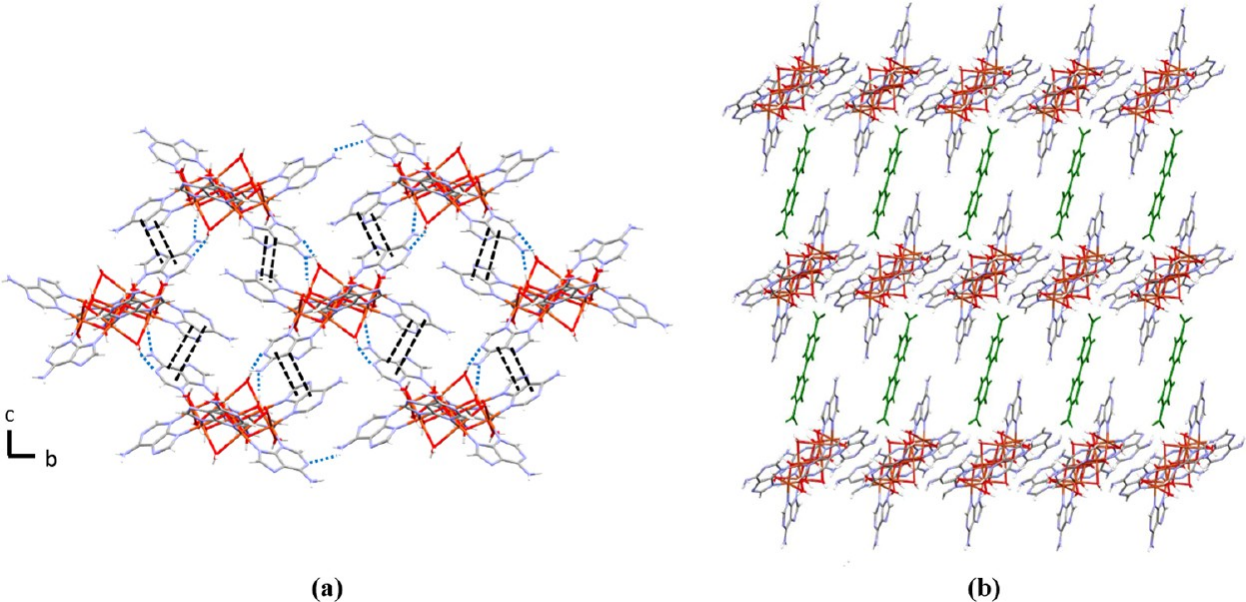
(a) Supramolecular cationic layer formed by heptameric complexes; π–π interaction (double black line) and hydrogen bonds (dashed blue line). (b) Insertion of the dicarboxylate dianion (green) in the crystal structure of **5**.

In the crystal structure of all compounds, each heptameric cation is surrounded by four other ones, interacting with them through adeninato–adeninato offset face-to-face π–π stackings (Figure 2a). This intercomplex interaction is reinforced by a hydrogen-bonded R_2_^2^(8) ring formed by two O–H···N hydrogen bonding interactions between the pyrimidinic N1 and exocyclic N6 nitrogen atoms of the Watson–Crick face of an adeninato ligand as acceptor and a HO–Cu–OH_2_ fragment of a cationic unit as donor. These supramolecular interactions give rise to cationic layers from which the two adeninato ligands not involved in them hang perpendicularly. The dicarboxylate anions are inserted perpendicular to the cationic sheets and sited between two pendant adeninato ligands (Figures 2b and S10).

**Figure 3:**
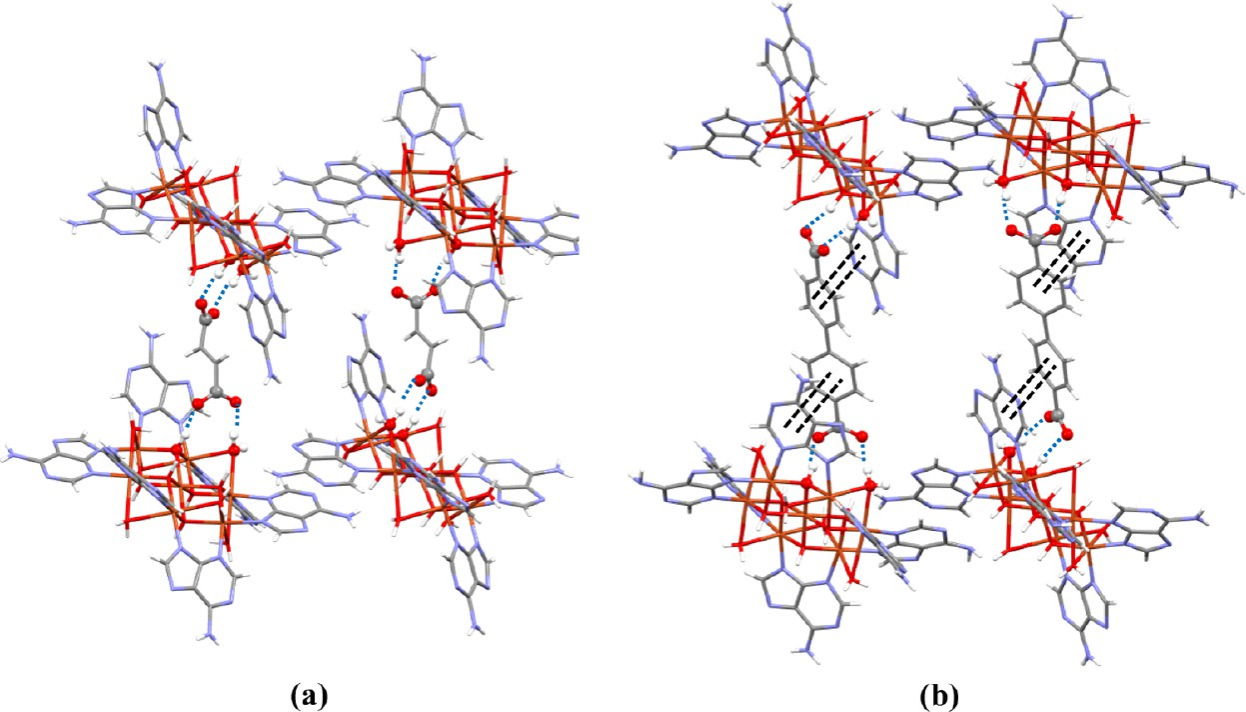
Details of the supramolecular anion–cation interactions in compounds: (a) **1a** and (b) **5**.

Each carboxylate group of the organic anion is attached to the nearest cationic layer by two O_anion_···H–O_cation_ hydrogen bonds, one with a hydroxide group and the other with a water molecule coordinated to the same copper atom to form a supramolecular R_2_^2^(8) synthon. Additionally, the aromatic rings of the terephthalate (**3**) naphthalene-2,6-dicarboxylate (**4a**) and biphenyl-4,4′-dicarboxylate anions (**5**) establish offset face-to-face π–π interactions with both adeninato moieties (Figures 3, S11 and Tables S10–S15).

**Figure 4:**
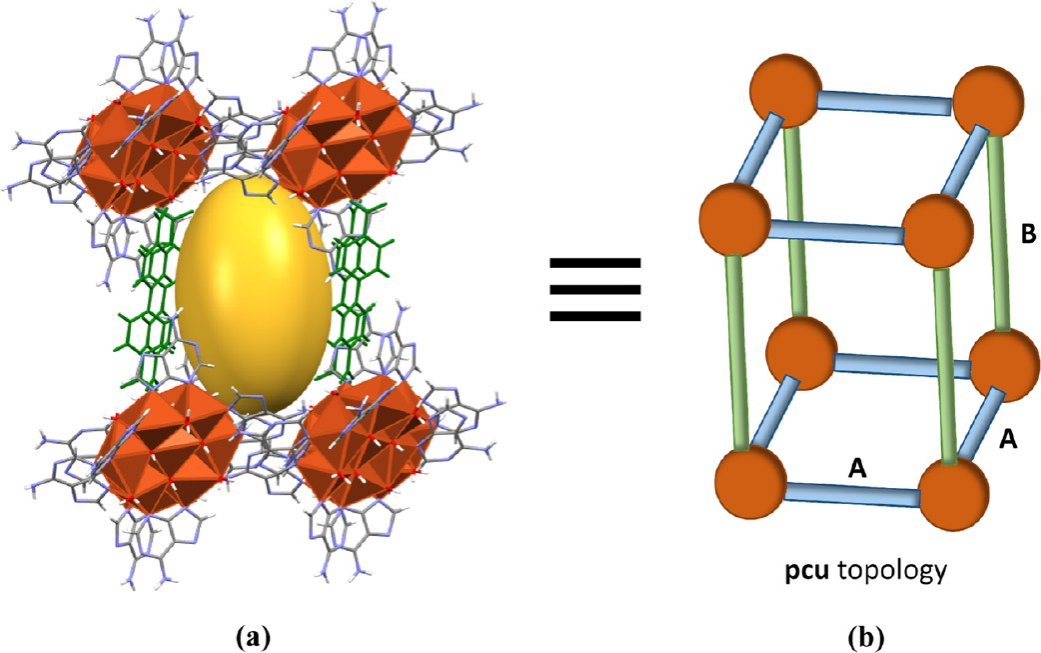
(a) Crystallographic and (b) schematic supramolecular box in compound **5**.

The above-described supramolecular interactions generate rectangular structural boxes (Figure 4), where the eight vertices are occupied by heptameric units (orange color), the edges of two opposite faces are defined by the π–π adeninato–adeninato interactions (blue A edge), and the edges that connect these opposite faces are formed by the adeninato–dicarboxylate–adeninato supramolecular synthon (green B edge). The distance between the heptameric clusters along the adeninato–adeninato interaction is *ca.* 12.1 Å in all cases but the longitudinal distance corresponding to the adeninato–dicarboxylate–adeninato interactions increases with the length of the dicarboxylate anion ranging from 13.5 Å for **1a** to 18.6 Å for **5**. The void in the inner of these molecular boxes (yellow color) is filled by the crystallization water molecules, and it increases with the increasing dicarboxylate anion size (Table S17 and Figures S12, S13).

**Figure 5:**
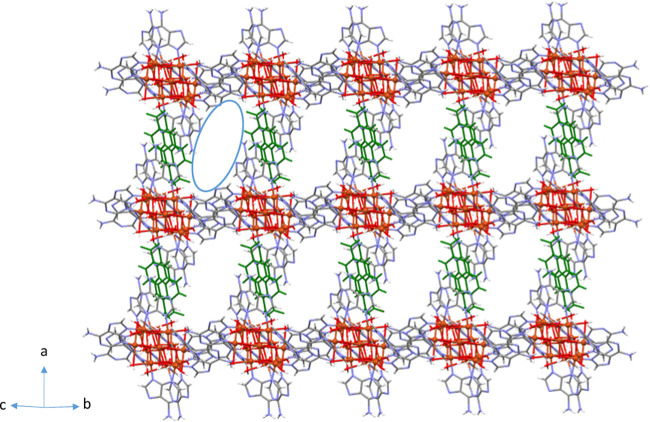
Image showing the channels for compound **5**.

**Figure 6:**
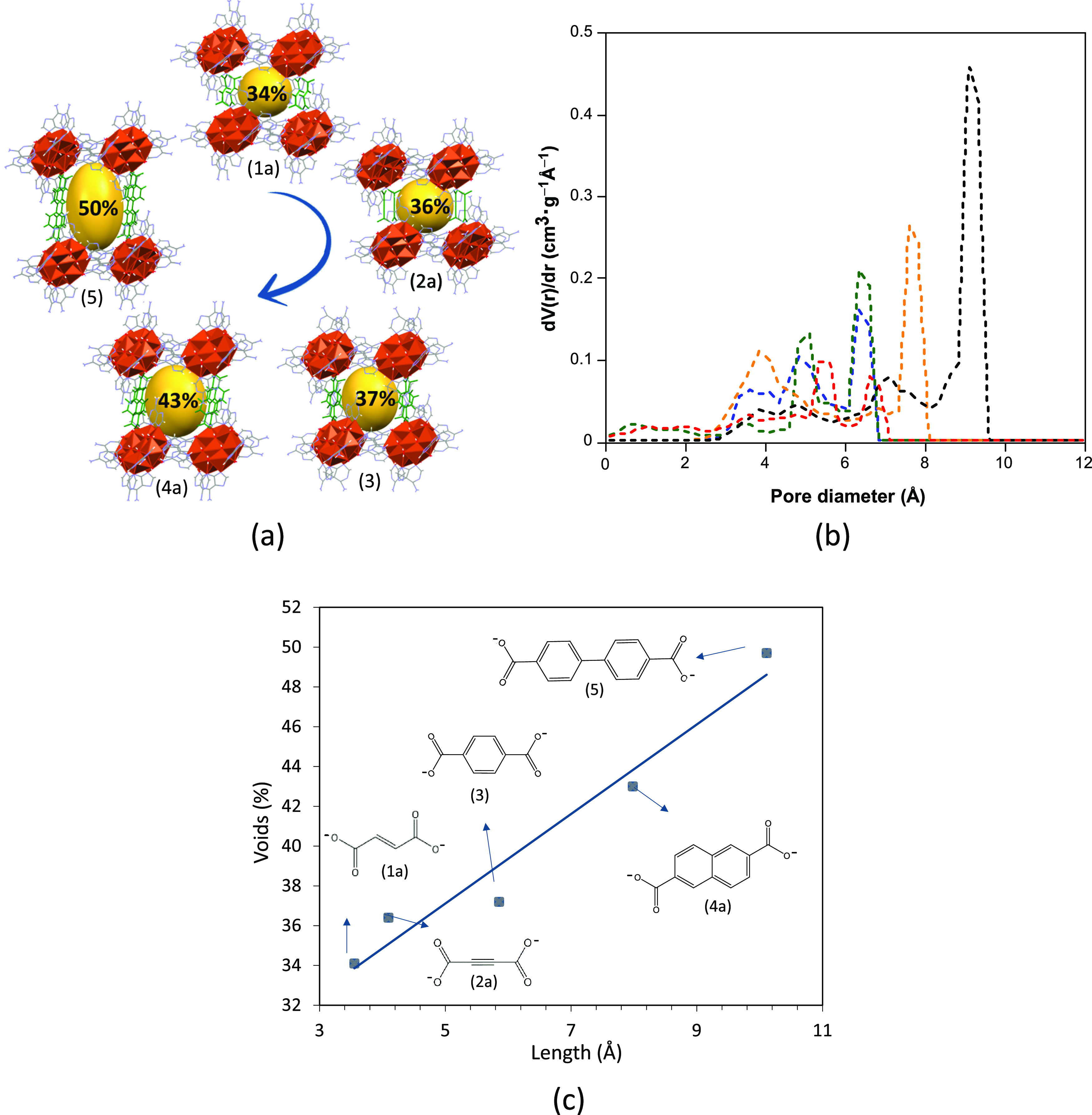
(a) Schematic relationship between the anion size and degree of porosity of the isoreticular compounds. (b) Pore size distribution for compounds **1a** (green), **2a** (blue), **3** (red), **4a** (orange), and **5** (black). (c) Linear correlation between the length of the dicarboxylate counterion and the void percentage.

The packing of these supramolecular boxes gives a porous network (Figure 5) that can be described as a 6-connected uninodal net with a **pcu** α-Po primitive cubic topology and a (4^12^·6^3^) point symbol considering the heptameric clusters as nodes and the π–π and the hydrogen bonding interactions as linkers.^47^ The overall topology resembles the reticular **pcu** architecture of the well-known IRMOF metal–organic framework series sustained by covalent cluster–linker interactions.^48^ As a result, the overall supramolecular architecture contains a 2D network of cross-linked channels formed by great pores (the inner cavity of the molecular boxes) connected by thinner corridors (the walls of the molecular boxes). The inner void of the molecular boxes and potential porosity of the crystal structures (calculated by PLATON) increase with the size of the organic anion, and an almost linear relationship between the porosity degree and the distance between the carbon atoms in the carboxylate groups of the anions is observed (Figure 6).

A computational analysis^49^ of porosity reveals an accessible surface area value of these voids, which gradually increases from 409 m^2^·g^–1^ for **1a** up to 1153 m^2^·g^–1^ for **5**, with a total pore volume of 0.257 and 0.476 cm^3^·g^–1^, respectively (Table S17). The pore size distribution provided by this analysis also corroborates the increase of the main cavity with respect to the dicarboxylate anion length (Figure 6b). Note that the flexible nature of these compounds precludes the analysis of the porosity through the measurement of the gas adsorption isotherms and thus the computational method can be regarded as a suitable approach for the comparison of the porous features of each compound.

### Water Adsorption Properties and Structural Flexibility

The crystallographic resolution of fumarate- and acetylenedicarboxylate-containing compounds shows that their partial dehydratation entails a significant reduction in the volume of the unit cell and the degree of porosity. The effect seems to be more drastic in the case of the naphthalene-2,6-dicarboxylate compound in which the crystals are cracked during dehydration. In all cases, when the water molecules are fully eliminated by the effect of temperature or by vacuum, the porosity drastically decreases with the X-ray diffractogram showing a broadening of the peaks and a significant displacement to higher angular values (indicative of a contraction of the unit cell, as shown in Figures S5 and S6). Indeed, the activated samples adsorb neither N_2_ (77 K) nor CO_2_ (298 K). However, the supramolecular networks are robust and flexible enough for recovering the crystalline original open structure when the outgassed samples are stored for 24 h in a humidifier with a humidity of *ca.* 90%, the crystallization water molecules are regained, and the powder X-ray diffraction patterns match those corresponding to the pristine hydrated **1a**, **2a**, and **4a** compounds. The dehydration/rehydration process is accompanied by a color change of the samples, from dark olive green for the dehydrated samples (probably because the semicoordinated water molecules are also totally or partially evacuated) to blue for the hydrated ones.

**Figure 7:**
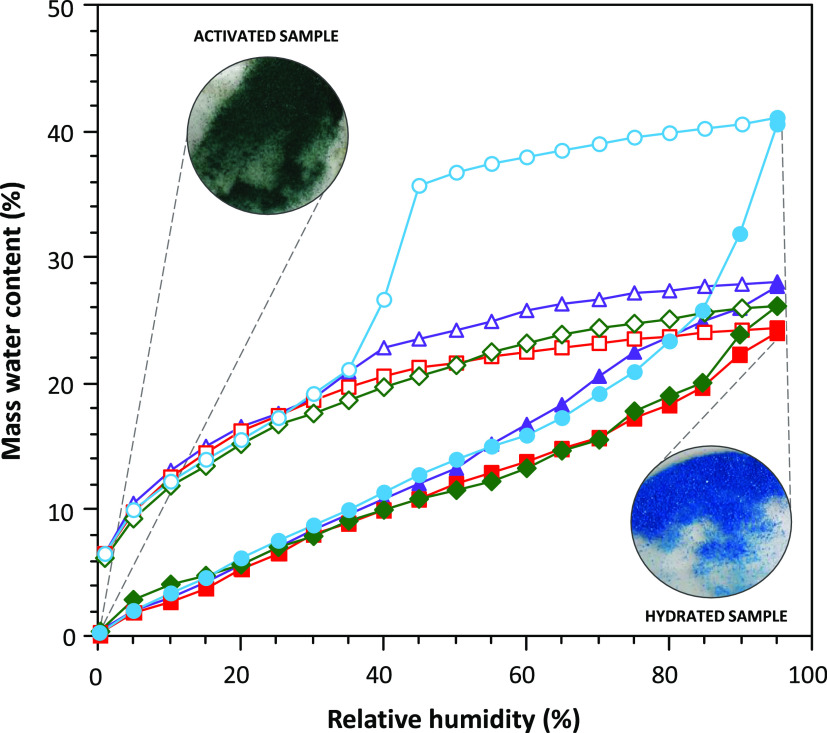
First water cycling of adsorption/desorption isotherms at 20 °C for an activated sample of **1b** (purple triangle), **2b** (red square), **3** (green rhombus), and **4b** (blue circle).

Figure 7 shows the water vapor adsorption–desorption curves for previously outgassed samples of the compounds. All compounds show a water adsorption pronounced hysteresis curve that does not close at low humidity values. There is still 5–6% of water retained, at zero humidity value. This amount of unreleased water is close to that expected for the coordination water molecules (5.2–6.1%). The source of this broad hysteresis curve comes from the structural flexibility that these compounds show, in which the pores shrink upon the removal of the solvent molecules and expand upon their adsorption. It means that unlike in many MOFs, there is neither a sudden adsorption step as the water adsorption must be followed by a structural rearrangement, nor there is that sudden step in the desorption curve for the same reason.

**Figure 8:**
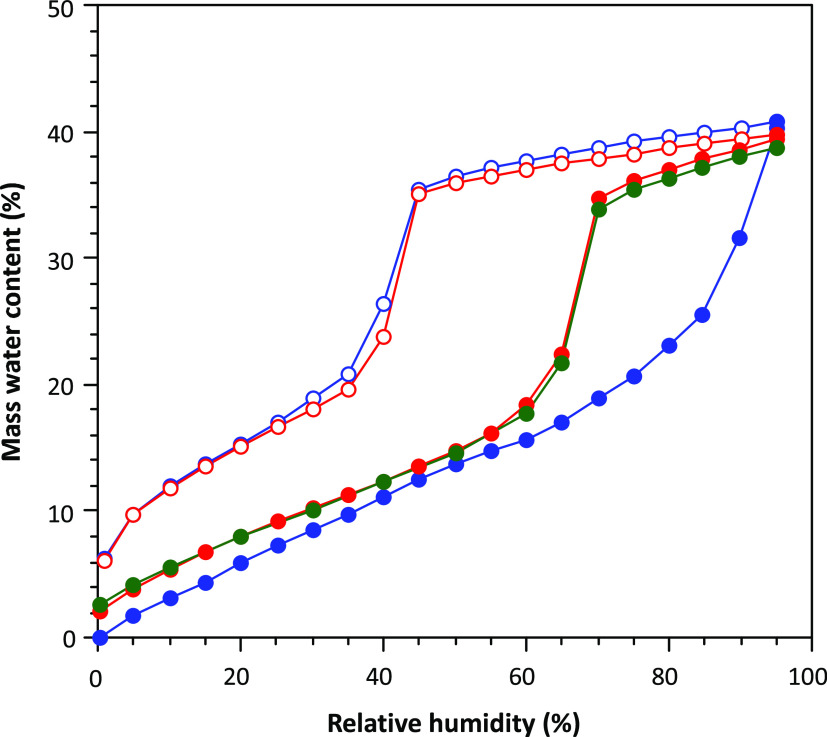
Consecutive adsorption/desorption cycling at 20 °C for **4b** (1st cycle: blue dots; 2nd cycle: red dots; and 3rd cycle: green dots).

However, compound **4b** shows a different desorption curve with the presence of a sudden drop around humidity values of 40%. The adsorption curve follows the same tendency described for the rest of the compounds with the exception of a greater amount of adsorbed water at its maximum (*ca*. 40%), which agrees with the greater void volume present in the crystal structure of the full hydrated compound **4a**. The sharp decrease in the desorption curve is characteristic of well-defined rigid voids. It seems that the elimination of the coordination water molecules induces a structural transformation, but the removal of the crystallization water molecules does not produce strong structural changes, contrary to the rest of the compounds tested in this work. This explanation becomes more evident when we analyze the second and third adsorption/desorption cycles (Figure 8) where the sample is not further activated, and the coordination water molecules are not removed. In these new cycles, we can observe that sharp step not only in the desorption curve but also in adsorption. Apart from that, there is a great mismatch between the adsorption step (humidity: ∼70%) and the desorption step (humidity: ∼40%) that is usually explained as due to a bottleneck effect. The crystal structure of this compound shows huge ellipsoidal pores (diameter: *ca.* 8.0 Å) that are connected by relatively narrow windows (*ca.* 4.5 Å). It means that the area surrounding these windows will adsorb a moderate amount of water at lower humidity values, as the water molecules interact more strongly, but the large pores to be filled require higher humidity values. However, when performing the desorption process, the water molecules present in the pores are released only when the water molecules in these necks/windows are released. We are aware that this is probably not the only contributing factor because the pores do not seem so big, and probably the crystal structure flexibility is also playing a significant role in determining the adsorption/desorption curve features.

**Figure 9:**
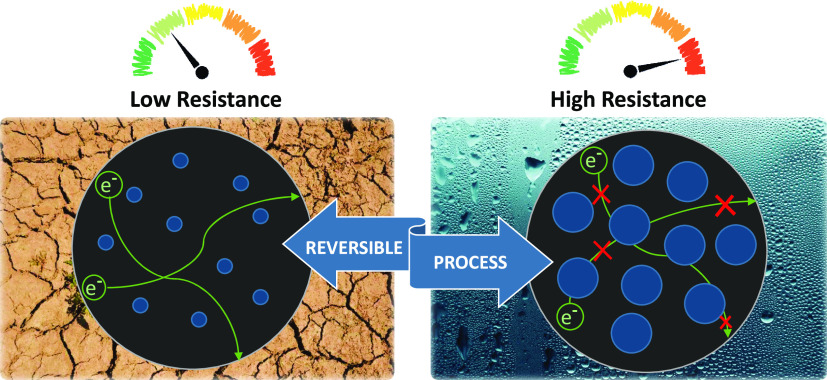
Scheme of the humidity-sensing composite pellet under different humidity conditions.

Taking advantage of the water adsorption capacity and the related structural breathing behavior of these compounds, in which the water incorporation into the materials leads to an expansion of the pores, and as a consequence, of the cell, we focused on the design of a humidity sensor based on this stimuli-response property. For this purpose, we envisaged a composite material shaped in the form of a disc (Figure 9) whose electrical conductivity would change according to the humidity values. The composite consisted of 80% of the porous SMOF material (in this case, the acetylenedicarboxylate compound was selected because of its smaller hysteresis curve that would imply a lower uncertainty in the provided humidity value). However, as these SMOFs lack meaningful electrical conductivity, we incorporated 10% of carbon black to provide the necessary conductivity and also 10% of poly(tetrafluoroethylene) (PTFE) as a binder to ensure the material stability of the disc.^50^

**Figure 10:**
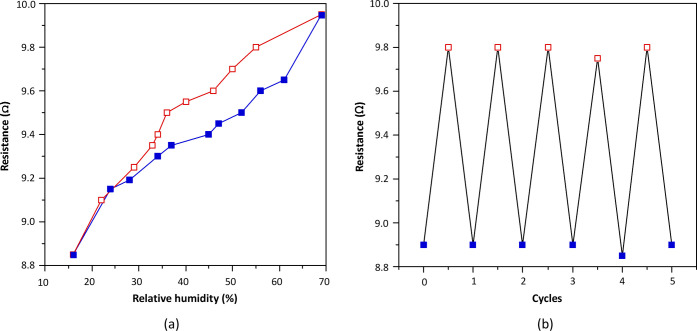
(a) Resistance variation during the water adsorption (red) and desorption (blue) processes of the composite pellet. (b) Cyclability of the adsorption/desorption process between 18 and 65% relative humidity.

The electrical conductivity change taking place on the disc comes from the degree of percolation between the carbon black particles. The latter depends on the particle size of the compound, which is controlled by the atmospheric humidity values. The conductivity measurement was performed by applying two silver paste contacts on opposite edges of the disc. The results shown in Figure 10 indicate that the conductivity values follow the same trend observed in the water adsorption isotherm. The stability and reversibility of the composite disc as a humidity sensor were also ensured by means of various cycles in which it was exposed to 15 and 70% relative humidity. There are no changes in the values for at least the first five cycles. Outside this humidity range, the response of the disc shows a wider hysteresis cycle (Figure S21), which would hinder its possible use as a sensor.

### Drug Loading and Delivering in Aqueous Media

At this point, encouraged by the achieved control on the porosity of this family of SMOFs and also by their great chemical stability and insolubility in aqueous media, we decided to check their viability as drug carriers incorporating different pharmaceutically active molecules such as the antitumoral 5-fluorouracil (5-FU), the antibiotic 4-aminosalicylic acid (4-ASA), 5-aminosalicylic acid (5-ASA), which is used to treat inflammatory bowel diseases, and the antiarthritic allopurinol (ALLO). The loading of these drugs in aqueous solution was monitored by means of a novel magnetic sustentation procedure recently developed by us.^32,51,52^ The method allows to obtain the amount of guest molecules incorporated by a paramagnetic porous material in aqueous solution, and it is based on the determination of the critical magnetic field (H) that is necessary to retain the particles of this material attached to the poles of a magnet (Figure S24). There is a linear correlation (eq 1) between the mass percentage of the loading molecule by the porous material and the critical H, in such a way that the greater the deviation with respect to the critical magnetic value of the pristine material (without loading), the greater the mass of the captured guest molecules.
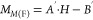
1where *M*_*M(*F*)*_ corresponds to the captured mass of the adsorbate in the material, *H* is the critical magnetic field determined from magnetic sustentation experiments, and A′ and B′ are constant values which must be previously determined by means of a calibration procedure that requires ^1^H-NMR measurements using an internal standard (*t*-BuOH) and repeating the adsorption procedure but using D_2_O.

The naphthalene-2,6-dicarboxylate compound (**4**) was employed because it is the compound that can be achieved in pure form, showing higher porosity and bigger voids. This compound as well as all of those based on this heptameric copper–adeninato entity presents a ferrimagnetic ordering at low temperatures (see Section S8 of the Supporting Information) but in the paramagnetic regime at room temperature. The calibration of the magnetic sustentation device was made using three adsorptive molecules: methanol (MeOH), propan-2-ol (i-PrOH), and glucose (GLU). For that, 50 mg of both **4b** and the adsorptive were placed in 2 mL of water and kept with gentle shaking for 24 h at 25 °C. Then, magnetic measurements were performed to determine the critical magnetic field at which the adsorbate@material particles are detached from the pole of the electromagnet. Simultaneously, the amount of captured adsorbate was determined by measuring the remaining concentration of the adsorptive in solution after the adsorption procedure. For this purpose, ^1^H-NMR measurements were performed on 1 mL of the liquid phase by adding 100 μL of a 5% tert-butanol heavy water solution. The characteristic signals of the adsorptive and tert-butanol were employed to quantify the amount of adsorptive remaining in the solution after 24 h of adsorption, taking into account the difference with the blank (Figures S25–S27).

**Figure 11:**
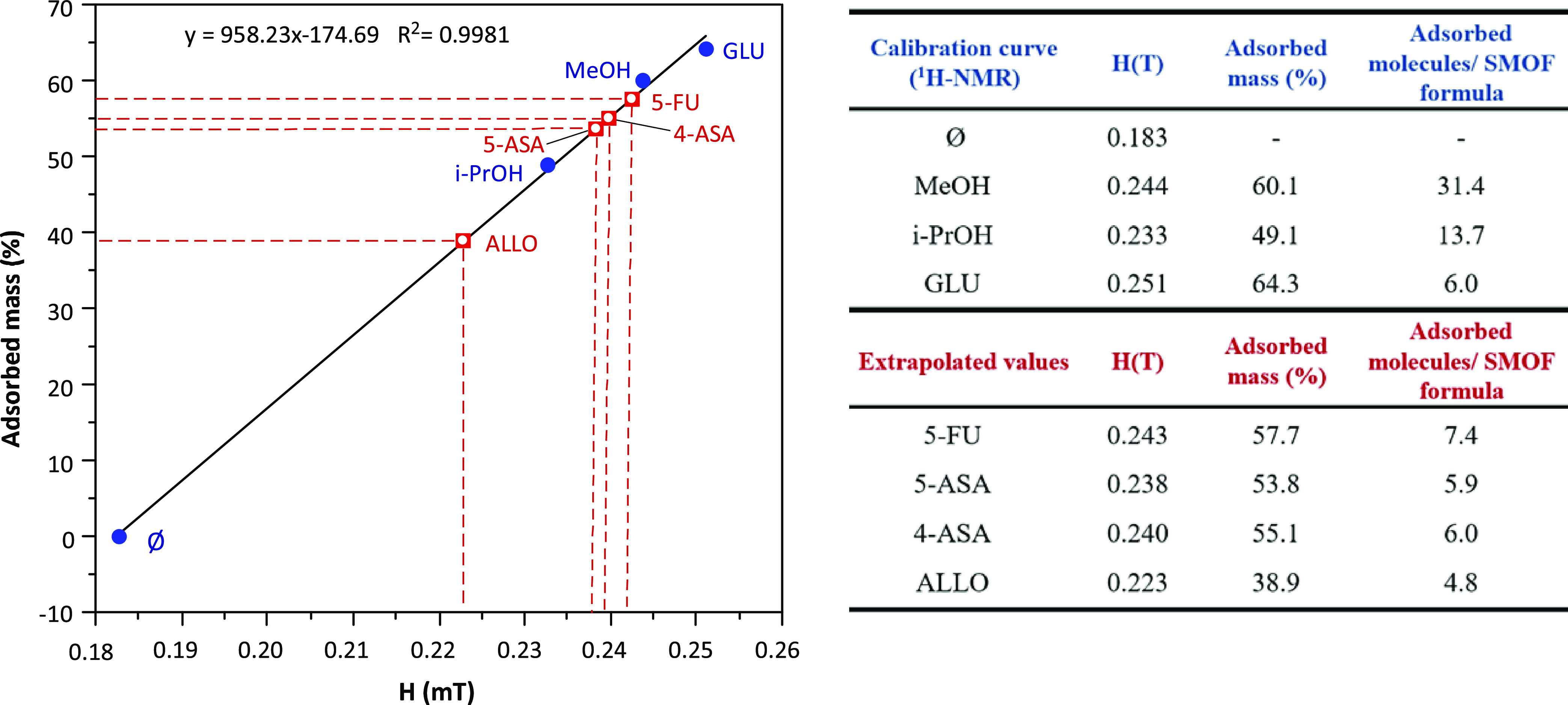
Linear dependence of adsorbed mass (%) versus critical magnetic field. Calibration curves were obtained by ^1^H-NMR spectroscopy for MeOH, iPrOH, and GLU, altogether with the extrapolated values for the selected drug molecules. Ø represents the pristine sample of compound **4a**.

The plot of the adsorbed mass vs H (Figure 11) reveals a direct proportionality that allows us to define the calibration line that will be employed later on for the quantification of the uptake of the drug molecules.

The magnetic sustentation technique offers several advantages toward more conventional techniques (ultraviolet–visible (UV–vis) spectroscopy, chromatography, or ^1^H-NMR) such as the direct quantification of the adsorbate mass incorporated into the material. In addition to that, the technique does not present any dependence on the nature of the adsorbate. In fact, the low solubility of selected drug molecules hinders or even precludes their adsorption quantification based on ^1^H-NMR or any procedure that relies on the quantification of the drug molecules remaining in solution. The extrapolation of the critical magnetic field in the calibration line provides the loading percentage for these drug molecules, which ranges between 38 and 58% (see table within Figure 11).

The incorporation of the drug molecules is also evidenced in the IR spectra and PXRD patterns. The IR spectra show additional signals coming from the incorporated adsorbate molecules (see Figure S28). On the other hand, the PXRD patterns do not reproduce that of the original compound **4** when removed from the mother liquid (closed form) but they are closer to that of the compound **4** when still in the mother liquid (open form), although still significant differences can be found probably due to the incorporation of the drug molecules within the channels of the SMOF (Figure S29). The most intense and first appearing peak is located at a slightly smaller 2θ angle (5.30–5.38 vs 5.75° for the drug-captured samples and the open form of **4**, *i.e*., **4a**, respectively). Among the drug-captured samples, there are also differences in terms of the positions of the peaks and intensity values. The good quality of the diffraction patterns has allowed us to complete a Le-Bail refinement, based on the unit cell parameters of the open form of compound **4**. The results indicate that the unit cell is able to expand even more than when immersed in pure water, accounting for the great flexibility provided by the supramolecular interactions that sustain the crystal structure of these SMOFs.

In fact, when analyzing the size, volume, and cross-section of all of the employed adsorptive molecules (Figures S30 and S31), which usually are a key factor on the adsorption capacity of the rigid porous materials (most MOFs), no correlation is observed (Figure S32).^53,54^ However, analyzing the number of captured drug molecules per formula unit of the SMOF, the obtained values are close to six, which can be related to the local 6-fold symmetry of the heptameric unit with six adeninato peripheral ligands ready to establish supramolecular interactions, especially if the incorporated molecules are of planar nature and hydrogen bonding-capable as are the selected drug molecules.

**Figure 12:**
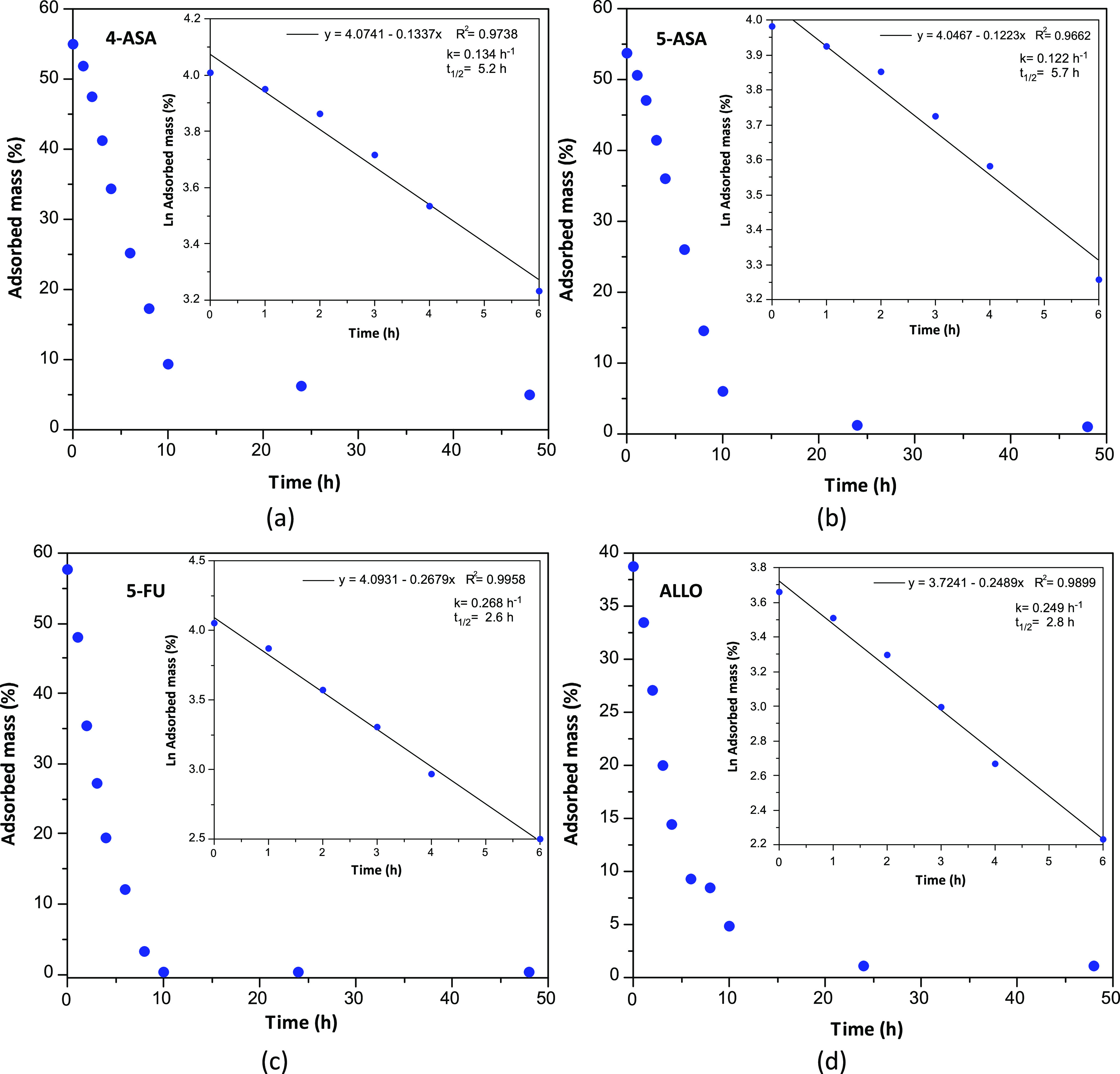
Desorption isotherms in the water solution of the **drugs@4** samples: Inset: fitting to first-order kinetics considering the first 6 h of the desorption process.

After assessing the drug uptake for compound **4a**, we decided to analyze the desorption kinetics of the drug-loaded samples at 35 °C using the same magnetic sustentation technique. For this purpose, 50 mg of drug-loaded samples of the SMOFs were placed in 5 mL of water and the critical magnetic field was measured at different times to determine the evolution of the drug release. The aqueous media was replaced after each measurement. The desorption curves indicate that half of the initially loaded drug is released in the first 2–3 h for 5-FU and ALLO and 6–7 h for 4-ASA and 5-ASA, following first-order kinetics (Figure 12).

### Cytotoxicity Assay of 5-FU@4 on the HCT116 Cell Culture

**Figure 13:**
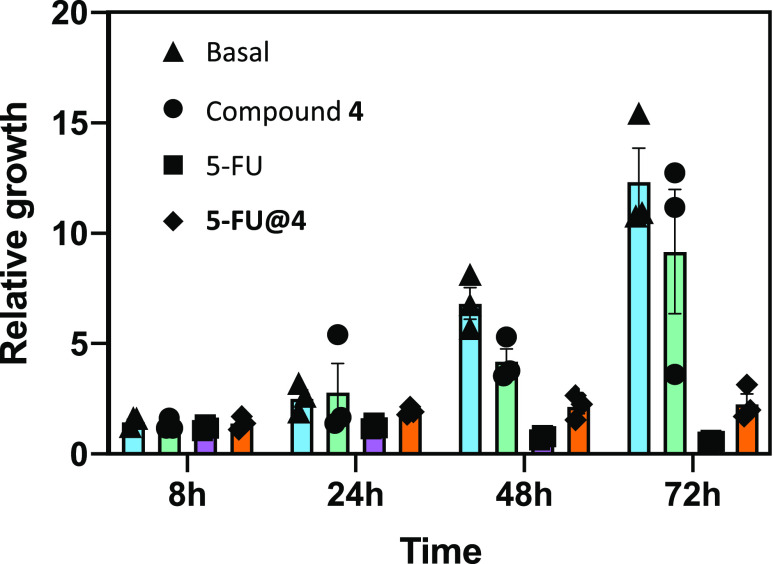
Relative growth of the HCT116 cells measured by crystal violet at different time points. Cells were grown at basal conditions or after the different compounds were added. Data represents the mean and standard error of three independent experiments. *p < 0.05; **p < 0.01 calculated by one tail, paired Students *t* test.

The 5-FU drug is widely employed in the treatment of several types of cancer. Prior to these studies, the **5-FU@4** sample required to be purified from any remaining particles of the highly insoluble 5-FU. The room temperature paramagnetism of the SMOF enables to purify the sample by using the magnetic sustentation phenomenon to retain the **5-FU@4** particles and get rid of the 5-FU excess present as diamagnetic particles. The cytotoxicity tests were performed on compounds **4**, 5-FU, and **5-FU@4**. The test performed using neat 5-FU implied the same amount of drug present in **5-FU@4**; likewise, the same amount of SMOF was used in the test performed with compound **4** and **5-FU@4**. These *in vitro* experiments on a human intestinal cell line (HCT116) are aimed to provide a preliminary insight into the biological activity of the porous matrix and also on its performance as a drug delivery system. The colon cell line HCT116 has been incubated for 72 h with the following concentrations: compound **4** 2 μg/100 μL; **5-FU@4** 2.56 μg/100 μL; and 5-FU 0.56 μg/100 μL for each of the components to be in the same concentration and obtaining comparative results. As observed in Figures 13 and S33, compound **4** is able by itself to provide a certain delay in cell growth, 5-FU alone decimates the cell culture from the very beginning, and **5-FU@4** provides an intermediate behavior that has been attributed to the delayed release of the cytotoxic 5-FU drug. As a result, we can conclude that the **5-FU@4** sample is able to produce a cytotoxic effect on the cell culture while delivering the active drug molecule more slowly. The latter is a crucial factor when considering the small range that exists between the therapeutic and toxic/lethal dosage for this drug.^55^

## Conclusions

This work has provided great insight into the possibilities arising from these supramolecularly assembled metal–organic porous materials (SMOFs). They not only provide very insoluble and water-stable materials but also incorporate very interesting features that come from the more flexible nature of the supramolecular interactions (especially when dealing with π-stacking interactions). These supramolecular interactions can also provide a high degree of predictability, which allows employing an isoreticular design approach (so fruitful for MOFs) while retaining the previously explained flexibility. In fact, the designed and synthesized isoreticular family of SMOFs provides tailorable pore sizes and porosity degrees based on the length of the employed organic dicarboxylate anions that hold together the [Cu_7_(μ-adeninato-κ*N3*:κ*N9*)_6_(μ_3_-OH)_6_(μ-OH_2_)_6_]^2+^ heptameric units through a predictable π-stacking and hydrogen bonding combination.

Furthermore, the compounds show a breathing effect with respect to the amount of water placed inside the pores of the supramolecular structure, being able to distinguish between open and closed forms. This process is reversible upon exposure to a humid atmosphere, involving a great volume expansion. This phenomenon endows the materials with a stimuli-response behavior that has enabled the generation of a humidity sensor based on changes of the electrical resistance taking place upon different humidity levels.

On the other hand, their stability in water and flexible behavior allow them to capture and release different adsorbate molecules in aqueous solutions, among which are several drug molecules. The uptake and release of these drug molecules can be monitored through the paramagnetic response of these materials toward an external magnetic field. Finally, one of these SMOFs loaded with the antitumoral 5-FU was tested in cytotoxic assays in the HCT116 cell culture, showing its potential as a drug carrier. All in all, the above diverse properties and applications must be understood as the starting point for these compounds to be considered multifunctional materials.

## Supporting Information Available

Details on the synthesis; FTIR spectroscopic data; thermogravimetric analysis; X-ray powder diffraction data; crystallographic and structural data; magnetic analysis; water adsorption isotherms; SEM images; adsorptive molecule size and shape analysis; adsorption data quantification by ^1^H-NMR spectroscopy and magnetic sustentation technique (PDF)
