# Supplementary material for: Isoreticular Chemistry and Applications of Supramolecularly Assembled Copper–Adenine Porous Materials
Source: Inorg Chem. 2023 Nov 1;62(45):18496–509. doi: 10.1021/acs.inorgchem.3c02708 (PMC10647167; doi:10.1021/acs.inorgchem.3c02708)
Supplement: Supplementary file 1 — ic3c02708_si_001.pdf [file ic3c02708_si_001.pdf]

Supporting Information for

Isoreticular Chemistry and Applications of Supramolecularly Assembled Copper–Adenine Porous Materials

Sandra Mena-Gutiérrez,[a,†] *Jon Pascual-Colino*[a,b,†]*, Garikoitz Beobide,*[a,b] *Oscar Castillo,*[a,b,*] *Ainara Castellanos-Rubio,*[c,d,e] *Antonio Luque,*[a,b] *Ekain Maiza-Razkin,*[a] *Jon Mentxaka,*[e,f] *Sonia Pérez-Yáñez*[a,b]

Corresponding autor e-mail: oscar.castillo@ehu.eus

[a]Departamento de Química Orgánica e Inorgánica, Facultad de Ciencia y Tecnología, Universidad del País Vasco/Euskal Herriko Unibertsitatea, UPV/EHU, Apartado 644, E-48080 Bilbao, Spain.

[b]BCMaterials, Basque Center for Materials, Applications and Nanostructures, UPV/EHU Science Park, E-48940 Leioa, Spain.

[c]Departamento de Genética, Antropología física y Fisiología animal, Facultad de Medicina, Universidad del País Vasco/Euskal Herriko Unibertsitatea, UPV/EHU, E-48940 Leioa, Spain.

[d]Ikerbasque, Basque Foundation for Science; E-48011, Bilbao, Spain.

[e]Biobizkaia Research Institute, E-480903 Barakaldo, Bizkaia, Spain.

[f]Departamento de Bioquímica y Biología Molecular, UPV-EHU, E-48940 Leioa, Bizkaia, Spain.

[†]These authors (S.M.G. and J.P.C.) contributed equally, they performed the experiments and prepared the manuscript and the supplementary information.

S1. SYNTHESIS OF THE COMPOUNDS 3
S2. FOURIER TRANSFORM INFRARED SPECTRA (FTIR) 5
S3. THERMOGRAVIMETRIC ANALYSIS 7
S4. POWDER X-RAY DIFFRACTION 9
S5. CRYSTAL STRUCTURE OF COMPOUNDS 11
S6. MAGNETIC ANALYSIS 28
S7. WATER ADSORPTION AND HUMIDITY SENSOR 32
S8. MAGNETIC SUSTENTATION METHOD 34
S9. SORPTION DATA QUANTIFICATION BY PROTON NUCLEAR MAGNETIC RESONANCE (^{1}H-NMR) 35
S10. POWDER X-RAY DIFFRACTION PATTERNS AND FTIR SPECTRA OF THE DRUG LOADED COMPOUND 4 39
S11. ADSORPTIVE VOLUMEN AND SHAPE 41
S12. CYTOTOXICITY ASSAYS 44

S1. SYNTHESIS OF THE COMPOUNDS

Crystallization procedure

All the compounds were crystallized and retrieved following the method explained in Figure S1 and S2:

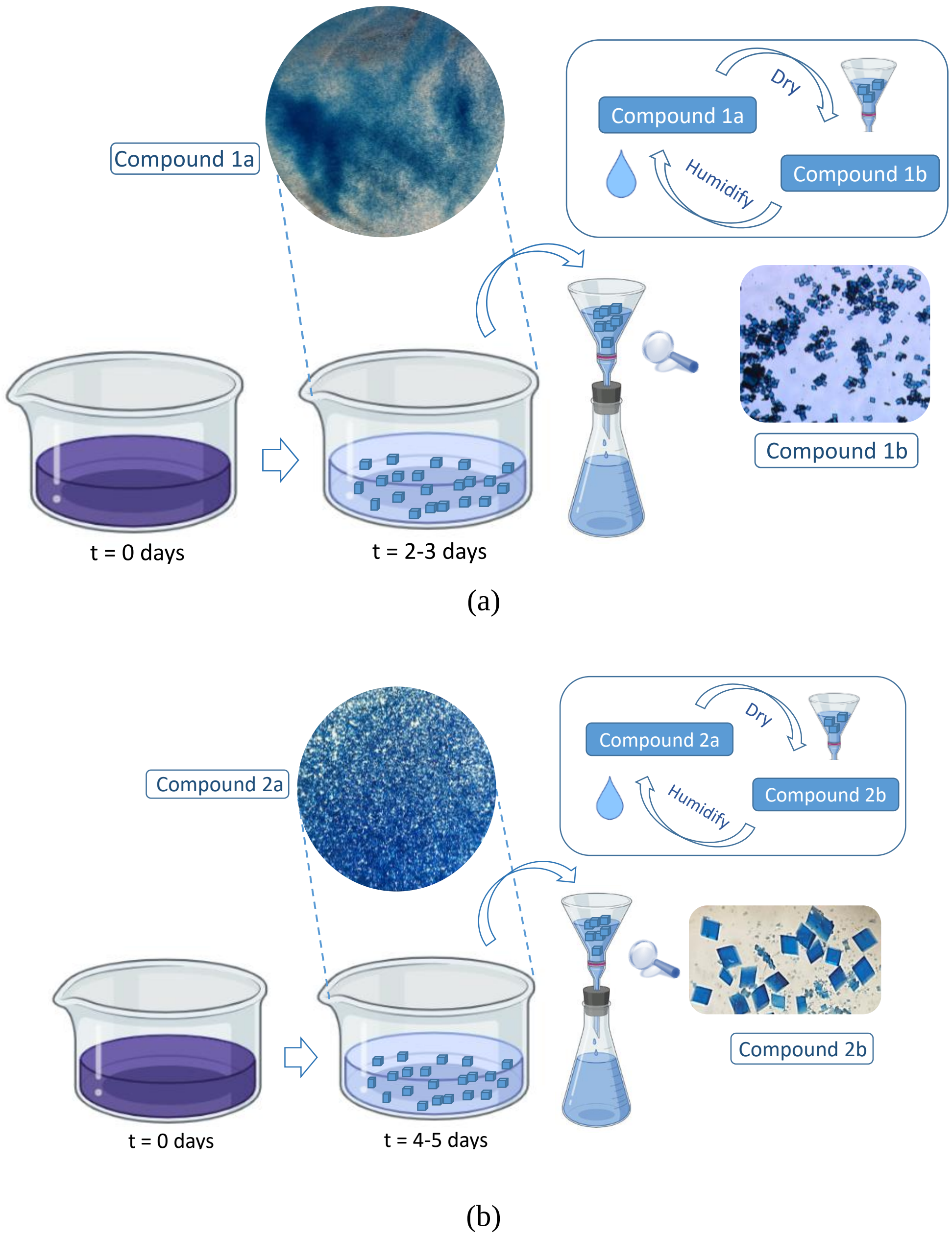

Figure S1. Synthesis process of compounds containing fumarate (**1a**, **1b**) and acetylenedicarboxylate (**2a**, **2b**) dianions, respectively.

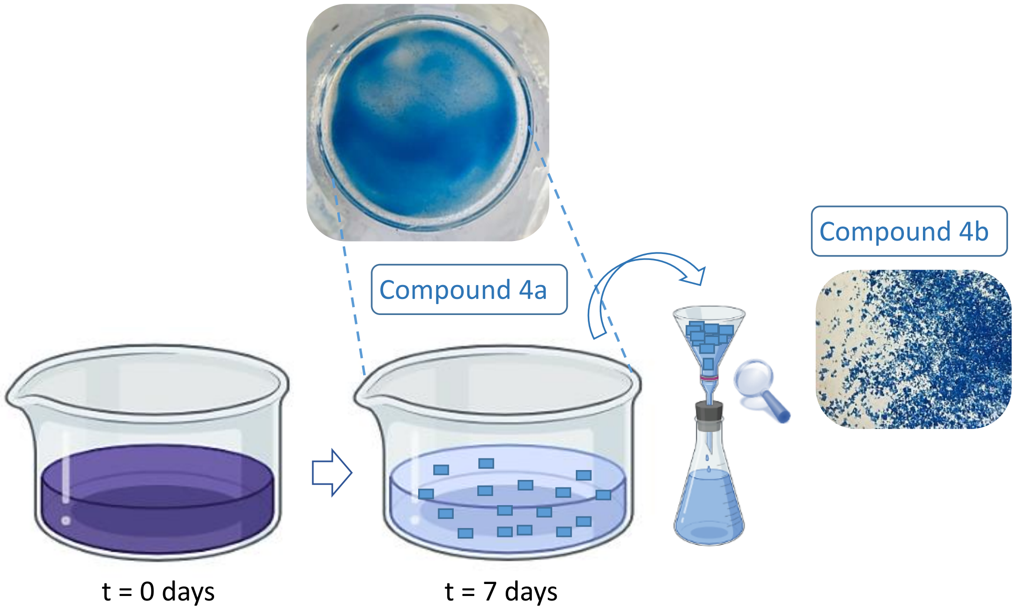

(a)

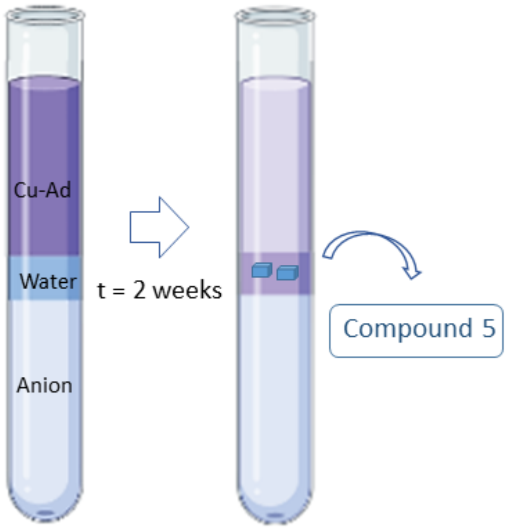

(b)

Figure S2. Synthesis process of compounds containing: (a) naphthalene-2,6-dicarboxylate (**4a**, **4b**) and (b) biphenyl-4,4´-dicarboxylate dianion (**5**), respectively.

Table S1. C and N elemental analysis of compounds **1b**, **2b** and **4b**.

	Calculated			Experimental		
**Compound**	**C (%)**	**N (%)**	**CN (ratio)**	**C (%)**	**N (%)**	**CN (ratio)**
**1b**	21.93	22.57	0.97	22.23	22.64	0.98
**2b**	21.74	22.38	0.97	21.66	22.31	0.97
**4b**	23.35	19.45	1.20	22.49	18.60	1.21

S2. FOURIER TRANSFORM INFRARED SPECTRA (FTIR)

All FTIR spectra exhibit the bands corresponding to the stretching vibration of the C–H and N–H bonds of the purine bases between 3400 and 3100 cm^{-1}, together with the O–H bonds of the water molecules. The bands between 1700 and 1600 cm^{-1} belong to the nucleobase ligands for the imino groups. The antisymmetric tension of the carboxylate group of anions are observed around 1640 cm^{-1}. The presence of the peak located around 1600 cm^{-1} corresponding to the vibration of the C=C bond and the deformation of the NH_2 group allows the identification of adeninato ligands. The vibrational bands of the M–N bonds appear below 550 cm^{-1}.

Table S2. FTIR assignation.

**Adenine**	**HFum** [a]	**HAcet**	**HNaph**	**1b**	**2b**	**4b**	**Assignation**[b, c]
				3450vs	3420vs	3440vs	υ O─H,
3296s				3350vs	3350w	3340w	υ N─H
3123s	3088m		3100w	3210s		3190w	υ C─H
		2232m			2130m	─	υ C≡C
1670vs	1699vs	1680vs	1694vs	1640vs	1640vs	1640vs	$υ_{as}$ C=O + δ NH_2
1600vs	1620s	1620m	1605m	1600m	1600m	1600m	υ C=C+ υ C=N
	1540w	1520m	1570w	1550s	1550s	1540s	$υ_s$ C=O
1504s				1500w	1500m	1490m	υ C─NH_2
	1496vs	1440s	1504s	1460vs	1460vs	1460vs	$υ_{as}$ COO
1420vs	1406m		1420vs	1400vs		1400vs	$δ_{ring +}$ δ C─H
	1382vs	1375s	1344vs	1370s	1340s	1340s	$υ_s$ COO
	1296s<br>1152s	1283s<br>1120m	1290vs<br>1140s	1280s<br>1140vs	1280m<br>1150s	1270m<br>1140s	$υ_s$ C─O
1230ss	1202s	1200s	1993s	1190vs	1200s	1200s	$δ_{ip}$ CCH
1020vs	1050s	1060w	1090m	1040m	1030w	1040m	γ C─H +γ NH_2 + υ C─C_{arom}
930vs	933m	930w	920m	930w	940w	930w	δ C─H, δ C─C
790vs	782m	820m	780m	800m	780m	790m	$δ_{ip}$ C─H, ω NH_2
720s<br>640vs	740m<br>660s	750vs<br>650vs	750vs<br>630m	740m<br>670s	740m<br>660m	740m<br>650m	$δ_{ip}$ ring defor., $δ_{oop}$ COO^-
				520vs<br>470m	550m<br>450m	550m<br>450m	υ (M-N)

[a] HFum: fumaric acid, HAcet: acetylenedicarboxyilic acid, HNaph: naphthalene-2,6-dicarboxylic acid. [b]vs: very strong, s: strong, m: medium, w: weak. [c]s: symmetric, as: antisymmetric, υ: stretching vibration, δ: bending vibration, γ:rocking, ω: wagging, ip = in plane, oop = out of plane. [Brese, N.E.; O'Keeffe, M. *Acta Cryst.* **1991**, *B47*, 192-197. Brown, I.D.. *The Chemical Bond in Inorganic Chemistry: The Bond Valence Model*. Oxford University Press, Oxford, 2002. Mohamed, T.A., Shabaan, I.A.; Zoghaid, W.M.; Husband, J.; Farag, R.S.; Alajhaz, A.E. **2009**, *J. Mol. Struct.* , *938*, 263-276, Kanagathara, N.; Pawlus, K.; Marchewka, M.K. **2018**, *Acta Phys. Pol. A*, *133*, 45-56.]

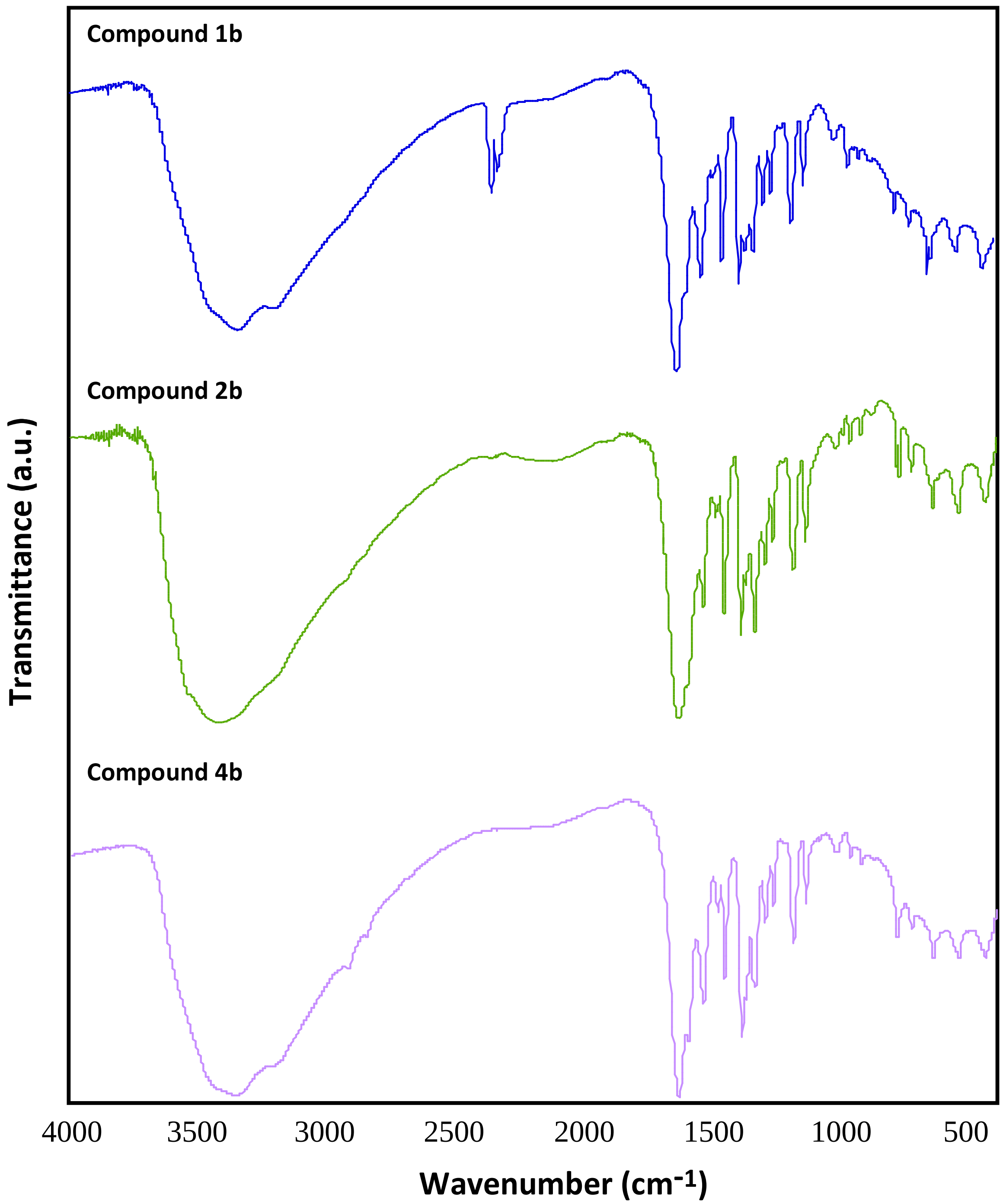

Figure S3. FTIR spectra of compounds **1b**, **2b** and **4b**.

S3. THERMOGRAVIMETRIC ANALYSIS

Thermogravimetric analysis of compounds (**1b**, **2b**, **4b**) were performed in synthetic air (79% N_2, 21% O_2) from 30 °C to 600 °C with a temperature increase rate of 5 °C/min. All experimental mass losses fit fairly well with those expected from the chemical formula of the compound. The thermograms show four main weight loss stages. Crystallization solvent water molecules are released at a temperature range of 25 – 100 °C. Thereafter, the compounds lose the six coordination water molecules at 75–140 °C range. This mass loss stage is overlapped with the loss of three more water molecules due to the dehydroxilation of six hydroxide group of the cluster at a range of 140 – 195 °C. After that, the desamination stage occurred at the temperature range of 180 – 290 °C. Finally, the framework decomposition takes place to lead CuO (PDF: 48-1548) as final residue above 450 °C.

Table S3. Thermoanalytic data for compounds **1b**, **2b** and **4b**.[a]

**Step**	**T_i**	**T_{peak}**	**T_f**	**Δm(%)**	**ΣΔm(%)**	**ΣΔm(%)$_{theor}$**
**Comp. 1b**						
**1**	25	125	140	21.82	21.82	21.27 (–16 H_2O + – 6 H_2O)
**2**	140		190	3.12	24.94	24.17 (–3 H_2O)
**3**	200	220	240	5.19	30.13	29.32 (–6 NH_2)
**4**	330	440	600	40.29	70.42	70.10 (CuO)
**Comp. 2b**						
**1**	25	50	100	17.14	17.14	16.29 (–17 H_2O)
**2**	100	135	195	8.58	25.72	24.92 (–9 H_2O)
**3**	200	225	240	4.93	30.65	30.03 (–6 NH_2)
**4**	250	435	600	39.90	70.55	70.35 (CuO)
**Comp. 4b**						
**1**	25	75	125	27.54	27.54	27.52 (– 27 H_2O + – 6 H_2O)
**2**	125		180	2.59	30.13	30.02 (–3 H_2O)
**3**	180	230	290	4.17	34.30	34.46 (–6 NH_2)
**4**	300	380	600	39.87	74.17	74.22 (CuO)

[a] T_i = initial temperature; T_{peak} = DTA peak temperature; T_f = final temperature; Δm(%) = mass loss percentage for each process; ΣΔm(%) = total mass loss percentage; ΣΔm(%)$_{theor}$ = theoretical total mass loss percentage.

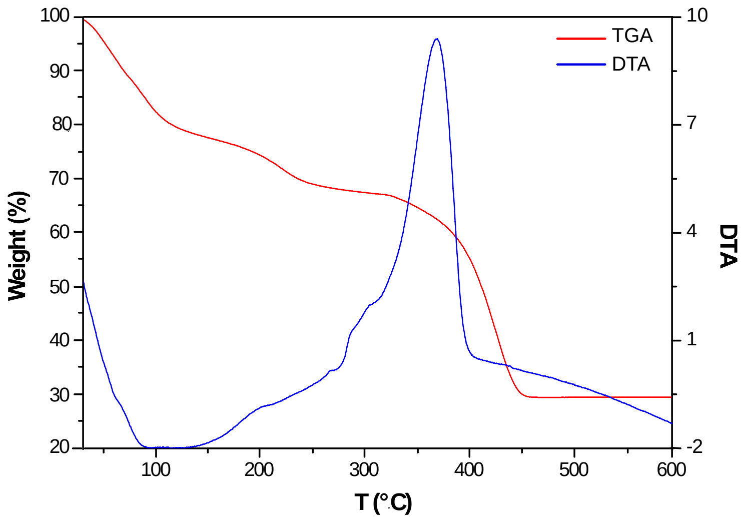

(a)

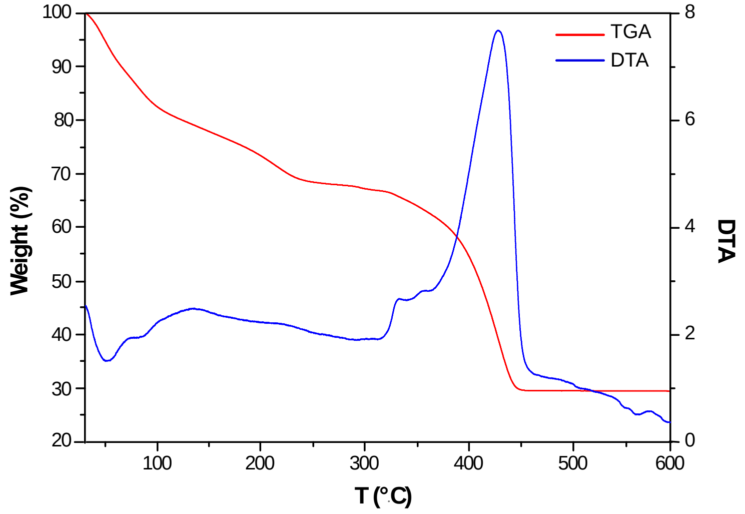

(b)

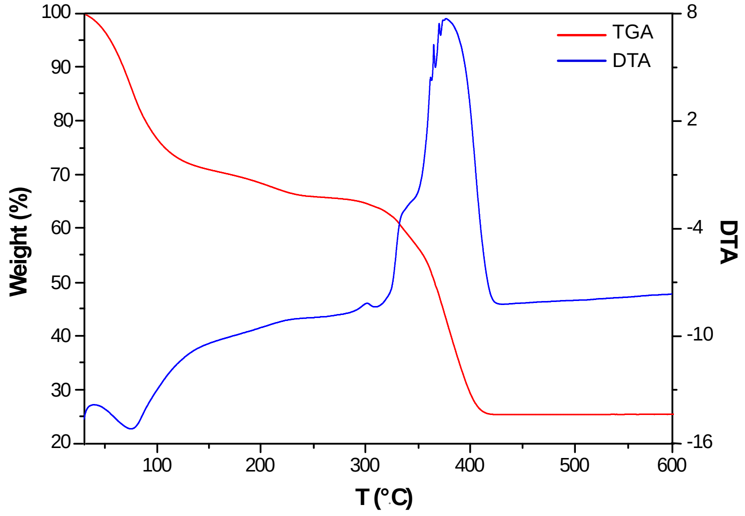

(c)

Figure S4. Thermogravimetric data (TGA-DTA curves) for compounds **1b** (a), **2b** (b) and **4b** (c).

S4. POWDER X-RAY DIFFRACTION

Thermodiffraction experiments were carried out by heating samples of the different compounds, at intervals of 10 °C, to analyze the thermal effect on the structural stability of the compounds. In the following lines a general description, which is suitable for all compounds, is provided. At temperatures higher than 100 °C, where the thermal analysis showed that the crystallization water molecules are lost, the crystallinity of the sample decreases and the displacement of the diffraction peaks to higher angles indicates the contraction of the volume of the unit cell. At 150 °C the further removal of coordination water molecules leads to a significant loss of crystallinity. The change in crystallinity after dehydration was confirmed by activating a sample by heating it at 30 °C, under vacuum, for eight hours. The diffractogram of the activated sample indicates that a high loss of crystallinity occurs. When the activated sample is stored in a humidifier with a degree of humidity of *ca*. 90%, after 24 hours, its weight increases, it recovers crystallinity and its diffractogram is coincident with that of the initial hydrated compound.

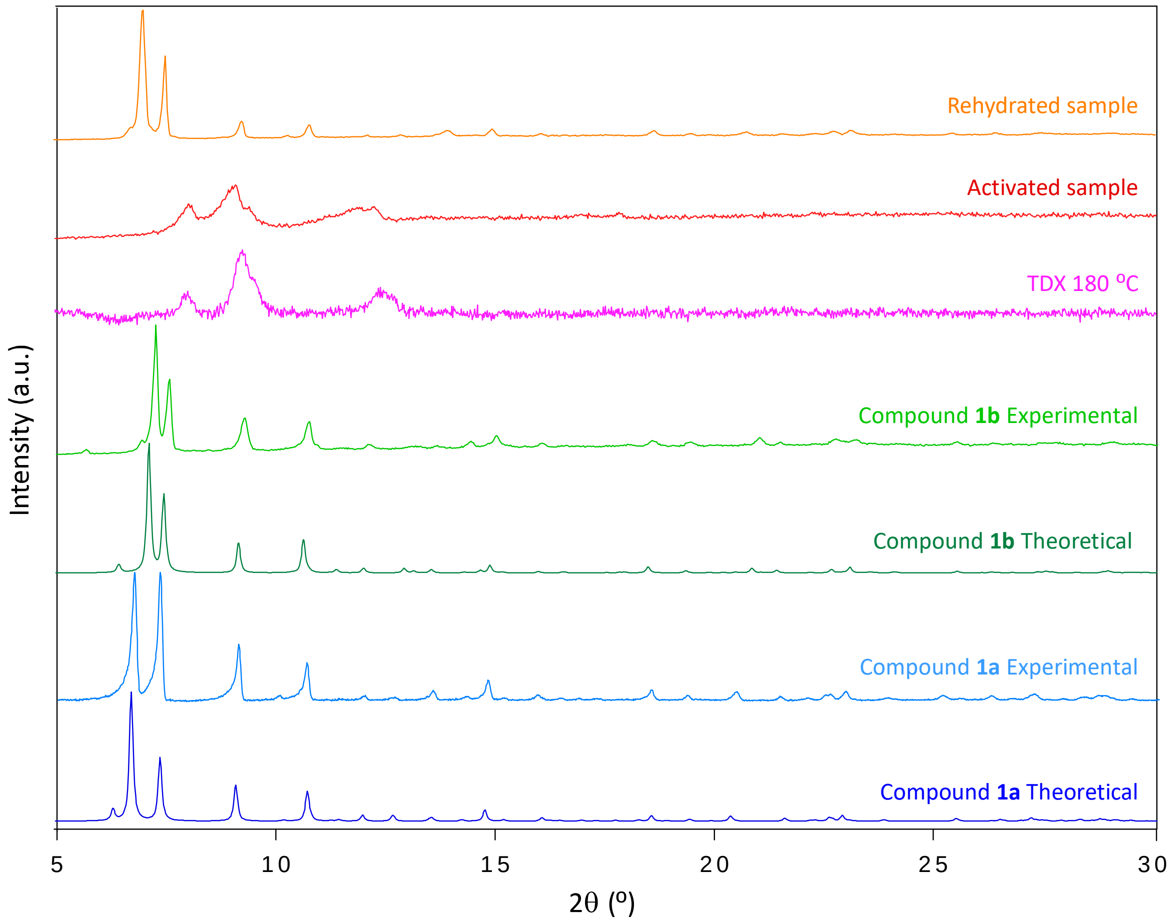

Figure S5. Diffractograms of samples with the fumarate anion (**1**).

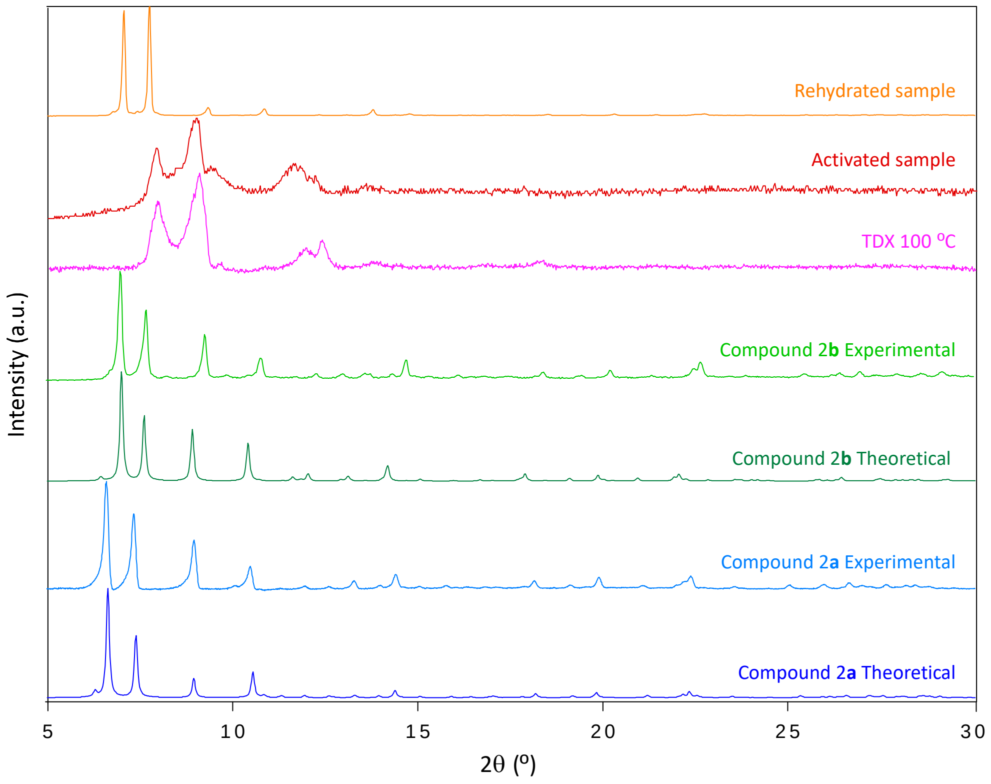

(a)

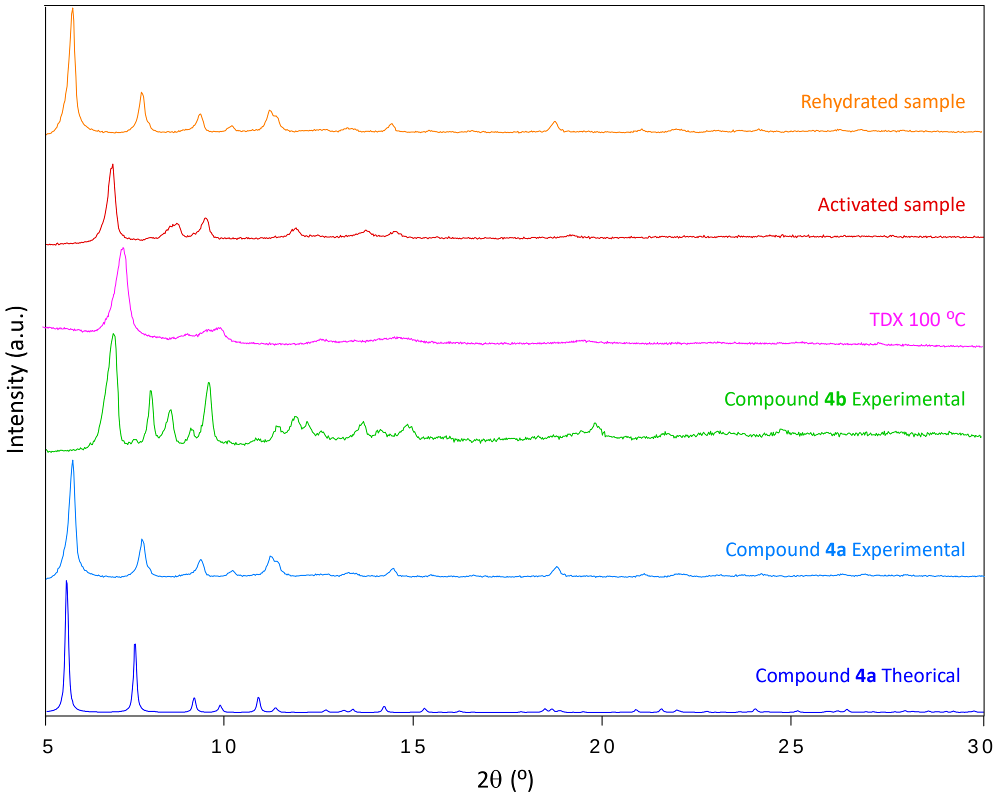

(b)

Figure S6. Diffractograms of samples with the (a) acetylenedicarboxylate (**2**) and (b) naphthalene-2,6-dicarboxylate anion (**4**).

S5. CRYSTAL STRUCTURE OF COMPOUNDS

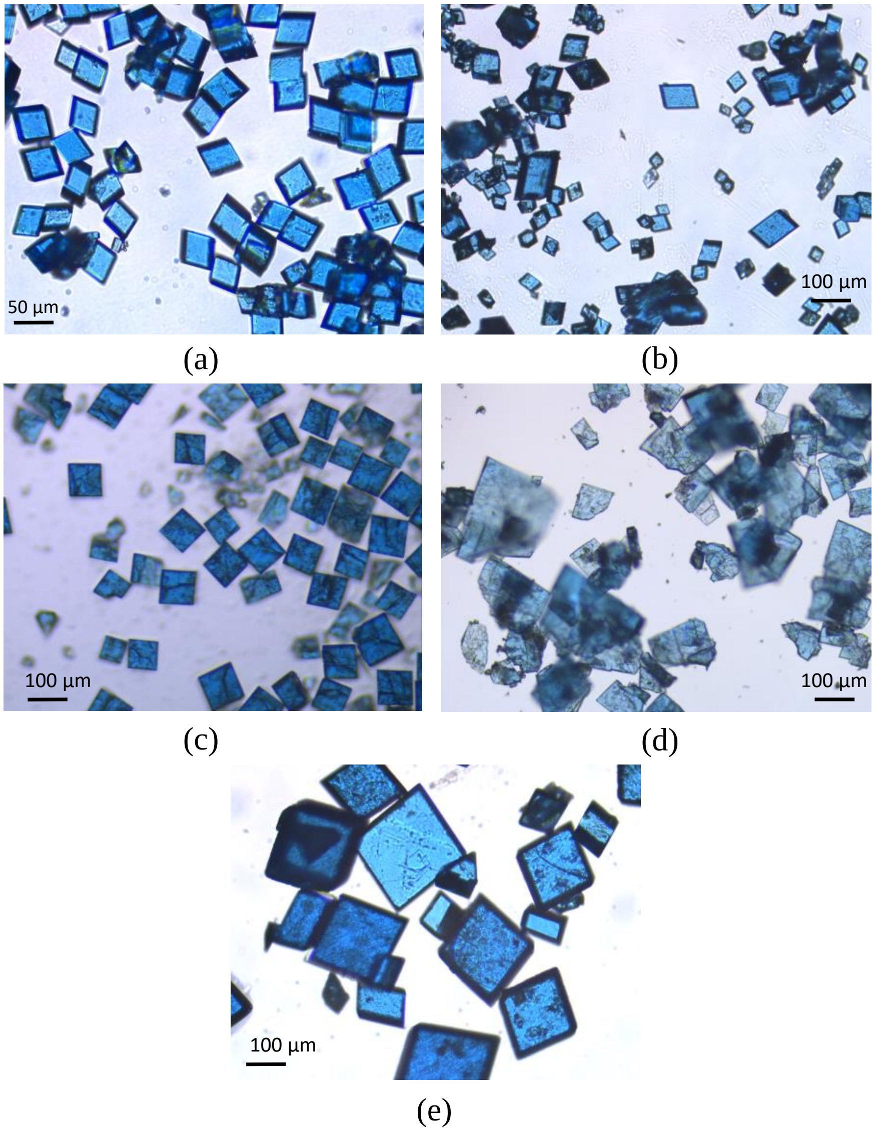

Figure S7. Optical images of crystals corresponding to: (a) **1b**, (b) **2b**, (c) **3**, (d) **4b**, (e) **5**.

All non-hydrogen atoms were refined anisotropically, except those corresponding to disordered atoms. The hydrogen atoms belonging to organic entities have been geometrically fixed and refined according to a riding model with an isotropic thermal parameter linked to the atom to which they are attached (120 %). In most of the cases, the hydrogen atoms of the ligands and anions have been located in the difference Fourier map, while for the coordination water molecules cases the routine CALC-OH (Nardelli M. *J. Appl. Crystallogr*. **1999**, *32*, 563-571). implemented in WINGX (Farrugia, L. J. *J. Appl. Crystallogr*. **2012**, 45, 849–854) interface has been employed. The refinement of water hydrogen atoms has been performed using an isotropic thermal parameter of 150% regarding their parent atom. It has not been possible positioning

geometrically the hydrogen atoms of all crystallization water molecules due to the disorder that many of them present. During the structural solution of the compounds, it was common to observe the presence of a static disorder in the adenine molecules with an unusually high thermal motion of some atoms and nearby peaks in the Fourier map differences with high electron density was also observed. This disorder corresponds to the existence of two coplanar positions of the nucleobase with inverted orientation with respect to the coordination mode (μ-κ*N*3:κ*N*9/μ-κ*N*9:κ*N*3). The disorder was modelled including the peaks observed as atoms split in two positions (A and B), to which common free occupancy factors were assigned for each of the subgroups with the condition that the two occupancy factors add up to 100%.

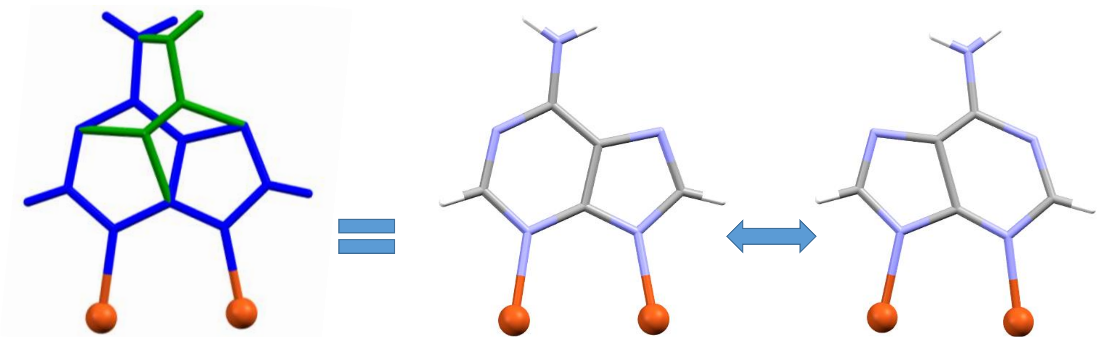

Figure S8. Disorder of the adeninato ligand with two coplanar orientations colored in blue and green.

The crystal structures of all these compounds show the presence of great channels in which the solvent molecules are placed highly disordered. It precluded their modeling and, therefore, the electron density at the voids of the crystal structure was subtracted from the reflection data by the SQUEEZE method as implemented in PLATON. Once the process has been carried out, it is verified that the holes generated by the program are suitable for the presence of those amounts of water molecules, assigning the value of ten electrons for each water molecule. The representation of the crystal structures has been made by MERCURY (MacRae, C. F.; Sovago, I.; Cottrell, S. J.; Galek, P. T. A.; McCabe, P.; Pidcock, E.; Platings, M.; Shields, G. P.; Stevens, J. S.; Towler, M.; Wood, P. A. *J. Appl. Crystallogr.* **2020**, *53*, 226–235.).

Table S4. Crystallographic data and structure refinement details of compounds **1-5**.[a,b]

	**1a**	**1b**	**2a**	**2b**	**4a**	**5**
empirical formula	$C_{34}H_{88}Cu_7N_{30}O_{38}$	$C_{34}H_{76}Cu_7N_{30}O_{32}$	$C_{34}H_{86}Cu_7N_{30}O_{38}$	$C_{34}H_{76}Cu_7N_{30}O_{33}$	$C_{42}H_{112}Cu_7N_{30}O_{48}$	$C_{44}H_{138}Cu_7N_{30}O_{60}$
formula weight	1970.12	1862.02	1968.10	1878.02	2250.33	2492.62
crystal system	Monoclinic	Monoclinic	Monoclinic	Monoclinic	Monoclinic	Monoclinic
space group	*C*2/*c* (15)	*C*2/*c* (15)	*C*2/*c* (15)	*C*2/*c* (15)	$P2_1/c$ (14)	*I*2/*c* (15)
a	28.462(5)	35.805(7)	28.4832(5)	26.3048(16)	15.9866(12)	18.2720(6)
b	16.489(1)	16.417(1)	16.4538(2)	16.2582(7)	15.8405(7)	15.9398(5)
c	17.881(2)	17.877(3)	17.9449(3)	17.7037(12)	18.4074(9)	37.2369(16)
α	90	90	90	90	90	90
β	113.016(11)	136.27(4)	111.355(2)	107.089(7)	95.424(5)	93.503(3)
γ	90	90	90	90	90	90
V ($Å^3$)	7723.6(18)	7265(4)	7832.6(2)	7237.0(8)	4640.5(5)	10825.1(7)
Z	4	4	4	4	2	4
T (K)	150.0(1)	170.0(1)	150.1(3)	150.1(1)	151.0(2)	100.0(1)
λ (Å)	0.71073	0.71073	1.54184	0.71073	1.54184	0.71073
Size (mm)	0.08/0.06/0.04	0.03/0.02/0.02	0.10/0.10/0.05	0.13/0.12/0.11	0.07/0.07/0.02	0.08/0.07/0.03
Shape	Cubic	Cubic	Cubic	Cubic	Cubic/Square	Cube
Color	Blue	Blue	Blue	Blue	Blue	Blue
Max. and medium Δ/σ	0.000 / 0.000	0.000 / 0.000	0.000 / 0.000	0.001 / 0.000	0.000 / 0.000	0.000 / 0.000
θ interval	1.649 – 28.269	2.114 – 29.960	4.496 – 73.126	2.407 – 28.729	3.688 – 72.697	1.983 – 23.999
hkl interval	$-33\le h\le 21$<br>$-19\le k\le 19$<br>$-17\le l\le 21$	$-48\le h\le 44$<br>$-22\le k\le 22$<br>$-25\le l\le 24$	$-35\le h\le 34$<br>$-20\le k\le 18$<br>$-20\le l\le 22$	$-25\le h\le 34$<br>$-21\le k\le 21$<br>$-22\le l\le 23$	$-18\le h\le 19$<br>$-18\le k\le 19$<br>$-14\le l\le 22$	$-20\le h\le 20$<br>$-18\le k\le 16$<br>$-42\le l\le 42$
ρ_c ($g\cdot cm^{-3}$)	1.694	1.702	1.669	1.724	1.611	1.529
μ (cm^{-1})	1.995	2.110	2.969	2.120	2.669	1.456
F(000)	4036	3796	4028	3828	2322	5180
S^a	0.957	1.249	1.050	0.921	1.003	0.977
R_{int}	0.1702	0.0668	0.0317	0.0786	0.1037	0.0628
Parameters	400	410	417	286	444	471
Weight scheme[c]	Shelx	Shelx	Shelx	Shelx	Shelx	Shelx
final R indices						
[I > 2σ(I)] $R_1{}^b/wR_2{}^c$	0.1043/0.2863	0.1678/0.4568	0.0832/0.2408	0.1397/0.3863	0.1166/0.3157	0.0630/0.1806
all data $R_1{}^b/wR_2{}^c$	0.2336/0.3764	0.3247/0.5238	0.0929/0.2541	0.2977/0.4355	0.2030/0.3700	0.1021/0.1931

[a]$S=[\sum w(F_0^2-F_c^2)^2/(N_{obs}-N_{param})]^{1/2}$. [b]$R_1=\sum||F_0|-|F_c||/\sum|F_0|$. [c]$wR_2 = [\sum w(F_0^2-F_c^2)^2/\sum wF_0^2]^{1/2}$; $w = 1/[\sigma^2(F_0^2) + (aP)^2 + b]$ where $P = (\max(F_0^2,0) + 2F_c^2)/3$; **1a** (a = 0.1718, b = 0); **1b** (a = 0.2000, b = 0), **2a** (a = 0.1351, b = 38.8421), **2b** (a = 0.2000, b = 0) **4a** (a = 0.2000, b = 0) and **5** (a = 0.1090, b = 0).

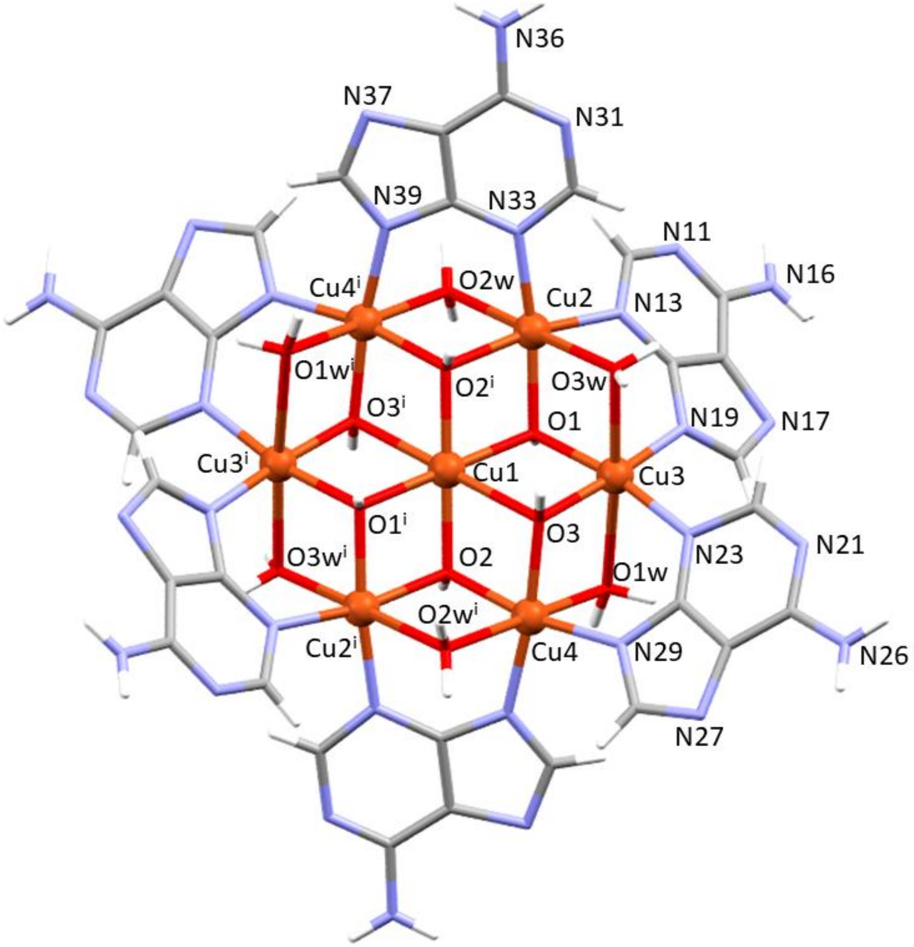

(a)

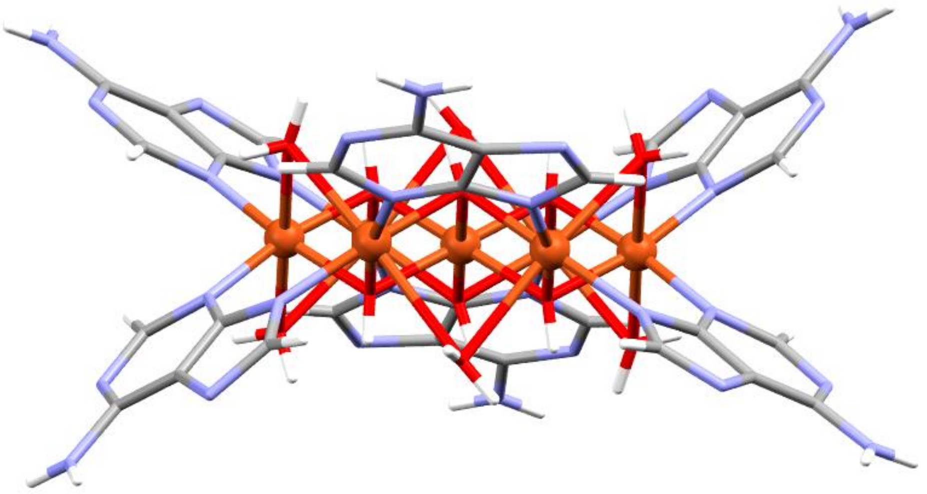

(b)

Figure S9. Heptameric copper cluster showing the numbering scheme. For the sake of clarity, only the adeninato ligands of the asymmetric unity are labeled.

Table S5. Distances and angles (Å, deg) for compound **1a**.[a]

Cu1–O1	2.079(9)	Cu1–O2	2.095(8)	Cu1–O3	2.001(8)
Cu2–O1	1.976(9)	Cu2–O3	2.017(9)	Cu2–N13	1.997(1)
Cu2–N39^{i}	2.011(1)	Cu2–O1w	2.508(1)	Cu2–O3w^{i}	2.497(1)
Cu3–O1	1.973(8)	Cu3–O2	1.957(9)	Cu3–N19	1.966(2)
Cu3–N29	1.961(2)	Cu3–O1w	2.466(1)	Cu3–O2w	2.475(1)
Cu4– O2	1.961(9)	Cu4–O3^{i}	1.995(8)	Cu4–N23	2.000(1)
Cu4–N33	2.026(1)	Cu4–O2w	2.339(1)	Cu4–O3w	2.500(1)
Cu1···Cu2	3.073(2)	Cu1···Cu3	3.149(2)	Cu1···Cu4	3.093(2)
Cu2···Cu3	3.101(3)	Cu2···Cu4^{i}	3.142(3)	Cu3···Cu4	3.073(3)
Cu1–O1–Cu2	98.5(4)	Cu1–O1–Cu3	102.0(4)	Cu2–O1–Cu3	103.5(4)
Cu1–O2–Cu3	101.9(4)	Cu1–O2–Cu4	99.3(4)	Cu3–O2–Cu4	103.3(4)
Cu1–O3–Cu2	99.8(4)	Cu1–O3–Cu4i	101.4(4)	Cu2 –O3–Cu4^{i}	103.1(4)
Cu2 –O1w–Cu3	77.1(3)	Cu3–O2w–Cu4	79.3(3)	Cu3–O3w–Cu2^{i}	77.9(4)

[a]Symmetry: (i) – 1/2 – x, 1/2 – y, – z.

Table S6. Distances and angles (Å, deg) for compound **1b**.[a]

Cu1–O1	1.961(1)	Cu1–O2	2.210(1)	Cu1–O3	2.108(1)
Cu2–O1	1.997(1)	Cu2–O3	1.913(1)	Cu2–N13	2.020(2)
Cu2–N39^{i}	2.040(3)	Cu2–O1w	2.469(2)	Cu2–O3w^{i}	2.540(3)
Cu3–O1	1.965(1)	Cu3–O2	1.962(1)	Cu3–N19	2.150(2)
Cu3–N29	1.860(2)	Cu3–O1w	2.500(2)	Cu3–O2w	2.590(2)
Cu4–O2	1.972(2)	Cu4–O3^{i}	2.012(1)	Cu4–N23	2.080(2)
Cu4–N33	1.970(2)	Cu4–O2w	2.410(3)	Cu4–O3w	2.510(3)
Cu1···Cu2	3.067(2)	Cu1···Cu3	3.093(3)	Cu1···Cu4	3.132(2)
Cu2···Cu3	3.107(4)	Cu2···Cu4^{i}	3.109(4)	Cu3···Cu4	3.078(4)
Cu1–O1–Cu2	101.6(8)	Cu1–O1–Cu3	104(8)	Cu2–O1–Cu3	103.3(7)
Cu1–O2–Cu3	98.5(7)	Cu1–O2–Cu4	99.8(7)	Cu3–O2–Cu4	103.0(8)
Cu1–O3–Cu2	99.3(8)	Cu1–O3–Cu4^{i}	98.9(6)	Cu2–O3–Cu4^{i}	104.7(8)
Cu2–O1w–Cu3	77.4(6)	Cu3–O2w–Cu4	76.0(8)	Cu4–O3w–Cu2^{i}	75.9(8)

[a]Symmetry: (i) 1/2 – x, 1/2 – y, 1 – z.

Table S7. Distances and angles (Å, deg) for compound **2a.**[a]

Cu1–O1	2.012(4)	Cu1–O2	2.125(3)	Cu1–O3	2.048(4)
Cu2–O1	1.991(4)	Cu2–O3^{i}	1.962(4)	Cu2–N13	1.982(7)
Cu2–N39^{i}	2.015(7)	Cu2–O1w	2.473(4)	Cu2–O3w	2.531(6)
Cu3–O1	1.989(3)	Cu3–O2	1.957(5)	Cu3–N19	1.970(6)
Cu3–N29	1.992(6)	Cu3–O1w	2.490(4)	Cu3–O2w	2.496(4)
Cu4–O2	1.960(4)	Cu4–O3	1.980(3)	Cu4–N23	2.016(6)
Cu4–N33	2.012(6)	Cu4–O2w	2.369(5)	Cu4–O3w^{i}	2.510(6)
Cu1···Cu2	3.079(9)	Cu1···Cu3	3.126(9)	Cu1···Cu4	3.110(8)
Cu2···Cu3	3.119(1)	Cu2···Cu4^{i}	3.123(1)	Cu3···Cu4	3.072(1)
Cu1–O1–Cu2	100.5(2)	Cu1–O1–Cu3	102.8(2)	Cu2–O1–Cu3	103.2(2)
Cu1–O2–Cu3	99.8(2)	Cu1–O2–Cu4	99.1(2)	Cu3–O2–Cu4	103.3(2)
Cu1–O3–Cu2^{i}	100.3(2)	Cu1–O3–Cu4	101.1(2)	Cu4–O3–Cu2^{i}	104.8(2)
Cu2–O1w–Cu3	77.9(1)	Cu3–O2w–Cu4	78.3(1)	Cu2–O3w–Cu4^{i}	76.6(2)

[a] Symmetry: (i) 1/2 – x, 1/2 – y, 1 –z.

Table S8. Distances and angles (Å, deg) for compound **2b.**[a]

Cu1–O1	2.004(1)	Cu1–O2	2.096(1)	Cu1–O3	2.084(8)
Cu2–O1	2.012(1)	Cu2–O3^{i}	1.990(1)	Cu2–N19	1.999(8)
Cu2–N39^{i}	1.994(7)	Cu2–O1w	2.502(1)	Cu2–O3w^{i}	2.583(1)
Cu3–O1	1.982(1)	Cu3–O2	1.934(1)	Cu3–N13	1.978(6)
Cu3–N29	2.092(1)	Cu3–O1w	2.477(1)	Cu3–O2w	2.530(1)
Cu4– O2	1.969(9)	Cu4–O3	1.949(9)	Cu4–N23	1.918(1)
Cu4–N33	1.963(5)	Cu4–O2w	2.570(1)	Cu4–O3w	2.291(1)
Cu1···Cu2	3.071(2)	Cu1···Cu3	3.096(2)	Cu2···Cu4	3.129(2)
Cu2···Cu3	3.142(3)	Cu2···Cu4^{i}	3.080(3)	Cu3···Cu4	3.075(3)
Cu1–O1–Cu2	99.8(5)	Cu1–O1–Cu3	102.0(5)	Cu2–O1–Cu3	103.8(5)
Cu1–O2–Cu3	100.4(4)	Cu1–O2–Cu4	100.6(4)	Cu3–O2–Cu4	104.0(5)
Cu1–O3–Cu2^{i}	97.8(4)	Cu1–O3–Cu4	101.7(4)	Cu4–O3–Cu2^{i}	102.8(4)
Cu2–O1w–Cu3	78.3(3)	Cu3–O2w–Cu4	74.2(4)	Cu4–O3w–Cu2^{i}	78.1(4)

[a] Symmetry: (i) 3/2 – x, 1/2 –y, – z.

Table S9. Distances and angles (Å, deg) for compound **4a.**[a]

Cu1–O1	1.997(8)	Cu1–O2	2.012(8)	Cu1–O3	2.243(7)
Cu2–O1	1.974(8)	Cu2–O3^{i}	1.959(8)	Cu2–N13	2.006(1)
Cu2–N33^{i}	1.968(9)	Cu2–O1w	2.367(8)	Cu2–O3w^{i}	2.627(1)
Cu3–O1	1.990(8)	Cu3–O2	1.997(7)	Cu3–N19	1.972(1)
Cu3–N29	2.010(1)	Cu3–O1w	2.416(7)	Cu3–O2w	2.583(8)
Cu4–O2	1.998(7)	Cu4–O3	1.972(8)	Cu4–N23	1.962(1)
Cu4–N39	1.988(9)	Cu4–O2w	2.412(8)	Cu4–O3w	2.582(1)
Cu1···Cu2	3.104(2)	Cu1···Cu3	3.062(2)	Cu1···Cu4	3.164(2)
Cu2···Cu3	3.116(3)	Cu2···Cu4^{i}	3.080(2)	Cu3···Cu4	3.136(2)
Cu1–O1–Cu2	102.8(3)	Cu1–O1–Cu3	100.4(4)	Cu2–O1–Cu3	103.6(4)
Cu1–O2–Cu3	99.6(3)	Cu1–O2–Cu4	104.2(3)	Cu3–O2–Cu4	103.4(3)
Cu1–O3–Cu2^{i}	95.0(3)	Cu1–O3–Cu4	97.1(3)	Cu4 –O3–Cu2^{i}	103.2(4)
Cu2–O1w–Cu3	81.3(2)	Cu3–O2w–Cu4	77.7(2)	Cu4–O3w–Cu2^{i}	72.5(3)

[a] Symmetry: (i) 2 – x, – y, 2 – z.

Table S10. Distances and angles (Å, deg) for compound **5.**[a]

Cu1–O1	2.226(4)	Cu1–O2	2.014(5)	Cu1–O3	1.984(4)
Cu2–O1	1.943(5)	Cu2–O2^{i}	1.968(4)	Cu2–N13	1.981(6)
Cu2–N33	2.051(7)	Cu2–O1w	2.366(4)	Cu2–O3w	2.632(5)
Cu3–O1	1.959(5)	Cu3–O3	1.986(4)	Cu3–N19	1.997(6)
Cu3–N23	1.989(6)	Cu3–O2w	2.387(5)	Cu3–O3w	2.559(5)
Cu4–O2	1.970(4)	Cu4–O3	1.984(5)	Cu4–N29	1.973(6)
Cu4–N39^{i}	2.031(7)	Cu4–O1w^{i}	2.406(4)	Cu4–O2w	2.563(4)
Cu1···Cu2	3.112(8)	Cu1···Cu3	3.147(8)	Cu1···Cu4	3.060(9)
Cu2···Cu3	3.0879(1)	Cu2···Cu4^{i}	3.098(1)	Cu3···Cu4	3.135(1)
Cu1–O1–Cu2	96.3(2)	Cu1–O1–Cu3	97.3(2)	Cu2–O1–Cu3	104.6(2)
Cu1–O2–Cu4	100.4(2)	Cu1–O2–Cu2^{i}	102.8(2)	Cu4–O2–Cu2^{i}	103.7(2)
Cu1–O3–Cu3	104.9(2)	Cu1–O3–Cu4	100.9(2)	Cu3–O3–Cu4	104.3(2)
Cu2–O1w–Cu4^{i}	80.9(1)	Cu3–O2w–Cu4	78.5(1)	Cu2–O3w–Cu3	72.9(1)

[a]Symmetry: (i) 2 – x, – y, 1 – z.

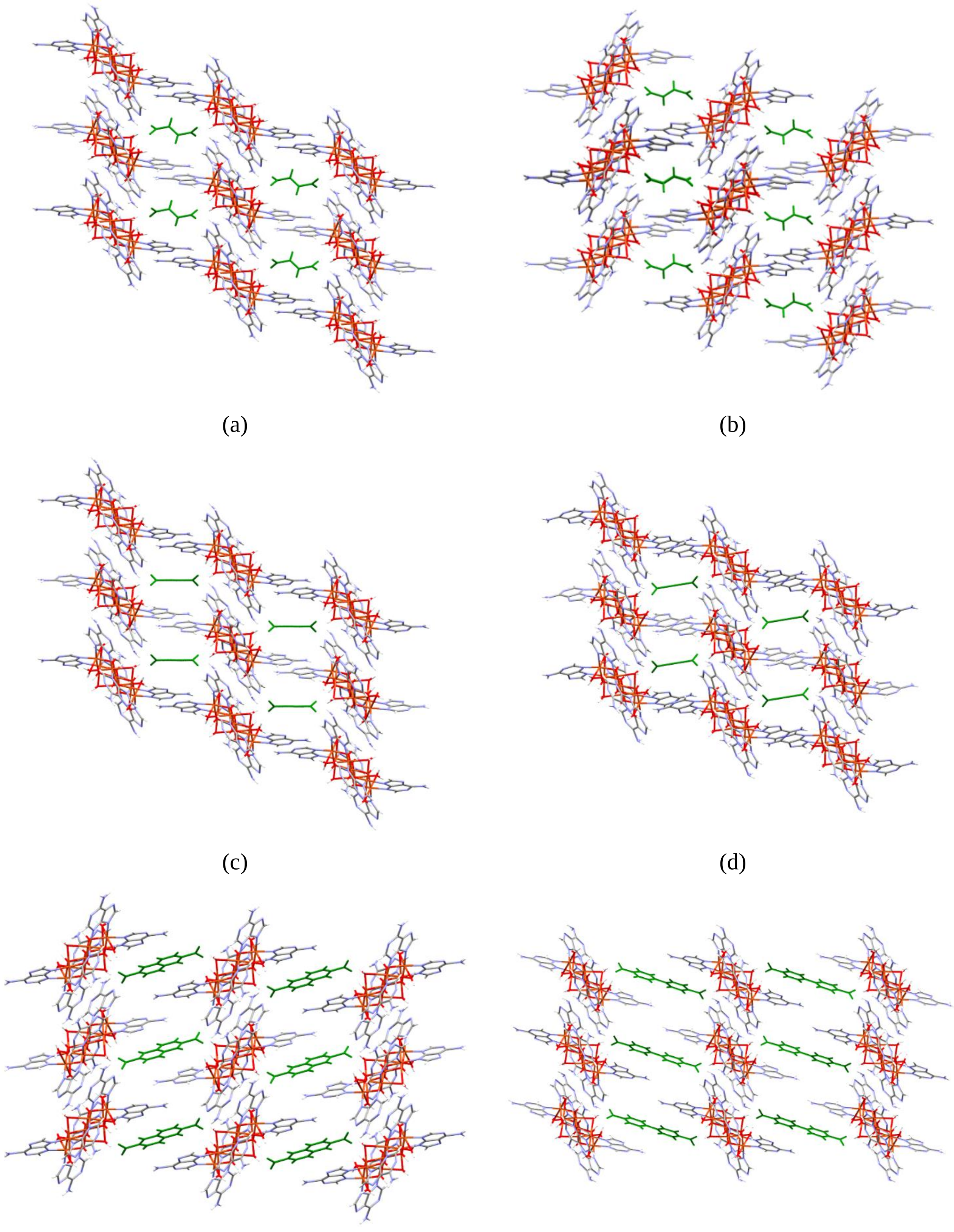

(a) (b) (c) (d) (e) (f)

Figure S10. View of the crystal packing of compounds: (a) **1a**, (b) **1b**, (c) **2a**, (d) **2b**, (e) **4a** and (f) **5** showing the insertion of the dicarboxylate dianions (green colour).

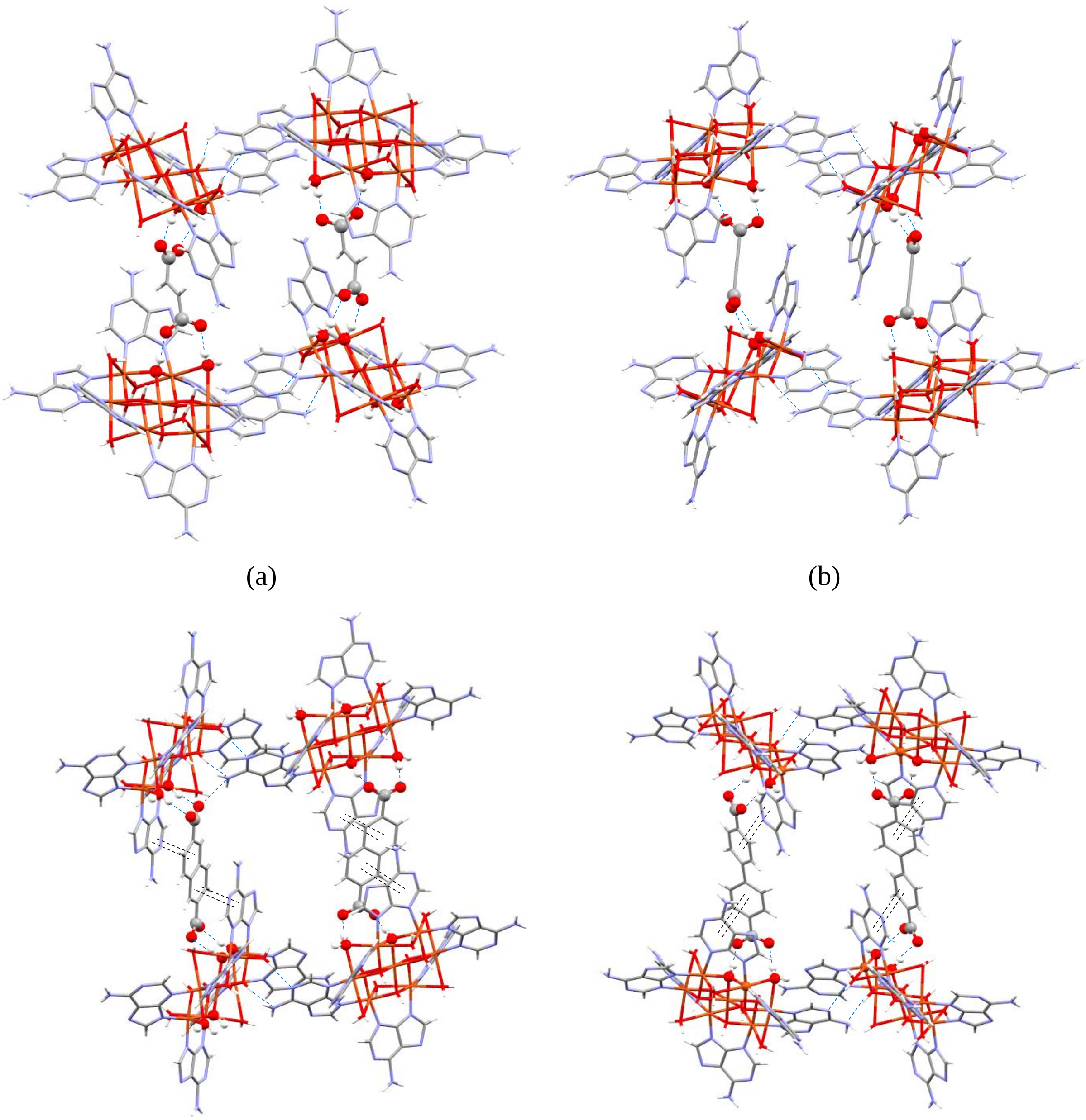

(a) (b) (c) (d)

Figure S11. Details of the supramolecular interactions between the organic anions and the cationic complexes in compounds: (a) **1a**, (b) **2a**, (c) **4a** and (d) **5**.

Table S11. Structural parameters (Å, deg) of hydrogen bonding and π-π stacking interactions in **1a**.[a]

**Hydrogen-bonding interactions**			
D−H···A[b]	H···A	D···A	D−H···A
O1−H1···O48	1.78	2.76(2)	171
O2−H2···N36A^{i}	1.92	2.90(2)	177
O3−H3···O4w^{ii}	1.76	2.74(1)	172
O1w−H12w···O5w	1.91	2.78(2)	171
O2w −H21w···O49	1.87	2.76(2)	174
O2w −H22w···O10w^{i}	2.05	2.85(2)	164
N16A−H16B···O9w^{iii}	1.77	2.62(3)	172
N26A−H26B···O10w^{iv}	2.18	3.04(2)	176

***π–π* interactions** [c]					
ring–ring[a]	packing	angle	DC	DZ	DXY
1pa····1pa^{i}	A····A	0.0	5.32	2.86	4.49
2pa····3pa^{v}	A····A	4.0	4.13	3.65	1.92

[a]Symmetry codes: (i) x, – y, 1/2 + z; (ii) – 1/2 – x, 1/2 + y,– 1/2 – z; (iii) – x, – y, – z; (iv) – 1/2 – x,– 1/2 + y,– 1/2 – z; (v) x, – y, 1/2 + z. [b]D: donor; A: acceptor. [c]Angle: dihedral angle between the planes (deg), DC: distance between the centroids of the rings (Å), DZ: interplanar distance (Å), DXY: lateral displacement (Å), pa: adenine pentagonal ring.

Table S12. Structural parameters (Å, deg) of hydrogen bonding and π-π stacking interactions in **1b**.[a]

**Hydrogen-bonding interactions**			
D−H···A[b]	H···A	D···A	D−H···A
O1−H1···O48	1.70	2.70(4)	172
O2−H2···N36^{i}	1.84	2.84(4)	179
O3−H3···O6w	1.72	2.70(3)	168
O1w−H11w···N31^{i}	2.12	2.93(4)	159
O1w −H12w···O5w^{ii}	1.97	2.86(2)	173
O2w−H21w···O49	1.74	2.55(4)	168
O2w−H22w···O7w^{iii}	2.23	3.01(3)	168
N26 −H26B···O7w ^{iv}	2.16	3.03(3)	171

***π–π* interactions** [c]					
ring–ring[a]	packing	angle	DC	DZ	DXY
1pa····1pa^{ii}	A····A	0.0	4.76	3.06	3.65
2pa····3pa^{i}	A····A	4.0	4.23	3.78	1.89

[a]Symmetry codes: (i) x, 1 – y, – 1/2 + z; (ii) – x, 1 – y, – z; (iii) – x, y,1/2 – z; (iv) 1/2 + x, 3/2 – y, 1/2 + z. [b]D: donor; A: acceptor. [c]Angle: dihedral angle between the planes (deg), DC: distance between the centroids of the rings (Å), DZ: interplanar distance (Å), DXY: lateral displacement (Å), pa: adenine pentagonal ring.

Table S13. Structural parameters (Å, deg) of hydrogen bonding and π-π stacking interactions in **2a**.[a]

**Hydrogen-bonding interactions**			
D−H···A[b]	H···A	D···A	D−H···A
O1–H1···O48	1.82	2.78(7)	168
O2–H2···N36[i]	1.93	2.91(8)	176
O3–H3···O4W[ii]	1.82	2.80(6)	174
O1W–H12W···N31[i]	2.03	2.88(7)	167
O2W–H21W···O49	1.88	2.73(8)	173
O2W–H22W···O6W	1.95	2.78(7)	172
N26A–H26B···O6W[iii]	2.22	3.07(2)	173
N36–H36B···O4W[iii]	2.27	3.15(1)	173

***π–π* interactions**[c]					
ring–ring[a]	packing	angle	DC	DZ	DXY
1pa····1pa[iv]	A····A	0.0	5.42	2.93	4.56
2pa····3pa[i]	A····A	4.6	4.09	3.64	1.94

[a]Symmetry codes: (i) x, – y, 1/2 + z; (ii) x, − y,– 1/2 + z; (iii) 1/2 − x, 1/2 − y, 1 − z; (iv) x, – y, 1 – z. [b]D: donor; A: acceptor. [c]Angle: dihedral angle between the planes (deg), DC: distance between the centroids of the rings (Å), DZ: interplanar distance (Å), DXY: lateral displacement (Å), pa: adenine pentagonal ring.

Table S14. Structural parameters (Å, deg) of hydrogen bonding and π-π stacking interactions in **2b**.[a]

**Hydrogen-bonding interactions**			
D−H···A[b]	H···A	D···A	D−H···A
O1–H1···O49[i]	1.79	2.64(2)	164
O2–H2···O6[ii]	1.89	2.81(2)	168
O3–H3···N26[iii]	1.83	2.70(3)	173
O1W–H11W···N21[iv]	2.14	3.01(1)	170
O1W–H12W···O4W	2.07	2.85(2)	166
O2W–H21W···O7W	2.07	2.87(2)	168
O3W–H31W···O48	1.78	2.72(2)	169
O3W–H32W···O8W	1.99	2.84(2)	163
N36A–H36A···O8W[i]	2.08	2.92(2)	163

***π–π* interactions**[c]					
ring–ring[a]	packing	angle	DC	DZ	DXY
1pa····1pa[v]	A····A	0.0	4.82	2.97	3.79
2pa····3pa[ii]	A····A	9.7	4.20	3.66	1.78

[a]Symmetry codes: (i) 3/2 – x, 1/2 – y, – z; (ii) x, – y, 1/2 + z; (iii) x, – y, – 1/2 + z; (iv) 3/2 – x, 1/2 + y, 1/2 – z; (v) 1 – x, 1 – y, z. [b]D: donor; A: acceptor. [c]Angle: dihedral angle between the planes (deg), DC: distance between the centroids of the rings (Å), DZ: interplanar distance (Å), DXY: lateral displacement (Å), pa: adenine pentagonal ring.

Table S15. Structural parameters (Å, deg) of hydrogen bonding and π-π stacking interactions in **4a**.[a]

**Hydrogen-bonding interactions**					
D–H···A[b]		H···A	D···A	D–H···A	
O1–H1···N36[i]		2.05	2.87(2)	163	
O2–H2···O48		1.66	2.63(1)	172	
O3–H3···O4w[ii]		1.83	2.81(1)	175	
O1W–H11W···049		1.86	2.70(1)	174	
O1W–H12W···O5w		1.94	2.78(1)	174	
O2W–H21W···N31[i]		1.93	2.78(2)	166	
O2W–H22W···O6w		1.97	2.81(1)	164	
O3W–H31W···O8w[iii]		2.06	2.91(1)	170	
O3W–H32W···O12w		2.08	2.92(2)	169	
N16A–H16A···O5w[iv]		2.12	2.97(2)	167	
***π–π* interactions**[c]					
ring–ring[a]	packing	angle	DC	DZ	DXY
1pa····3pa[ii]	A····A	6.4	4.28	3.64	2.25
2pa····1hd[v]	A····N	4.0	3.86	3.60	1.65
2pa····2hd[vi]	A····N	4.0	3.86	3.59	1.65

[a]Symmetry codes: (i) 2 – x, 1/2 + y, 5/2 – z; (ii) 2 – x, – 1/2 + y, 5/2 – z; (iii) x, – 1/2 – y, – 1/2 + z; (iv) 2 – x, 1 – y, 2 – z; (v) x, y, z; (vi) 3 – x, – y, 2 – z. [b]D: donor; A: acceptor. [c]Angle: dihedral angle between the planes (deg), DC: distance between the centroids of the rings (Å), DZ: interplanar distance (Å), DXY: lateral displacement (Å), pa: adenine pentagonal ring, hd: anion hexagonal ring.

Table S16. Structural parameters (Å, deg) of hydrogen bonding and π-π stacking interactions in **5**.[a]

**Hydrogen-bonding interactions**					
D–H···A[b]		H···A	D···A	D–H···A	
O1–H1···O5w		1.89	2.86(6)	171	
O2–H2···N16[i]		2.05	2.88(7)	164	
O3–H3···O48		1.73	2.70(6)	170	
O1w–H11w···O49[ii]		1.91	2.77(6)	179	
O1w –H12w···O7w[iii]		1.91	2.75(7)	171	
O2w–H21w···N11i		1.96	2.82(8)	170	
O2w–H22w···O6w		2.02	2.87(7)	169	
N36 –H36B···O7w		1.93	2.77(1)	165	
***π–π* interactions**[c]					
ring – ring[a]	packing	angle	DC	DZ	DXY
2pa····hd[iv]	A····B	3.9	4.04	3.57	2.04
1pa····3pa[vi]	A····A	5.2	4.21	3.66	2.14

[a]Symmetry codes: (i) 3/2 – x, 1/2 + y, 1 – z; (ii) 2 – x, – y, 1 – z; (iii) 2 – x, – 1 – y,1 – z; (iv) x, y, z; (vi) 1/2 + x, – 1/2 – y, z. [b]D: donor; A: acceptor. [c]Angle: dihedral angle between the planes (deg), DC: distance between the centroids of the rings (Å), DZ: interplanar distance (Å), DXY: lateral displacement (Å), pa: adenine pentagonal ring, hd: anion hexagonal ring.

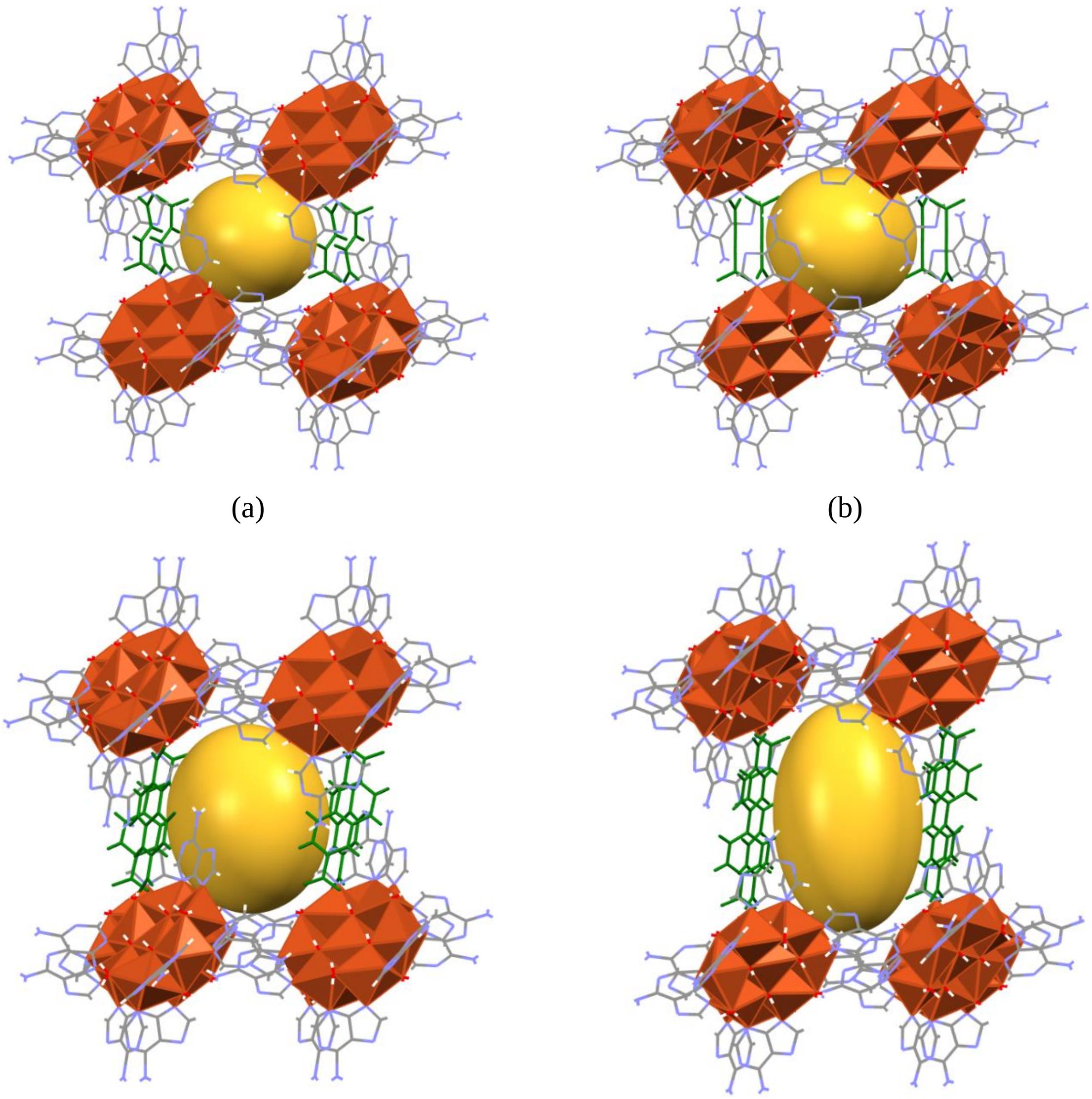

(a) (b)

(c) (d)

Figure S12. Supramolecular boxes present in the isoreticular crystal structure of compounds (a) **1a** (b) **2a**, (c) **4a** and (d) **5**.

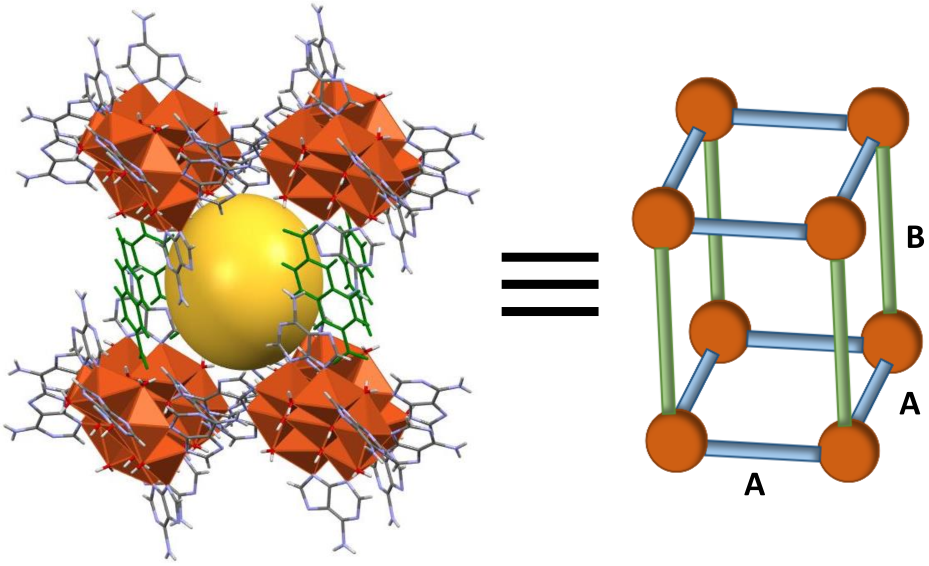

Figure S13. Main pore shape observed in these compounds defining the box like parameters.

Table S17. Porosity data for all reported compounds.

Compounds	A (Å)	B (Å)	d Pore (mode, Å) Min	max	Void volume (Å^3)	Void (%)	Surface area (m^2/g)	Pore volume (cm^3/g)
**1a**	12.2	13.5	3.6	6.6	2662	34	390	0.257
**1b**	12.0	13.5	3.2	5.8	2137	29	242	0.204
**2a**	12.2	13.8	3.5	6.7	2788	36	449	0.267
**2b**	12.1	13.0	2.9	5.7	2198	30	216	0.210
**4a**	12.1	18.6	3.5	8.1	1994	43	728	0.359
**5**	12.1	18.6	4.8	9.3	5380	50	1153	0.476

Geometric pore size distribution (PSD was computed by means of a Monte Carlo procedure implemented within a code developed by L. Sarkisov, in which the Lennard-Jones (LJ) universal force field parameters are used to describe the SMOF atoms while the accessible pore volume is assessed by a gradually increasing probe (Sarkisov, L.; Bueno, R.; Sutharson, M.; Fairen, D. Materials Informatics with PoreBlazer v4.0 and the CSD MOF Database. *Chem. Mater.* **2020**, *32*, 9849–9867).

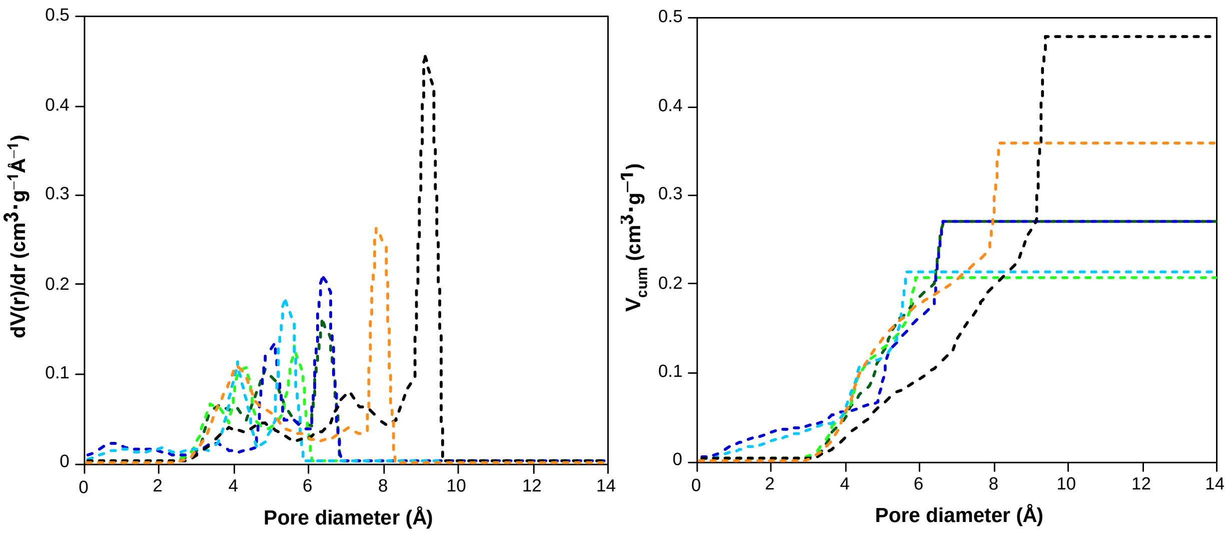

(a) (b)

Figure S14. (a) Derivative representation of the geometric pore volume of compounds (b) Cumulative representation of the geometric pore volume of compound **1a/1b** (dark and light green), **2a/2b** (dark and light blue), **4a** (orange) and **5** (black).

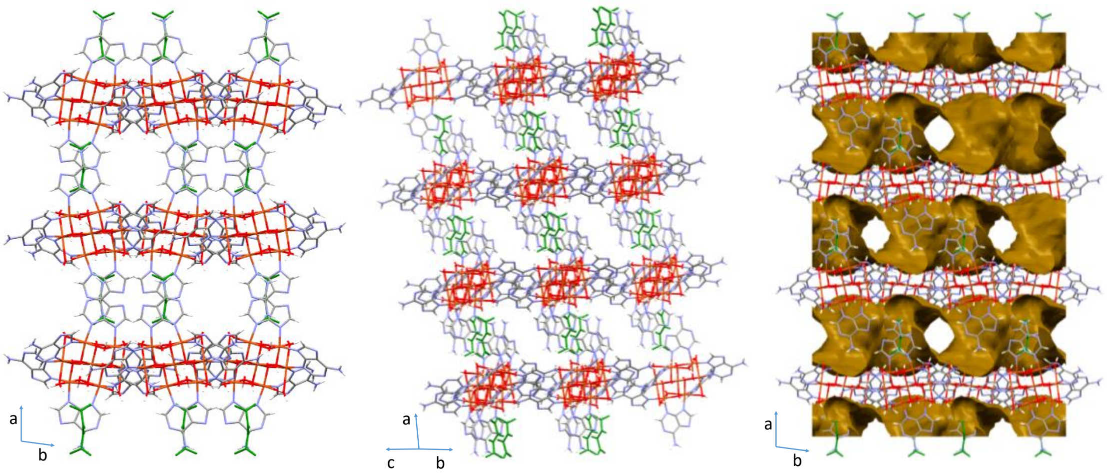

(a)

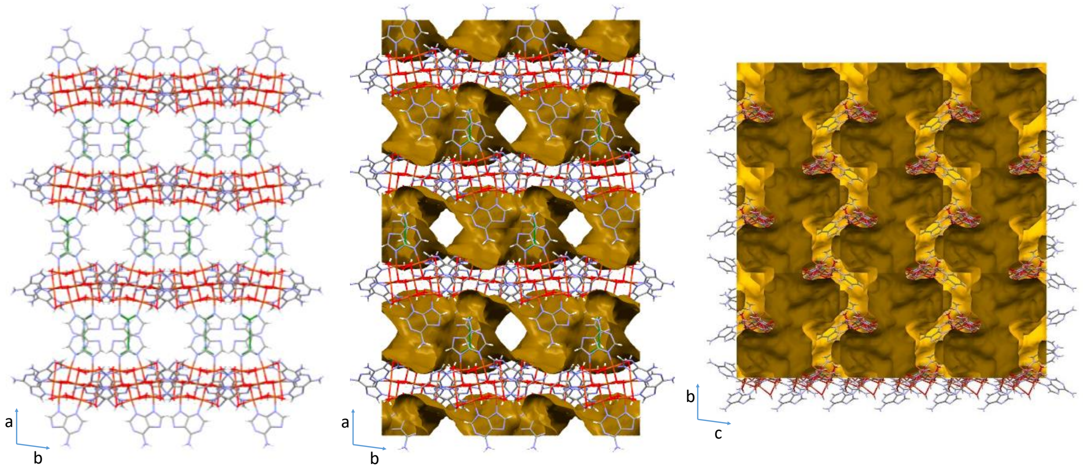

(b)

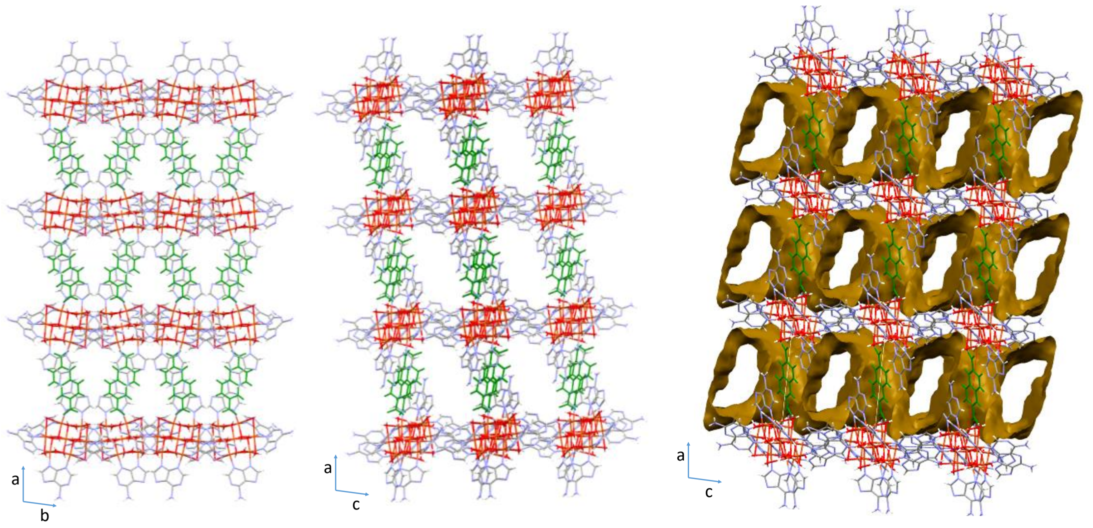

(c)

Figure S15. Image showing the channels for compounds: (a) **1a**, (b) **2a** and (c) **4a**.

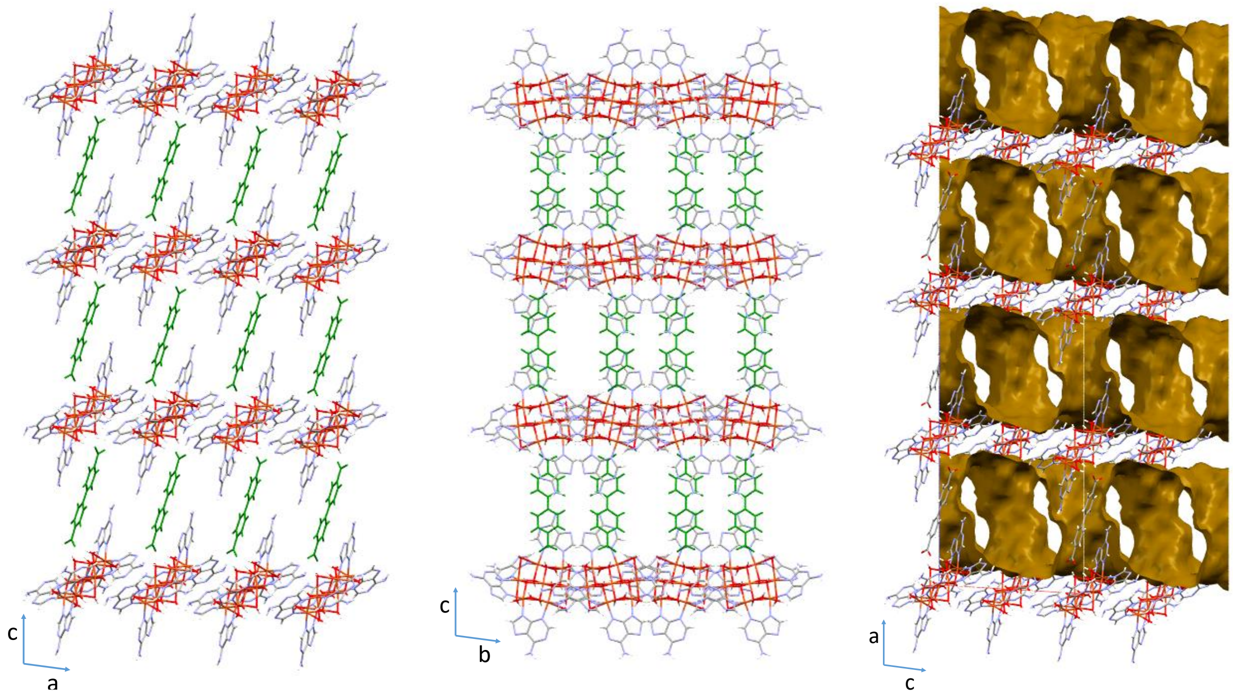

(a) (b) (c)

Figure S16. Views of the crystal structure of compound **5** (a, b) and the inner surface of the porous system (c).

S6. MAGNETIC ANALYSIS

Magnetization measurements as a function of field (up to 7 T) and temperature (range of 2 to 300 K) have been performed in a SQUID magnetometer (MPMS3, Quantum design. The crystals of **1a**, **2a**, and **4a** compounds lose molecules of water when extracted from their mother liquor and **1b**, **2b**, and **4b** compounds are partially dehydrated phases of the first ones. So that, the assays that require bulk samples were realized for these last compounds. Additionally, only a few crystals of compound **5** were obtained during the synthetic procedure and these assays could not be carried out on them.

The magnetic measurements of **1b**, **2b** and **4b** compounds indicate an overall ferrimagnetic behavior within the heptameric copper-adenine entity, in agreement with previous examples of compounds containing the same heptameric discrete entity.[1,2] Figures S17 and S18 show the magnetic behavior of these compounds. The values of $\chi_M T$ at 300 K is close to that corresponding to seven uncoupled Cu^{2+} atoms with S = 1/2 and g = 2.10 (2.89 $cm^3 \cdot K \cdot mol^{-1}$). The curve is essentially constant up to 150 K, below that temperature a sharp increase is observed. The magnetization curve at 2 K shows a linear dependence, from 0 to 12–15 kOe, which slowly tends to saturate to a value of 4.6-5.8 μB/heptamer at 20 kOe. These values agree with the presence of a central copper(II) metal center antiferromagnetically coupled to the six external ones to provide a S_T = 5/2. The magnetization curve shows no hysteresis, indicating that the magnetic contributions are limited to the molecular scale of the heptameric entity without providing a 3D ordering. Only at very low temperature in the $\chi_M T$ curve, it can be observed a slight decrease probably due to the presence of very weak antiferromagnetic interheptameric coupling mediated through the supramolecular interactions.

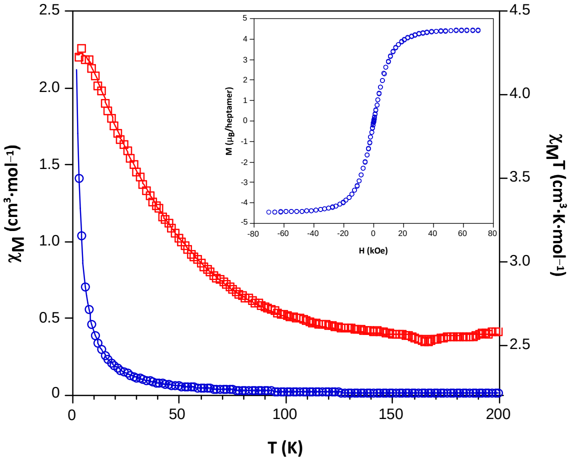

Figure S17. Thermal evolution of the molar magnetic susceptibility χ_M (o) and $\chi_M T$ (□) product for compound **1b**. Magnetization curve at 2 K (inset). The line shows the best fitting of the experimental data.

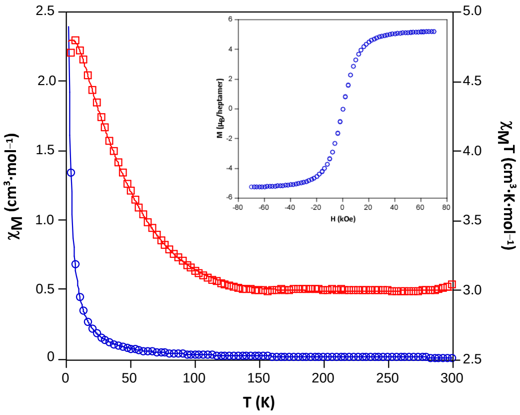

(a)

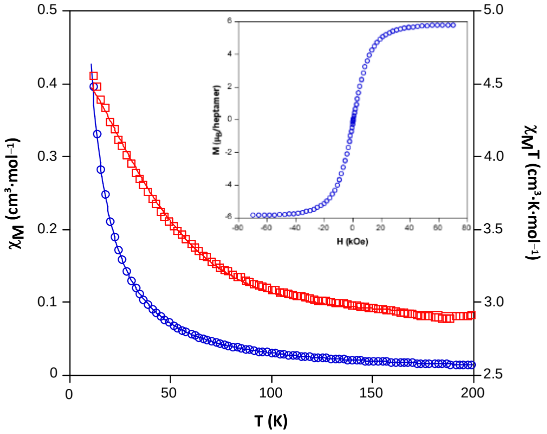

(b)

Figure S18. Thermal evolution of the molar magnetic susceptibility χ_M (o) and $\chi_M T$ (□) product for compound (a) **2b** and (b) **4b**. Magnetization curve at 2 K (inset). The line shows the best fitting of the experimental data.

In all compounds, we have defined three different external superexchange interactions (J), the first two fit the interaction involving the central atom and the peripheral copper atoms (J_1 and J_2). J_1 involves a mixture of equatorial–equatorial (short–short) and equatorial–axial (short–long) with the μ-OH bridges and J_2 involves double short (equatorial–equatorial) coordination bond distances with the μ-OH bridges. The last one, J_3 involves the peripheral Cu^{II} ions bridged by μ-adeninato, μ-OH_2 and μ_3-OH bridges and results in ferromagnetic values. As a consequence of the presence of these three magnetic pathways, a spin frustration situation arises as there is no optimal spin ordering within the heptameric entity that could fulfill all the magnetic interaction preferences. The relative strength of these pathways: $|J_2(\text{antiferromagnetic})| > |J_3(\text{ferromagnetic})| > |J_1(\text{ferromagnetic})|$ determines a $S_T = 5/2$ ground state as observed in its magnetization curve at 2 K.

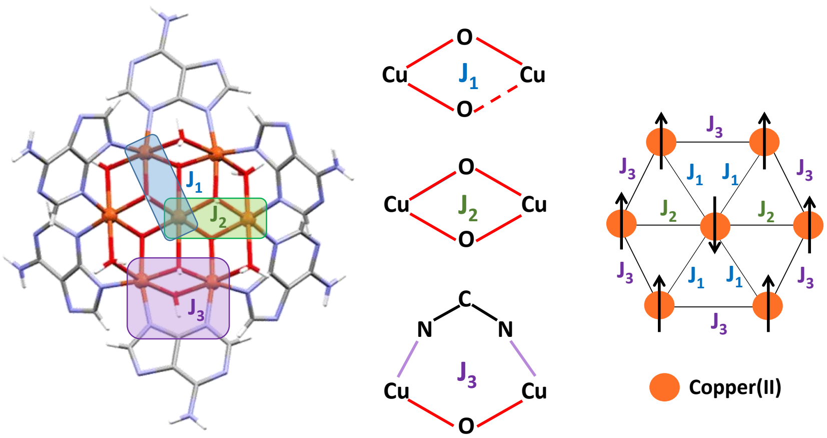

Figure S19. Magnetic superexchange scheme of the $[Cu_7(\mu\text{-adeninato})_6(\mu_3\text{-OH})_6(\mu\text{-OH}_2)_6]^{2+}$ heptanuclear entity emphasizing the different relationships of the magnetic topologies and three J coupling types for the local Cu^{II}_7 cluster.

Taking into account the molecular structure of the heptanuclear entity present in all compounds and the Jahn-Teller elongated octahedron of the central copper(II) atom, the following Hamiltonian [Eq. (S1)] was employed to fit the experimental data.

$$H = -J_1\left(\vec{S}_1\cdot\vec{S}_2 + \vec{S}_1\cdot\vec{S}_3 + \vec{S}_1\cdot\vec{S}_5 + \vec{S}_1\cdot\vec{S}_6\right) - J_2\left(\vec{S}_1\cdot\vec{S}_4 + \vec{S}_1\cdot\vec{S}_7\right) - J_3\left(\vec{S}_2\cdot\vec{S}_3 + \vec{S}_3\cdot\vec{S}_4 + \vec{S}_4\cdot\vec{S}_5 + \vec{S}_5\cdot\vec{S}_6 + \vec{S}_6\cdot\vec{S}_7 + \vec{S}_2\cdot\vec{S}_7\right) - g\mu_B\vec{B}\cdot\vec{S} \quad \text{(S1)}$$

J_1 and J_2 couplings are assigned to the superexchange interactions between the central and the external copper ions taking place through double μ-OH bridges. J_1 involves a mixture of short-short and short-long distances. J_2 presents only a short-short Cu-O based arrangement. J_3 represents the superexchange interaction between the external Cu^{II} ions. The fitting of the $\chi_M T$ experimental data to this model was performed using the PHI software tool.[3] The resulting magnetic coupling constants (Table S18) for the superexchange magnetic pathways depicted in Figure 7b are in concordance with previous reports.[1,2]

Table S18. Magnetic coupling constants (J, cm^{-1}) and g-factor values obtained from the fitting of the magnetic susceptibility curves.

**Compound**	J_1	J_2	J_3	g
**1b**	+76	–278	+97	2.04
**2b**	+44	–229	+97	2.10
**4b**	+27	–178	+94	2.06

References:

(1) Pérez-Aguirre, R.; Beobide, G.; Castillo, O.; de Pedro, I.; Luque, A.; Pérez-Yáñez, S.; Rodríguez Fernández, J.; Román, P. 3D Magnetically Ordered Open Supramolecular Architectures Based on Ferrimagnetic Cu/Adenine/Hydroxide Heptameric Wheels. *Inorg. Chem.* **2016**, *55*, 7755–7763.

(2) Pascual-Colino, J.; Beobide, G.; Castillo, O.; da Silva, I.; Luque, A.; Pérez-Yáñez, S. Porous Supramolecular Architectures Based on π-Stacking Interactions between Discrete Metal-Adenine Entities and the Non-DNA Theobromine/Caffeine Nucleobases. *Cryst. Growth Des.* **2018**, *18*, 3465–3476.

(3) Chilton, N.; Anderson, R.; Turner, L.; Soncini, A.; Murray, K. PHI: A Powerful New Program for the Analysis of Anisotropic Monomeric and Exchange-Coupled Polynuclear d- and f-Block Complexes. *J. Comput. Chem.* **2013**, *34*, 1164–1175.

S7. WATER ADSORPTION AND HUMIDITY SENSOR

Water vapour sorption isotherms were performed using an automated gravimetric analyzer (Aquadyne DVS, Quantachrome Instruments) with nitrogen 6.0 as carrier gas. The weight of the sample is constantly monitored and recorded as the relative humidity is automatically varied by the blending of dry carrier gas with a saturated gas stream using precision mass flow controllers. An equilibrium criterion corresponding to 0.0004% of mass change per minute at a given relative humidity was used. Before the experiments, the samples were outgassed under vacuum at 30°C for 8 hours.

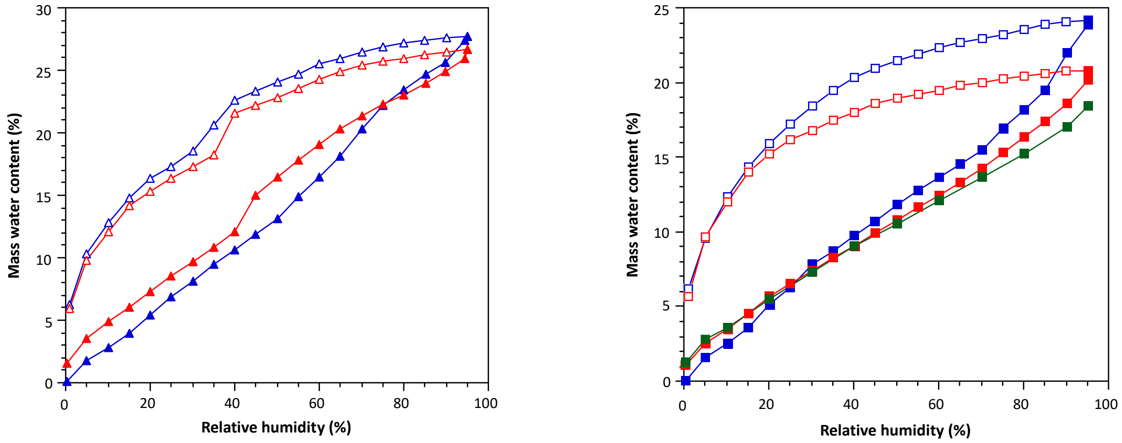

(a) (b)

Figure S20. Consecutive adsorption/desorption cycling (1st cycle: blue dots; 2nd cycle: red dots and 3rd cycle: green dots) at 20 ºC for (a) **1** and (b) **2**.

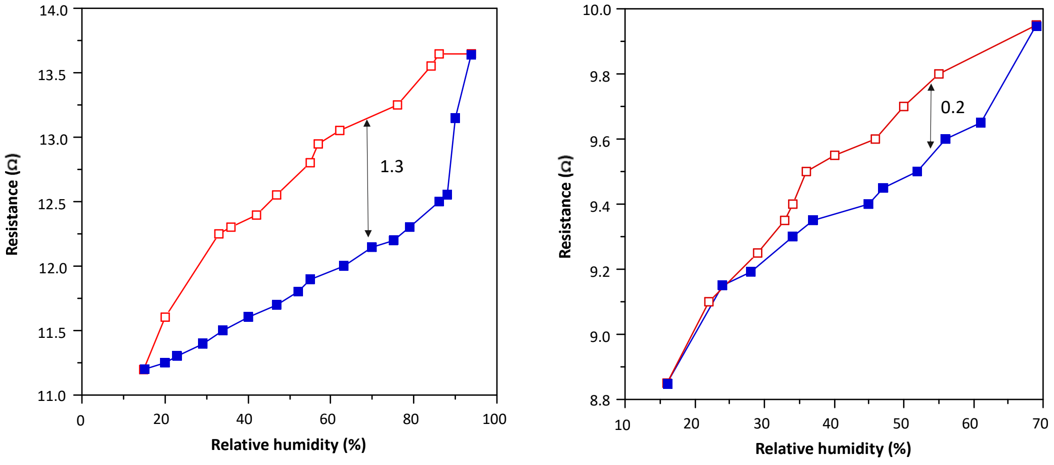

(a) (b)

Figure S21. Electrical resistance measurements under different relative humidity conditions of the disk shaped pellet for compound **2**: (a) from 15 to 95%, (b) from 15 to 70%. Filled blue and empty red points correspond to the humidity increase and decrease stages, respectively. Different pellets were employed for cycling (a) and (b).

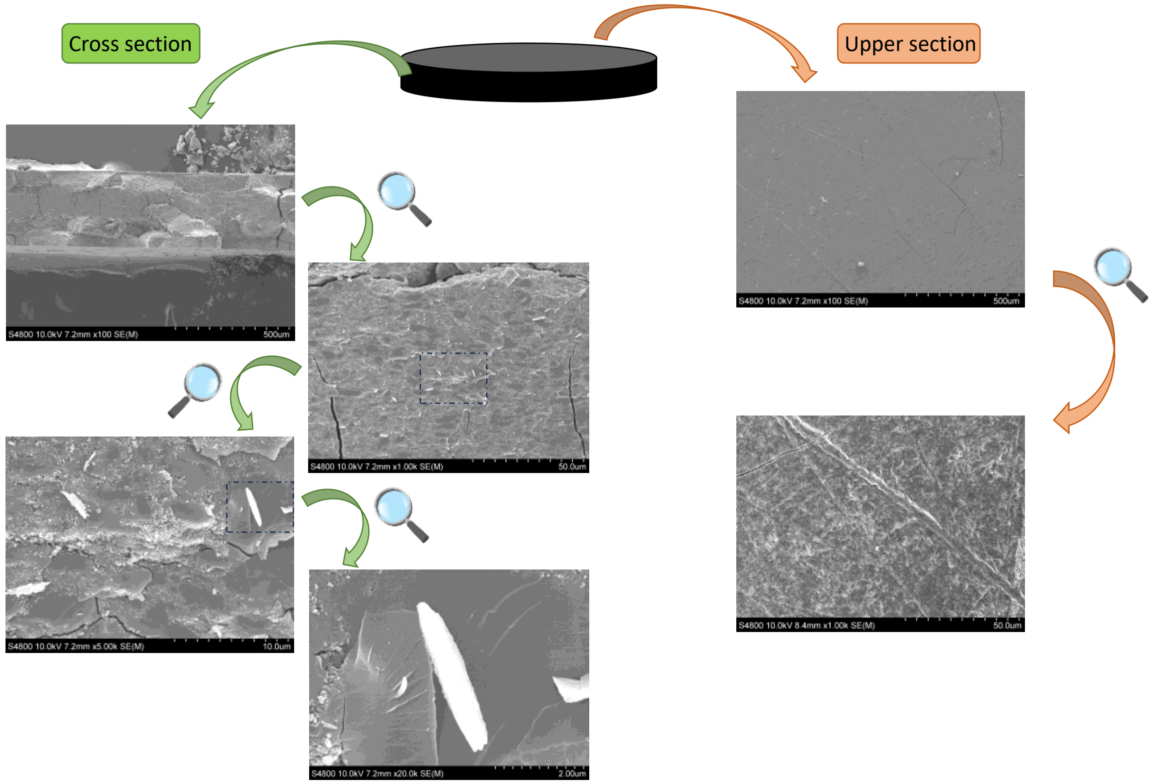

Figure S22. SEM images of the sensing composite pellet.

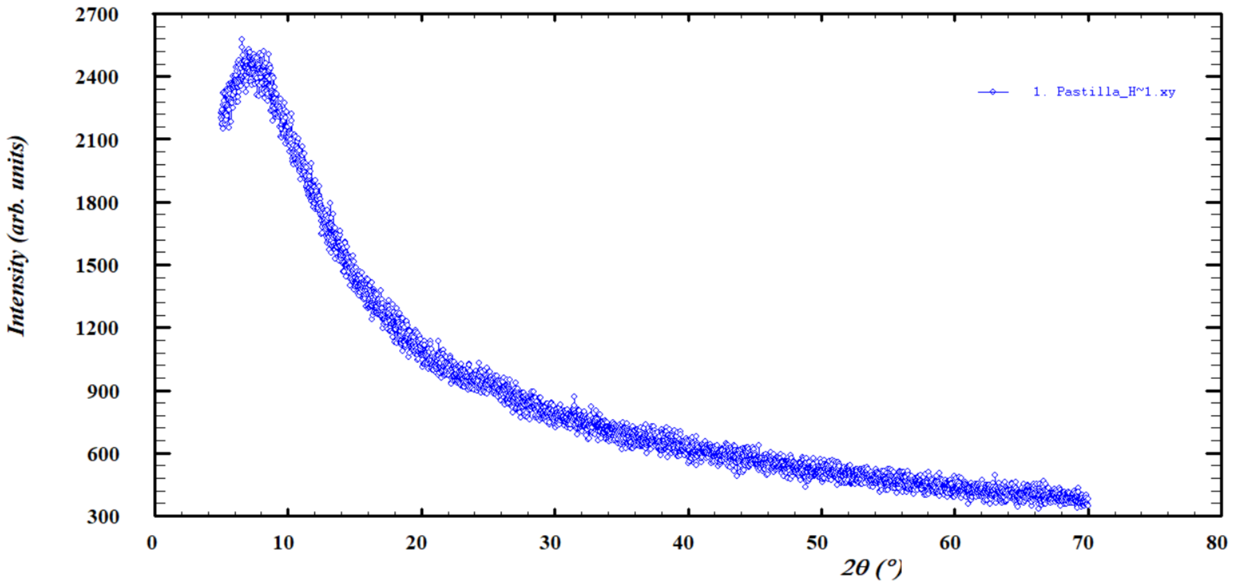

Figure S23. X-ray diffraction pattern of the sensing composite pellet.

S8. MAGNETIC SUSTENTATION METHOD

The method used to determine the amount of loaded molecules by a paramagnetic porous material in solution, developed by our research group, has been reported in previous works: (a) Pérez-Aguirre, R.; Artetxe, B.; Beobide, G.; Castillo, O.; de Pedro, I.; Luque, A.; Pérez-Yáñez, S.; Wuttke, S. "Ferromagnetic supramolecular metal-organic frameworks for active capture and magnetic sensing of emerging drug pollutants". *Cell Reports Phys. Sci.* **2021**, *2*, 1–13. (b) Barroso, N.; Andreo, J.; Beobide, G.; Castillo, O.; Luque, A.; Pérez-Yáñez, S.; Wuttke, S. "Magnetic sustentation as an adsorption characterization technique for paramagnetic metal-organic frameworks". *Commun. Chem.* **2023**, *6*, 4. (c) Pascual-Colino, J.; Pérez-Aguirre, R.; Beobide, G.; Castillo, O.; de Pedro, I.; Luque, A.; Mena-Gutiérrez, S.; Pérez-Yáñez, S. "An in solution adsorption characterization technique based on the response to an external magnetic field of porous paramagnetic materials: application on supramolecular metal–adenine frameworks containing heterometallic heptameric clusters". *Inorg. Chem. Front.* **2023**, *10*, 2250–2261.

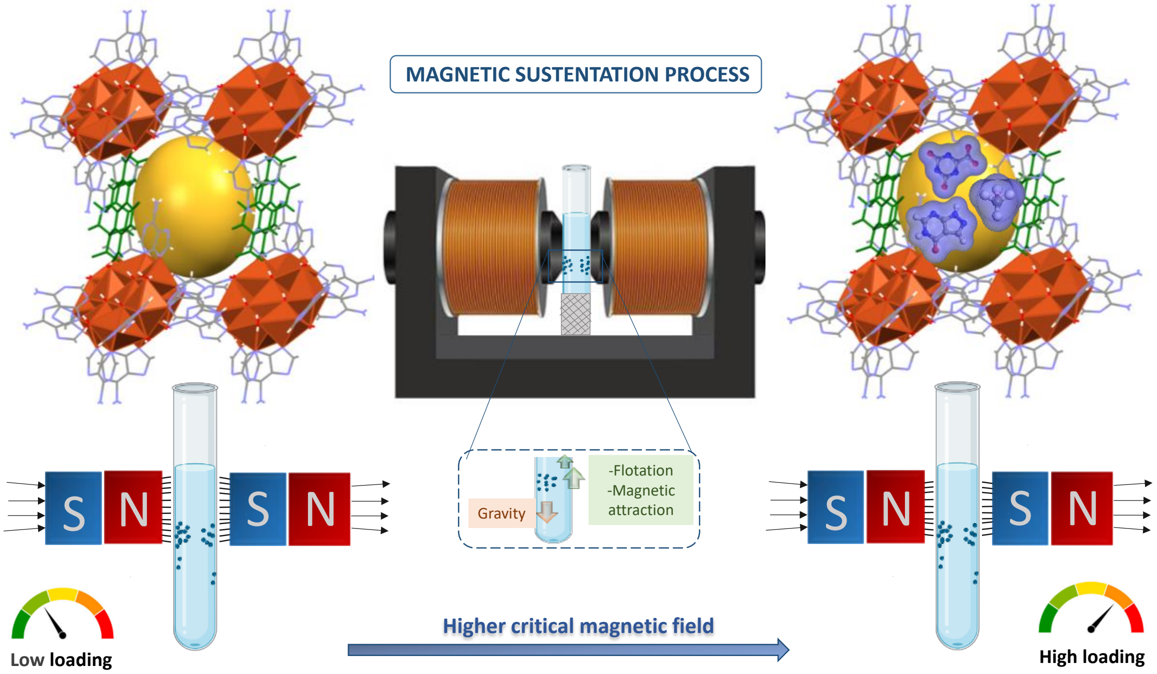

Figure S24. Scheme of the magnetic sustentation method

S9. SORPTION DATA QUANTIFICATION BY PROTON NUCLEAR MAGNETIC RESONANCE (^{1}H-NMR)

^{1}H–NMR spectra were acquired in a Bruker AVANCE 5OO (one-bay; 500 MHz) at 293 K. The adsorption experiment was repeated but using D_2O: 50 mg of compound **4b**, 2 mL of deuterated water and 50 mg or 50 μL of the adsorptive of interest (isopropanol, glucose and metanol) in each case. The samples were left under continuous agitation for 24h under room temperature (25 ºC). Later, the suspension is centrifuged to separate the solid from the solution and to 1 mL of the liquid phase 50 μL of a 5% *t*-butanol heavy water solution were added. The same procedure was applied for each adsorptive but without adding the porous material (**4b**) in order to set the initial adsorptive amount in the adsorption experiment. ^{1}H–NMR measurement was performed on the centrifuged solution and the characteristic signals of the adsorptive and *t*-butanol were employed to quantify the amount of the adsorptive remaining in solution and by difference with the initial value to determine the amount adsorbed within the porous material. Figures show the blank and after the sorption experiment solution ^{1}H–NMR spectra, indicating the signals employed for the quantification.

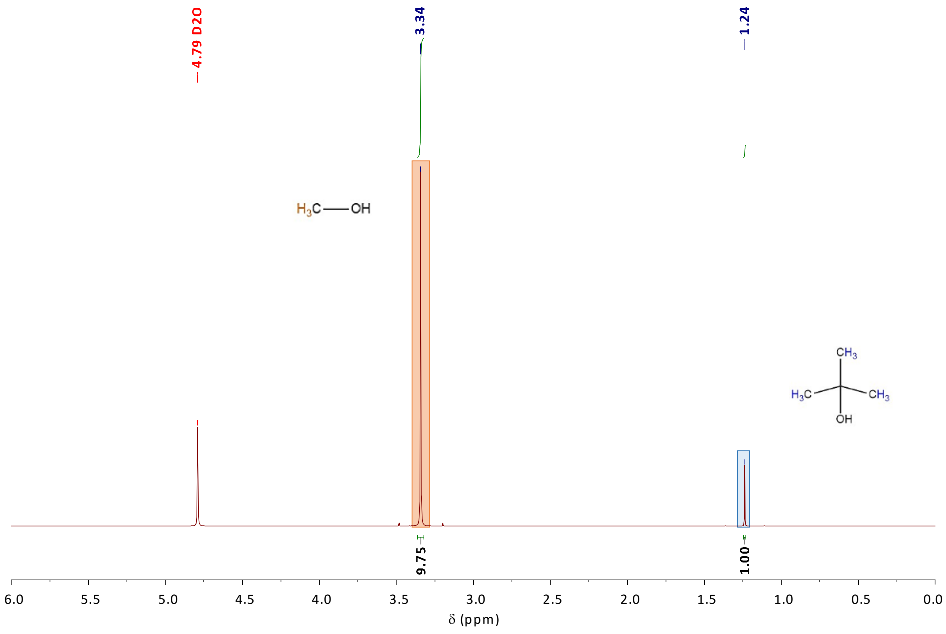

(a)

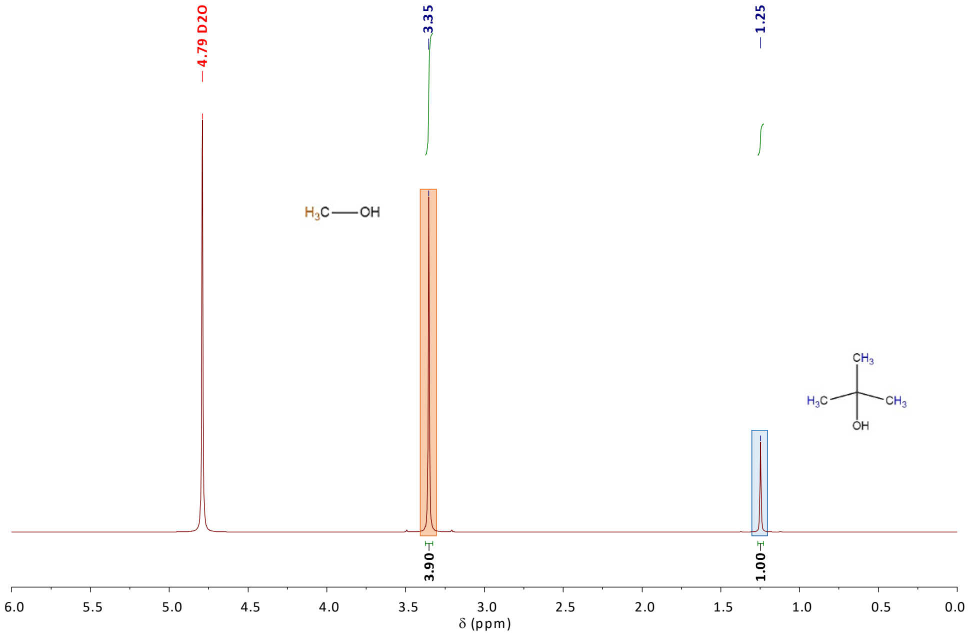

(b)

Figure S25. ^{1}H-NMR spectra for blank (upper) and experimental (bottom) samples of compound **4** with methanol**.**

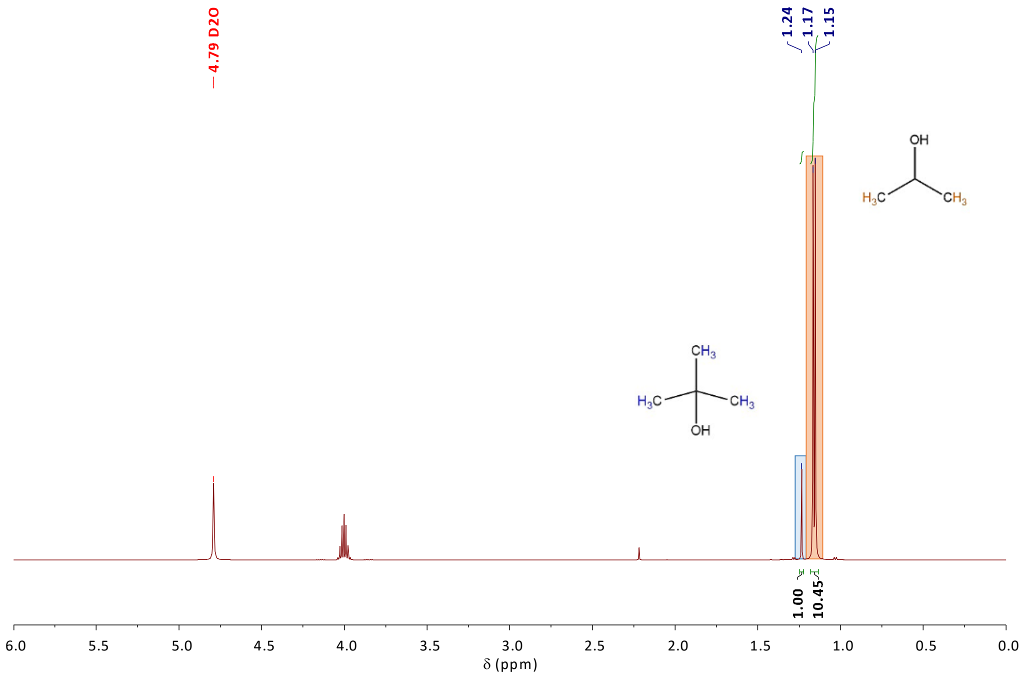

(a)

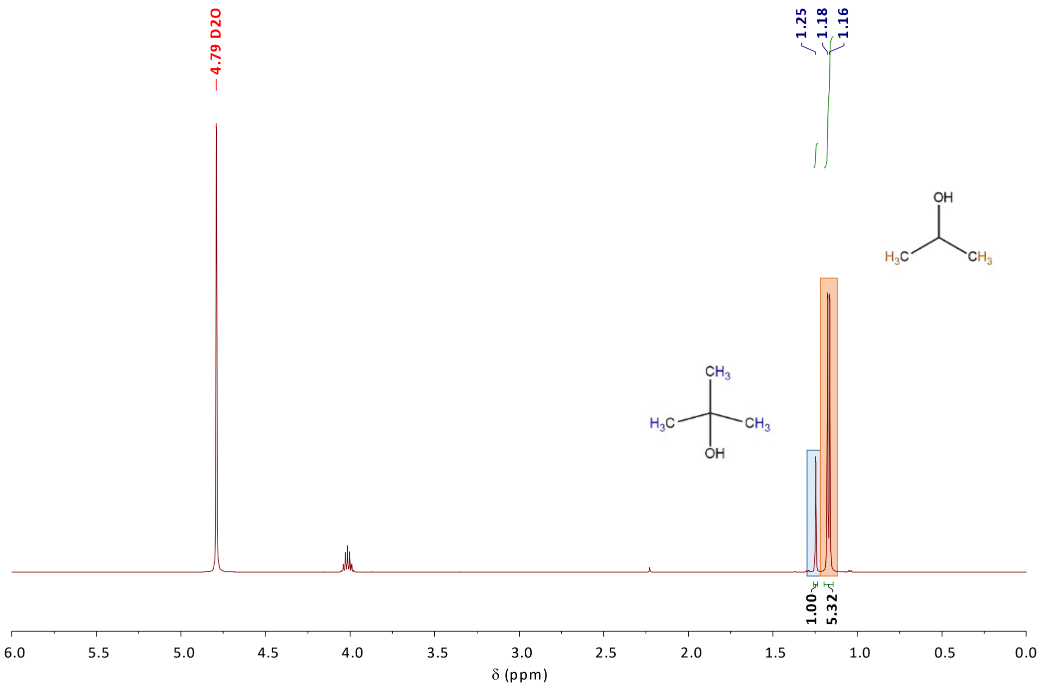

(b)

Figure S26. ^{1}H-NMR spectra for blank (upper) and experimental (bottom) samples of compound **4** with isopropanol**.**

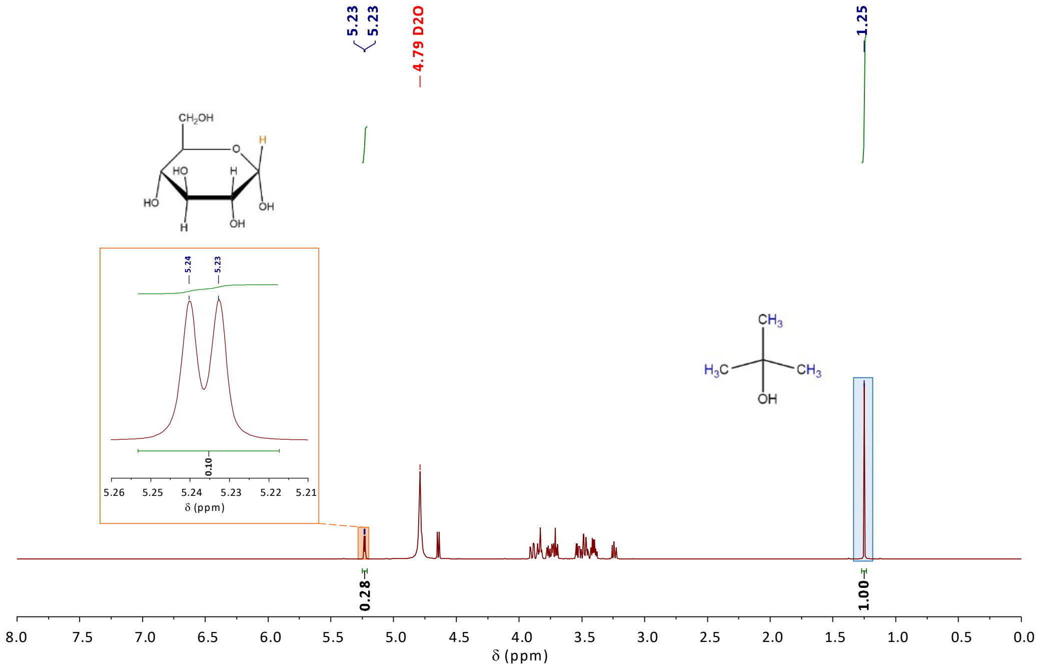

(a)

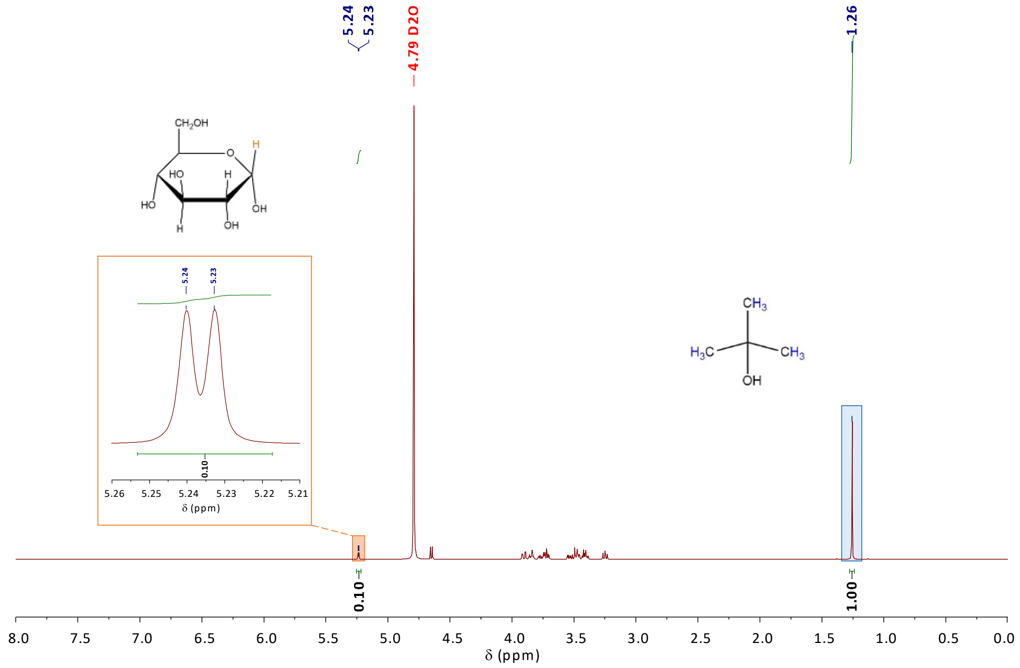

(b)

Figure S27. ^{1}H-NMR spectra for blank (upper) and experimental (bottom) samples of compound **4** with glucose**.**

S10. POWDER X-RAY DIFFRACTION PATTERNS AND FTIR SPECTRA OF THE DRUG LOADED COMPOUND 4

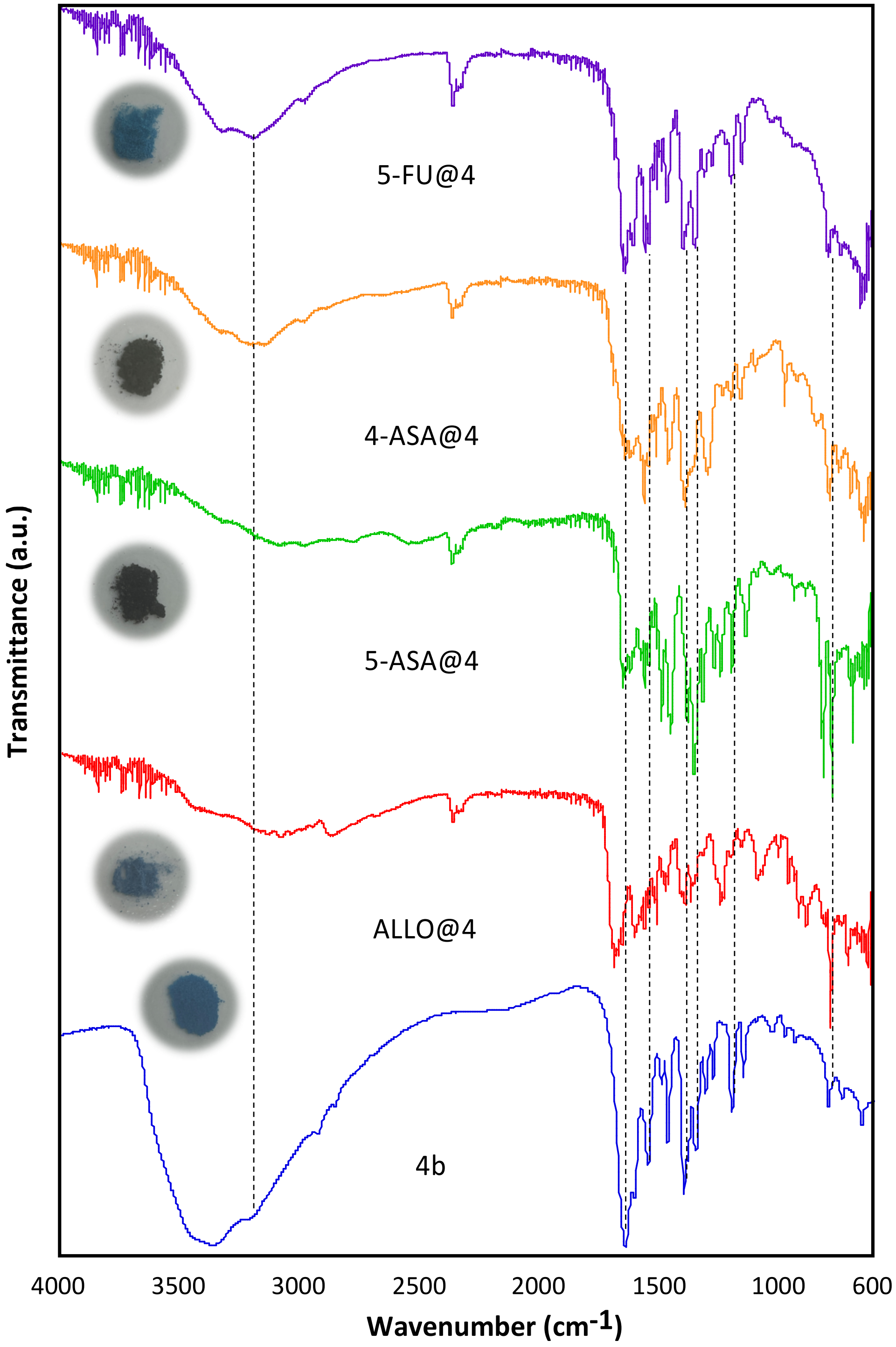

Figure S28. FTIR of compound **4** after filtration (**4b**), loaded with allopurinol (**ALLO@4**), 5-aminosalicylic acid (**5-ASA@4**), 4-aminosalicylic acid (**4-ASA@4**) and 5-fluorouracil (**5-FU@4**).

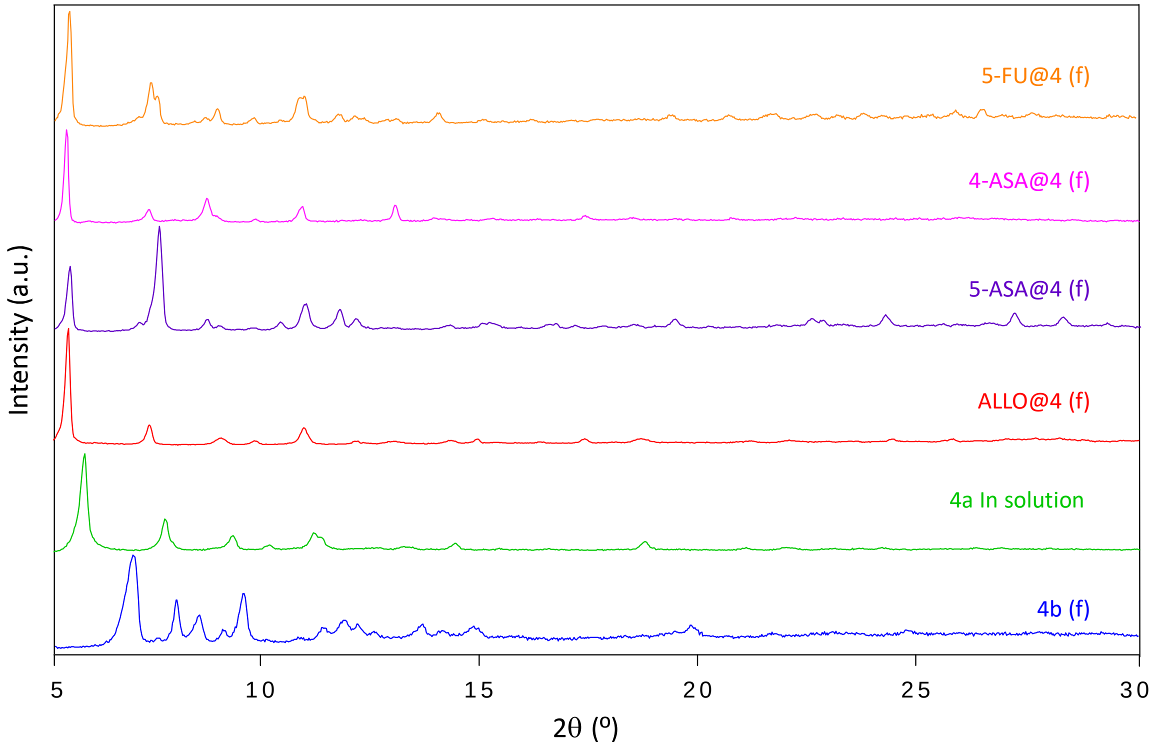

Figure S29. Powder diffraction pattern of compound **4** while in solution (**4a**, in solution), after filtration (**4b**), loaded with allopurinol (**ALLO@4**), 5-aminosalicylic acid (**5-ASA@4**), 4-aminosalicylic acid (**4-ASA@4**) and 5-fluorouracil (**5-FU@4**). (f) means after filtration.

S11. ADSORPTIVE VOLUMEN AND SHAPE

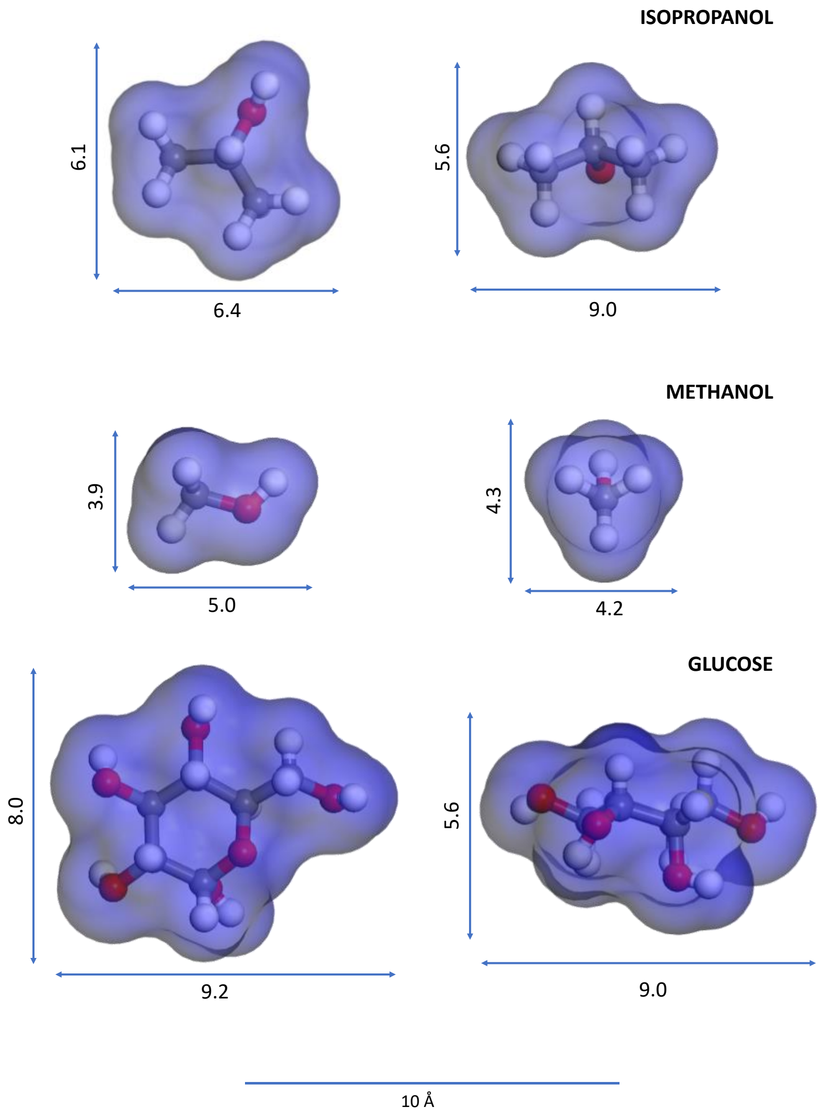

Figure S30. Shape and dimensions of the adsorptive molecules used for the magnetic sustentation technique calibration. The same scale has been used for all the images to facilitate a direct visual comparison between them. A 1.2 Å probe has been employed to define the Connolly surface of the adsorptive molecules (Materials Studio, *BIOVIA Materials Studio*: 2017 R2, 17.2.0.1626, package).

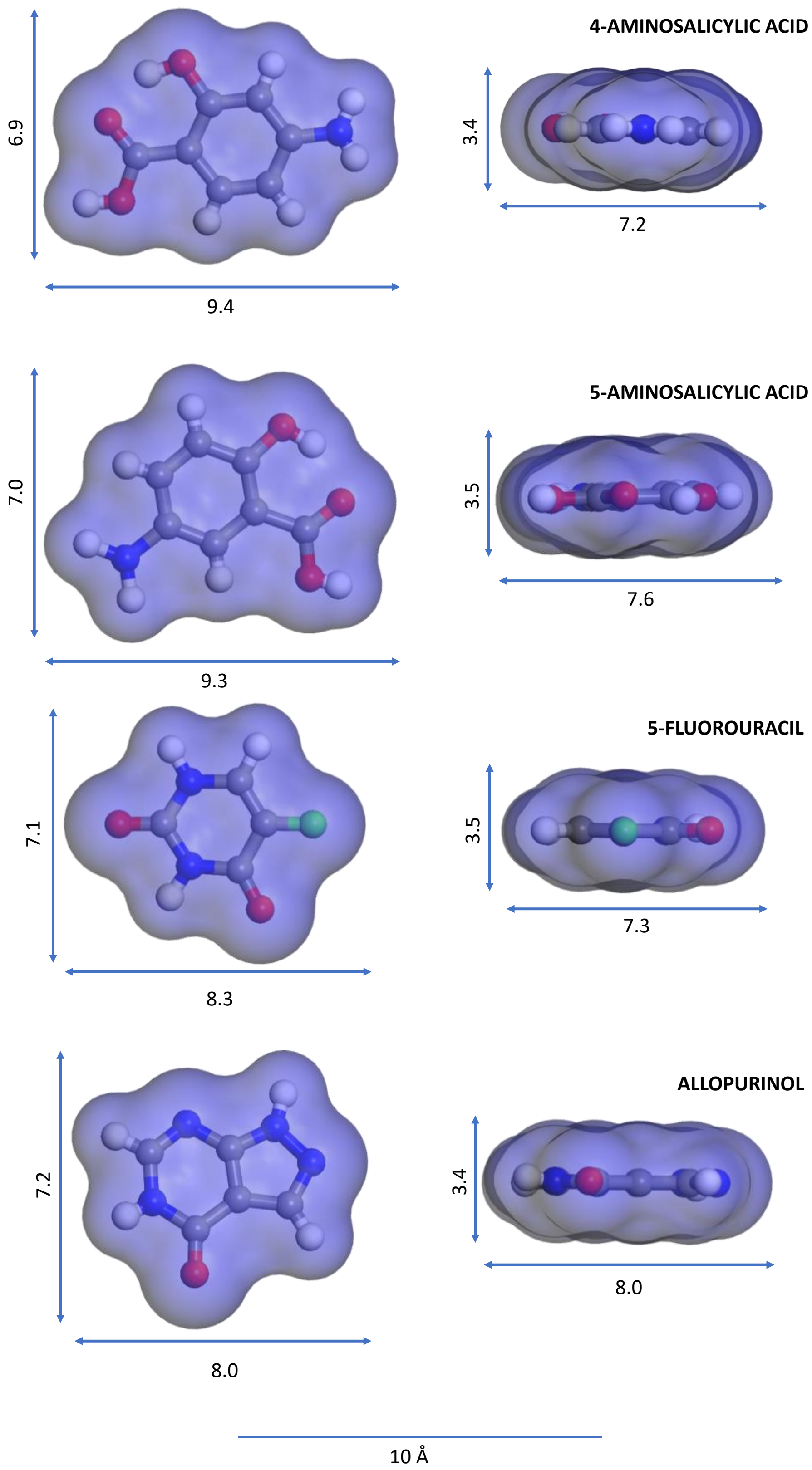

Figure S31. Shape and dimensions of the drug molecules.

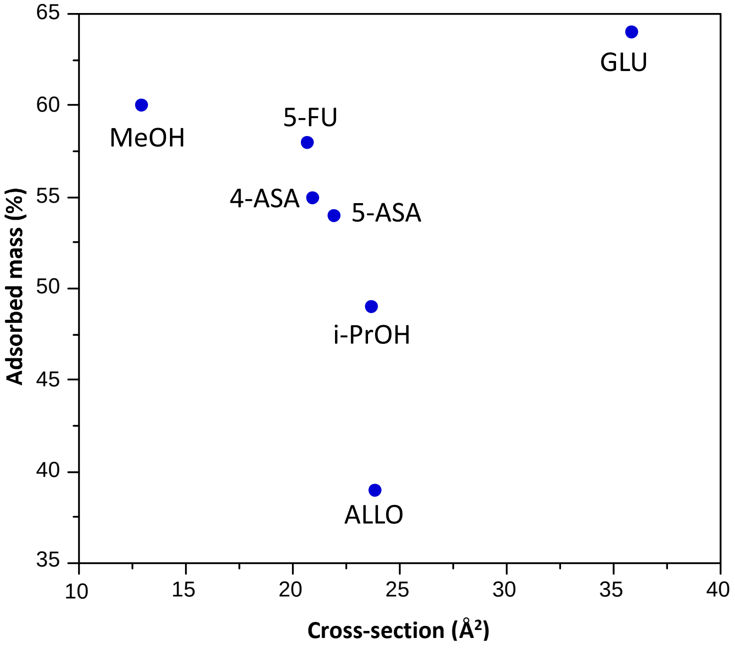

(a)

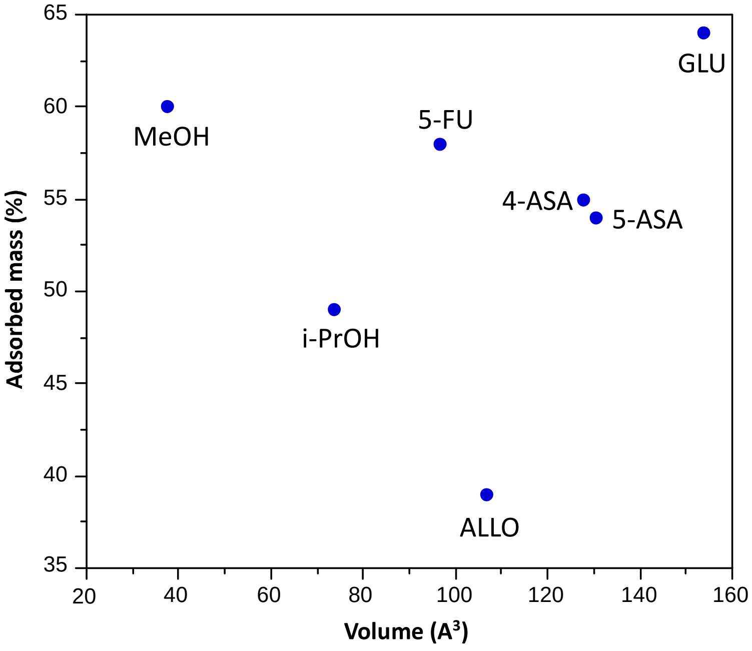

(b)

Figure S32. Absorbed drug mass percentage *vs* the adsorptive molecular cross-section (a) and (b) volume.

S12. CYTOTOXICITY ASSAYS

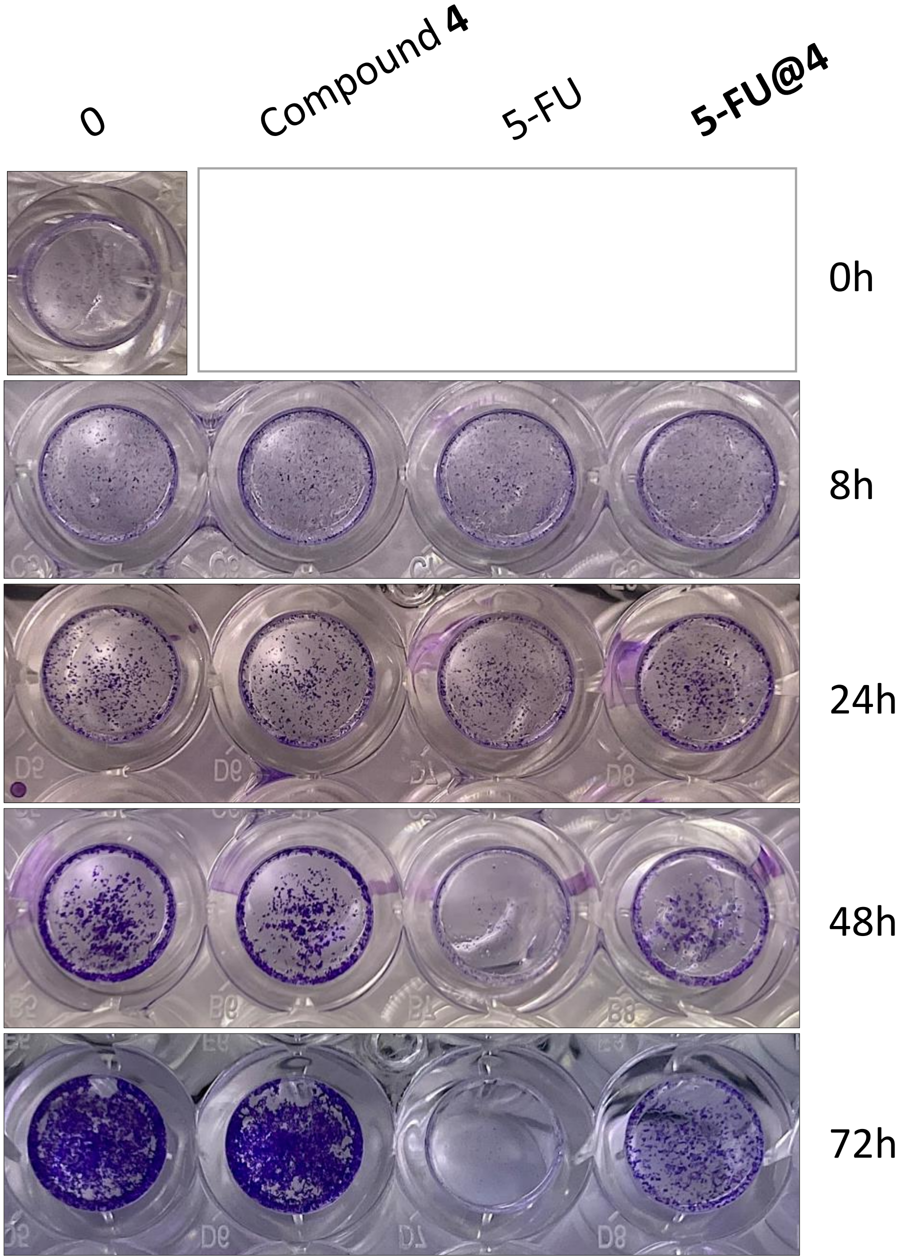

Figure S33. Crystal violet staining of HCT116 cells grown for 8h, 24h 48h and 72h in basal conditions (0) and in the presence of the different compounds. Image is representative of 3 independent experiments.
